# A Bayesian analysis of variables causally associated with hair cortisol concentration in dogs with obesity

**DOI:** 10.3389/fvets.2025.1695345

**Published:** 2025-11-27

**Authors:** Kaitlin Turnbull, Georgiana R. T. Woods-Lee, John Flanagan, Xavier Langon, Alexander J. German

**Affiliations:** 1 Institute of Infection, Veterinary and Ecological Sciences, University of Liverpool, Neston, United Kingdom; 2 Institute of Life Course and Medical Sciences, University of Liverpool, Neston, United Kingdom; 3 Royal Canin Research Centre, Aimargues, France

**Keywords:** overweight, adipose tissue, canine, chronic stress, causal inference, hypothalamic-pituitary-adrenal axis upregulation

## Abstract

**Objective:**

To identify clinical variables causally associated with hair cortisol concentration (HCC) in dogs with obesity using a Bayesian analysis.

**Study design:**

A retrospective analysis of clinical data and samples gathered from a cohort of dogs with obesity undergoing therapeutic weight reduction.

**Methods:**

Hair was clipped from two sites (jugular groove, antebrachium), from dogs attending a specialist obesity care clinic, and combined before storage at −20 °C. Hair cortisol concentration was measured by liquid chromatography mass spectrometry. Causal associations between HCC and different clinical variables were assessed, informed by a directed acyclic graph. Variables assessed included age, sex, breed group, coat colour, body fat mass, weight reduction and the presence of comorbidities. Statistical analyses involved Bayesian multi-level modelling, with the magnitude of causal effects estimated using simulation from the posterior probability distributions.

**Results:**

In total, 73 hair samples were collected from 52 dogs, with 31 providing single (before weight reduction) and 21 providing paired samples (before and after weight reduction). Dogs were of different ages, sexes and breeds, with most (44/52) having one or more comorbidities including orthopaedic, skin, cardiorespiratory, dental and neoplastic diseases. Mean HCC was 10.4 (standard deviation 19.52) pg/mg (logHCC 1.3, standard deviation 1.36). Bayesian multi-level models provided strong evidence that greater body fat percentage (98% probability) and presence of one or more comorbidities (>99% probability) were causality associated with increased HCC. Causal associations with other variables including, age, breed, sex, coat colour and season were less convincing.

**Conclusion:**

Greater adiposity and having at least one comorbidity are causally associated with hypothalamic–pituitary–adrenal axis upregulation in dogs with obesity. Mechanisms warrant further investigation.

## Introduction

Cortisol, the product of hypothalamic–pituitary–adrenal (HPA) axis activation, is a key hormone released in response to stress (1). Whilst acute cortisol concentrations can be measured in blood, saliva, or urine, such concentrations can be are influenced by circadian rhythms and acute stress at sampling (2, 3). Since cortisol is gradually incorporated into the keratinizing shaft during hair growth, hair cortisol concentrations (HCC) reflect its cumulative secretion over a period of weeks to months (1). Therefore, HCC has emerged as a non-invasive biomarker of HPA upregulation in both human and veterinary research (1, 2, 4). The method has been validated in dogs, showing good correlation between HCC and salivary cortisol concentration, and it has been applied to evaluate sustained cortisol output in various conditions (2, 4). For example, HCC is increased in dogs (5) and people (6) with spontaneous hyperadrenocorticism. Measurement of HCC is also used as a marker of possible stress in dogs experiencing different lifestyles or environments, such as working dogs and in dogs subjected to prolonged stressors in experimental settings (7). Therefore, HCC has the potential to capture chronic HPA axis upregulation stemming from both external and internal stressors. However, a standardised sampling protocol is required to ensure representative HCC measurements, controlling for possible confounding factors including coat colour, sampling season and region of the body from which hair is taken (8).

Chronic illness can also induce HPA upregulation in various ways including response to infection, chronic pain and due to altered immune function (9, 10). Obesity is a prevalent chronic disease in pet dogs, being associated with multiple comorbidities (11), a poorer quality of life (12), a shortened average lifespan (13) and also both functional and metabolic disturbances (14–17). Therefore, it is plausible that there may be HPA upregulation in dogs as a consequence of obesity. Indeed, in humans, there are positive associations between HCC and obesity metrics, including body weight, body-mass index and central fat distribution (18). In one weight maintenance trial, baseline HCC was associated with body mass in some cohorts, whilst increased HCC over 12 months predicted greater subsequent weight variability (19). One previous study has measured HCC in dogs with obesity, with little difference seen before and after a short weight reduction intervention involving lead walking (20); however, HCC tended to be greater in dogs undertaking the most exercise, possibly suggesting HPA upregulation due to physiological stress.

The term ‘causal inference’ is used to describe a process of analysing data to draw conclusions about causal relationships, and has most applicability when controlled experiments, including randomised control trials (RCT), are either impractical or unethical (21). Whereas traditional epidemiological methods focus on associations amongst variables, the aim of causal inference is to disentangle true causal effects from spurious correlation, including from confounding or bias. Although not yet fully exploited in canine research, causal inference has been used to assess risk factors for small intestinal dehiscence after surgery (22), unsuccessful dog ownership (23), early-onset incontinence (24) and patterns of physical activity (25).

Bayesian statistical analysis is an inferential framework based on Bayes’ theorem, in which prior knowledge or beliefs are updated after considering newly-observed data, enabling the probability of parameters or hypotheses of interest to be estimated (26). A crucial distinction from the frequentist approach is to consider the entire (posterior) probability distribution of an unknown quantity of interest, thereby better accommodating uncertainty in scientific research. Bayesian methods are particularly useful when sample sizes are small, when complex modelling is required (for example, hierarchical models) and when the degree of uncertainty needs to be quantified (27). Challenges with the computational complexity of Bayesian methods have recently been overcome by the development of Markov Chain Monte Carlo (MCMC) methods and availability of freely available online statistical software such as R (28); therefore, the use of Bayesian approaches is now feasible in biomedical research (29), including applications to veterinary species (30–34). Although most veterinary researchers are not familiar with Bayes theorem, it should arguably be intuitive to practising veterinarians because the method of Bayesian updating (sequentially updating initial beliefs as new evidence becomes available) is analogous to the process by which veterinarians investigate and make diagnoses in their patients (35). With this background in mind, the aim of the current study was to apply causal inference within a Bayesian workflow to investigate associations between body fat mass and other variables on HCC in dogs with obesity.

## Methods

### Animals

All participating animals were referred to a *specialist obesity care clinic* for dogs and cats (Royal Canin Weight Management Clinic, University of Liverpool, Neston, UK) for investigation and management of obesity or obesity-related disorders between May 2018 and February 2023, with all successful weight reduction interventions completed by September 2023. To be eligible, animals had to have had at least one adequate sample of hair available for cortisol analysis, and had to have reached an end point for their therapeutic weight reduction protocol, as described for similar studies in dogs (36); in this respect, some dogs completed their protocol and reached target weight, whilst others stopped prematurely, with the reasons recorded. Dogs diagnosed with either hypothyroidism or hyperadrenocorticism were not eligible given potential effects of these diseases on hair growth and HCC (2). However, having other comorbidities was not a reason for exclusion, and dogs were also not excluded because of the treatment they had received.

### Collection of hair samples and measurement of hair cortisol

Hair samples from each dog were obtained by clipping two sites (jugular groove, antebrachium), whilst preparing for jugular venepuncture and venous catheterisation, respectively. Samples were collected before undertaking any clinical procedures including sedation for dual-energy X-ray absorptiometry (DXA). The quantity of hair collected depended on coat density, although this was always sufficient for HCC measurement. Hair from each site was combined, placed in an individual sealable plastic bag (Ziploc; S. C. Johnson, Wisconsin, USA) and then stored at −20 °C under dark conditions. All samples were subsequently shipped to a commercial laboratory (Dresden Lab Service, Dresden, Germany), with HCC measured in powdered samples by liquid chromatography mass spectrometry. This method has previously been used to measure HCC in dogs (37); the reported quantification limit was 0.09 pg/mg, whilst reported inter- and intra-day coefficients of variation were <10% (38).

### Measurement of body weight and body fat percentage

Body weight was measured by electronic weigh scales, which were regularly calibrated using test weights (2–50 kg; guaranteed to be accurate to within ≤0.5%; Blake and Boughton Ltd., Thetford, UK). In most dogs (26/31 dogs providing single samples; 17/21 [before weight reduction] and 12/21 [after weight reduction] dogs providing paired samples), body fat percentage was analysed using fan-beam DXA (Lunar Prodigy Advance; GE Lunar; Madison, USA), calibrated on a weekly basis using a phantom supplied by the company, in conjunction with a bespoke computer software package (Encore 2004, 8.70.005; GE Lunar) (39). Dogs were either sedated (if DXA alone was performed) or anaesthetised if required for additional procedures, and scanned in dorsal recumbency, as described in a previous study (39).

### Therapeutic weight reduction protocol

Full details of the weight reduction protocol used have been published in previous research (36, 40), although all dogs followed a partial weight reduction plan, meaning that the target weight set was deliberately greater than their ideal weight range, as described in a previous study (41). Briefly, at the first visit, patients were weighed, their body condition score (BCS) recorded and, in most dogs, body composition was also measured by DXA (see above). Health status was determined by routine haematology, serum biochemistry, free thyroxine measurement and urinalysis. If necessary, additional diagnostic investigations (e.g., diagnostic imaging, additional laboratory investigations) were performed to determine the status of any comorbidities. A tailored therapeutic weight reduction protocol was then formulated for each animal, again as described in a previous study (36, 40, 41). Briefly, animals were fed high protein, high fibre dry or moist therapeutic diets (Table 1; Royal Canin, Aimargues, France), with some dogs consuming the dry diet exclusively, and the remainder being fed a combination of wet and dry food, the choice of which depended on owner and animal preferences. The methods used to calculate initial food allocation have again been described in a previous study (41). In addition to advice about feeding the therapeutic diet, owners also received tailored advice on lifestyle alterations to assist the weight reduction process. This included a physical activity plan, tailored to owner circumstances, individual animal factors and the presence of comorbidities. Advice could include recommendations about play activity, walking, running, agility training and hydrotherapy.

**Table 1:** Average composition of the therapeutic diets used for weight reduction in 298 dogs with obesity.

Criterion	HPHF dry ^1^		HPHF wet ^2^	
ME content	2,900 kcal per 1,000 g		602 kcal per 1,000 g	
	As fed ^3^	Per 1,000 kcal	As fed ^3^	Per 1,000 kcal
Moisture	10	33	83	1,379
Crude protein	30	105	8.5	141
Crude fat	10	33	2.0	33
Crude fibre	17	58	2.0	53
Total dietary fibre	28	97	3.2	33
Ash	6	20	1.5	25

^1^Satiety Weight Management Dry (Royal Canin;) 2 Satiety Weight Management Wet (Royal Canin); 3 expressed as grams per 100 g; DM: dry matter; ME: metabolisable energy content, calculated using a predictive equation based on total dietary fibre (TDF).

After the initial visit (V0, before therapeutic weight reduction), animals were reassessed every 7 to 21 days to have their body weight measurements taken, and changes were made to the dietary and exercise plan if necessary. In dogs that reached their target weight, a final evaluation was conducted (V1) after therapeutic weight reduction (median follow-up 313 days; range 116 to 1,609 days). Health status was determined based on physical examination, haematology, serum biochemical analysis and urinalysis. Body weight and body condition were recorded, and body composition was reassessed by DXA.

### Determining sample size

We included as many dogs as possible from those seen during the collection period, with final numbers being equivalent to, or exceeding those used in many previous veterinary studies where HCC was measured (42–45). Arguably, a formal sample size calculation is less critical when using Bayesian methods (46); such analyses automatically account for any uncertainty arising from the sample size because the entire posterior density distribution is reported, with wider intervals reflecting greater uncertainty.

### Causal model development

A variety of factors may induce HPA upregulation and, therefore, increased HCC, in pet dogs with obesity. These relationships were illustrated by constructing a directed acyclic graph (DAG), a graphical tool that provides a bridge between substantive theory (in the form of a scientific model) and statistical analysis (47). Such graphs can identify potential sources of bias enabling statisticians to decide which additional variables should be selected for adjustment in a particular statistical analysis (48, 49). The DAG developed in this study was created using online software [DAGitty software, version 3.1 (50)], and its design was informed by relevant canine and comparative literature. With this software, causal pathways (that directly or indirectly connect the causal variable to the outcome variable), confounding variables and confounding (a.k.a. ‘backdoor’) pathways within a DAG (51) can readily be identified. To obtain an accurate estimate of a causal association, all backdoor pathways must be closed, whilst not closing causal pathways; this is done by ‘conditioning on’ (i.e., including in the model) a set of a variables that are not descendants of the causal variable of interest; doing this should block all backdoor paths, thereby fulfilling the so-called ‘back door criterion’ (51).

**Figure 1:**
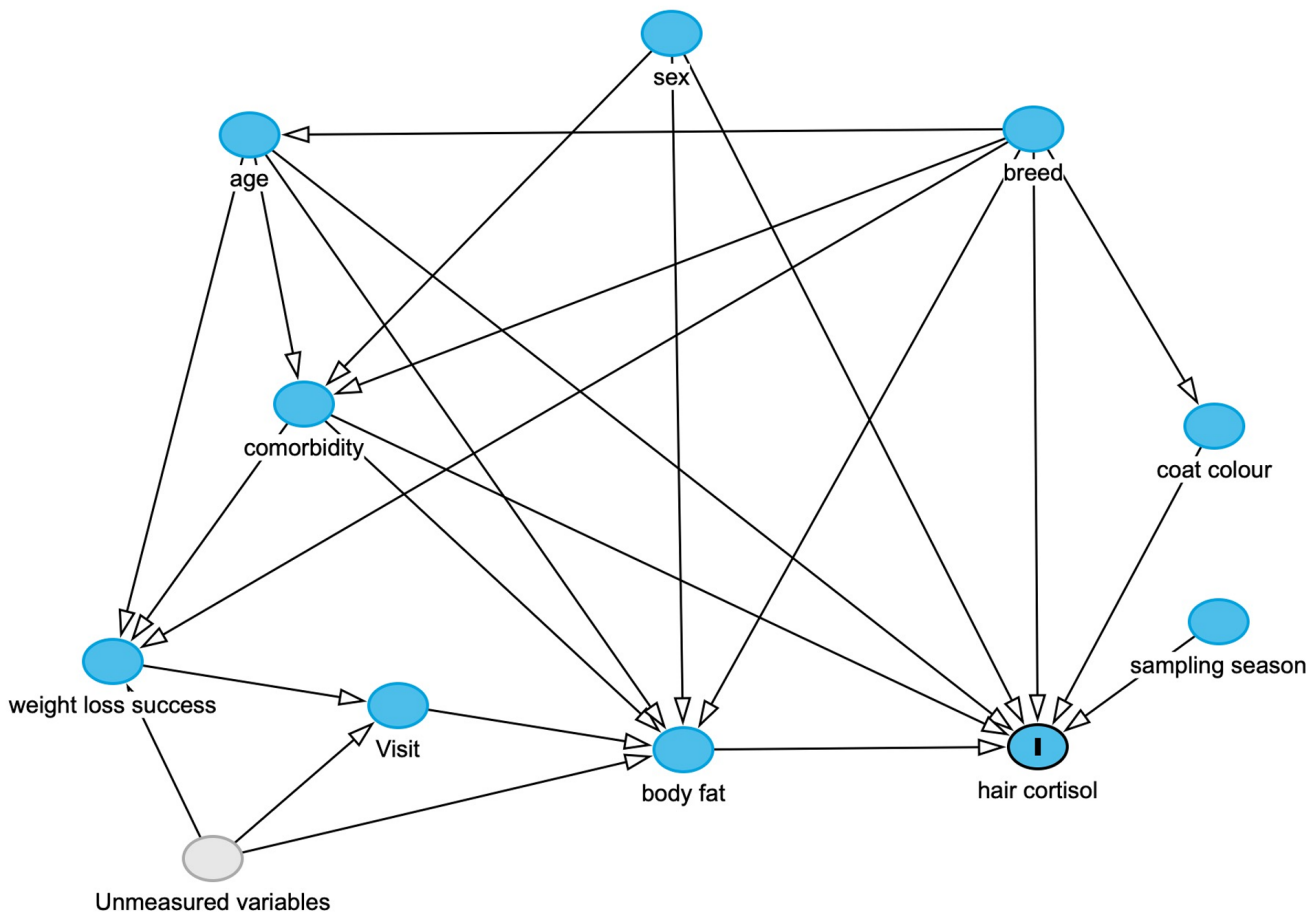
Directed-acyclic graph (DAG), created using the online resource: https://www.dagitty.net, and based on the scientific model which was used to inform the statistical analyses. The outcome variable (marked “I”) is hair cortisol, whilst other variables in the model are either observed (blue circles) or unobserved variables (grey), whilst arrows indicate a causal association between one variable and another. This DAG was used to determine adjustment sets for each of the final models, as shown in the Supplementary File 1. Possible unmeasured variables influencing the ‘weight loss success’, ‘visit’ and ‘body fat variables could include owner factors (e.g., owner attitudes and behaviours in implementing a therapeutic weight reduction protocol), environmental factors (e.g., the living environment of the dog) and possible impacts from the COVID-19 pandemic.

The final DAG is shown in Figure 1, whilst graphical representations of all adjustment sets are provided in the Supplementary File 1. As can be seen, this approach enabled appropriate sets of adjustment variables to be identified for modelling causal associations for most predictor variables, e.g., age, sex, breed, coat colour, body fat, season of sampling and comorbidity. However, appropriate adjustment sets could not be identified for both the ‘successful weight loss’ (i.e., comparing dogs completing a period of therapeutic reduction with those not completing) and ‘visit’ (before vs. after therapeutic weight reduction) variables. This was because, in the causal model, there were backdoor pathways that could not be closed on account of unmeasured confounding (‘backdoor criterion’ not fulfilled). These unmeasured variables included owner or environmental factors affecting feeding and lifestyle behaviours that might plausibly affect body fat mass, weight loss success and attendance at follow-up visits (Figure 1; Supplementary File 1).

### Data handling and statistical analysis

#### Statistical modelling strategy

A Bayesian workflow was chosen for statistical analysis which, as far as possible, has been conducted and reported in compliance with the Bayesian analysis reporting guidelines (52). There were several reasons for selecting such an approach. First, Bayesian methods are particularly well suited to the type of model used (multi-level linear models), and the flexibility they allow in specifying models that are appropriate for the data (52). Second, they perform well when causal associations are being estimated, involving statistical analyses informed by a pre-specified scientific model based on a DAG (49). Third, Bayesian analyses are computationally robust and are better able to handle uncertainty when making predictions in the face of small sample sizes (49).

A fourth advantage of a Bayesian approach is the requirement that all assumptions, both scientific and statistical, be clearly and openly stated in advance, ensuring that a scientist ‘shows their working’. Scientific assumptions include the components of the scientific model, as detailed in a DAG, outlining a scientist’s understanding of the data-generating process (see *causal model development* section). Statistical assumptions are those made when selecting the type of statistical model, deciding on an appropriate likelihood function and in choosing appropriate priors (see the *Variables, likelihood function and model parameters* and *Selection of prior distributions* sections below). Not only must such assumptions be stated and justified in advance, but they should also be considered when interpreting results, thereby reinforcing the notion that “posterior inferences are only as good as the model and experiment that produced the data” (26). A fifth advantage is the use of hypothesis testing in Bayesian inference; rather than being limited to null hypothesis significance testing (e.g., determining evidence against a null hypothesis) as with frequentist statistical methods, probability estimates directly in support of a particular hypothesis can be made. Such hypotheses are more flexible and more intuitive to readers. Finally, as emphasised in the results section, for a Bayesian analysis, it is the entire probability distribution that matters, not simply a point estimate (e.g., mean or median) or whether an arbitrary threshold for statistical significance (e.g., *p* < 0.05) was reached. Therefore, interpretations can be more nuanced, better reflecting the uncertainties of the scientific process and any study findings.

#### Dataset, variables assessed and missing data

The dataset on which all statistical analyses were conducted is provided in the online Supplementary File 2. Continuous baseline data are summarised as either mean [standard deviation (SD)] or median and range, whilst categorical data are reported as a number (percentage). Baseline variables recorded were age (in years), breed group [mixed breed (reference category), cavalier king Charles spaniel (CKCS), pug, retriever, other], coat colour [light (reference category), mixed, dark], sex [female (reference interval), male], comorbidities [no (reference category), yes], body fat percentage and season of sampling [spring (reference category), summer autumn, winter]. As discussed above, body fat percentage data were unavailable from 18 visits, including 9 initial visits (before weight reduction; 5 from dogs providing single samples; 4 from dogs providing paired samples) and 9 visits (after weight reduction; all from dogs providing paired samples). These data were considered ‘missing completely at random’ because the reason they were missing had nothing to do with observed and unobserved data (53). Otherwise, there were no missing data for any other variable assessed (Supplementary File 2).

#### Statistical software

Statistical analysis was performed using an online open-access statistical language and environment (R, version 4.4.3) (28). The additional packages used for data wrangling and visualisation were: ‘dlookr’ [version 0.6.3; (54)], ‘dplyr’ [version 1.1.4 (55)], ‘ggplot2’ [version 3.5.2; (56)], ‘psych’ [version 2.5.3; (57)], ‘reshape’ [version 0.8.9; (58)], ‘readxl’ [version 1.4.5; (59)], ‘tidyverse’ [version 2.0.0; (60)], ‘vioplot’ [version 0.5.1; (61)] and ‘vtable’ [version 1.4.8; (62)]. The additional packages used specifically for Bayesian modelling were: ‘brms’ [version 2.22.0; (62, 63)], ‘bayesplot’ [version 1.12.0; (63, 64)], ‘bayestestR’ [version 0.16.0; (65)], ‘loo’ [version 2.8.0; (66)], ‘priorsense’ [version 2.8.0; (67)], ‘rethinking’ [version 2.42; (68)] and ‘rstantools’ [version 2.4.0; (69)].

#### Variables, likelihood function and model parameters

The primary outcome variable was HCC, which was logarithmically transformed (logHCC) and standardised prior to analysis. Standardisation involves first centering the data (subtracting the mean) and then dividing by the SD, a process which was also applied to all continuous predictor variables. Such standardisation has several advantages; it ensures that the intercept corresponds to the mean value (set to 0), and also makes estimates of beta coefficients (a coefficient that quantifies how much the outcome variable changes for single-unit change in the predictor variable) easier to understand; by standardising, each beta coefficient then reflects the change in outcome variable for a 1-SD change in the predictor variable. As well as improving efficiency of the MCMC algorithm used in computation, it makes prior probabilities easier to set since positive and negative values represent positive and negative effects, respectively.

Separate models were constructed with logHCC as the outcome variable and the following causal predictor variables: age, sex, breed group, coat colour, season of sampling, presence of a comorbidity and body fat. The possibility of reverse causality, in the association between body fat and log HCC, was tested in a separate model; for this model, body fat as the outcome variable and log HCC as the causal predictor variable. For each model, adjustment variables were included as determined from the DAG (Figure 1; Table 2; Supplementary File 1).

**Table 2:** Prior and likelihood specifications of all causal models used in analysis, along with the sets of adjustment variables included, as determined from the directional acyclic graph.

Model	Outcome variable	General parameters		Likelihood function ^3^	Causal predictor		Adjustment set	
		Parameter ^1^	Prior ^2^		Variable ^4^	Prior ^2^	Variable ^4^	Prior ^2^
Age	LogHCC	Intercept Sigma Alpha Group	Normal (𝜇 0, 𝜎 0.5) Exponential (𝜆 1) Normal (𝜇 4, 𝜎 2) Normal (𝜇 0, 𝜎 1)	Skew-normal	Standardised age	Normal (𝜇 0, 𝜎 0.5)	Breed	Normal (𝜇 0, 𝜎 1)
Sex	LogHCC	Intercept Sigma Alpha Group	Normal (𝜇 0, 𝜎 0.5) Exponential (𝜆 1) Normal (𝜇 4, 𝜎 2) Normal (𝜇 0, 𝜎 1)	Skew-normal	Female (ref) Male	Normal (𝜇 0, 𝜎 1)	---	---
Breed group	LogHCC	Intercept Sigma Alpha Group	Normal (𝜇 0, 𝜎 0.5) Exponential (𝜆 1) Normal (𝜇 4, 𝜎 2) Normal (𝜇 0, 𝜎 1)	Skew-normal	Mixed breed (ref) CKCS Pug Retriever Other	--- Normal (𝜇 -0.070, 𝜎 1.5) Normal (𝜇 -0.075, 𝜎 1.5) Normal (𝜇 -0.070, 𝜎 1.5) Normal (𝜇 -0.075, 𝜎 1.5)	---	---
Coat colour	LogHCC	Intercept Sigma Alpha Group	Normal (0, 𝜎 0.5) Exponential (𝜆 1) Normal (𝜇 4, 𝜎 2) Normal (𝜇 0, 𝜎 1)	Skew-normal	Light (ref) Mixed Dark	--- Normal (𝜇 -0.070, 𝜎 1) Normal (𝜇 -0.075, 𝜎 1)	Breed	Normal (𝜇 0, 𝜎 1)
Season of sampling	LogHCC	Intercept Sigma Alpha Group	Normal (𝜇 0, 𝜎 0.5) Exponential (𝜆 1) Normal (𝜇 4, 𝜎 2) Normal (𝜇 0, 𝜎 1)	Skew-normal	Spring (ref) Summer Autumn Winter	Normal (𝜇 0, 𝜎 1)	---	---
Comorbidity	LogHCC	Intercept Sigma ^5^ Alpha Group	Normal (𝜇 0, 𝜎 0.5) --- Normal (𝜇 4, 𝜎 2) Normal (𝜇 0, 𝜎 1)	Skew-normal	No (ref) Yes	Normal (𝜇 0.25, 𝜎 1)	Standardised age Breed Sex	Normal (𝜇 0, 𝜎 0.5) Normal (𝜇 0, 𝜎 1) Normal (𝜇 0, 𝜎 1)
Body fat	LogHCC	Intercept Sigma ^5^ Alpha Group	Normal (𝜇 0, 𝜎 0.5) --- Normal (𝜇 4, 𝜎 2) Normal (𝜇 0, 𝜎 1)	Skew-normal	Standardised body fat	Normal (𝜇 0, 𝜎 0.5)	Standardised age Breed Sex Comorbidity	Normal (𝜇 0, 𝜎 0.5) Normal (𝜇 0, 𝜎 1) Normal (𝜇 0, 𝜎 1) Normal (𝜇 0.25, 𝜎 1)
Reverse causality	Standardised body fat	Intercept Sigma ^5^ Group	Normal (𝜇 0, 𝜎 1) Exponential (𝜆 1) Normal (𝜇 0, 𝜎 1)	Normal	LogHCC	Normal (𝜇 0, 𝜎 0.5)	Standardised age Breed Sex Comorbidity	Normal (𝜇 0, 𝜎 1) Normal (𝜇 0, 𝜎 2) Normal (𝜇 0, 𝜎 1) Normal (𝜇 0, 𝜎 1)

Analyses involving standardised log hair cortisol (LogHCC) as the outcome variable used a skew-normal likelihood function. In the reverse causality model, standardised body fat was the outcome variable, and a normal likelihood function was used. All models included visit as a grouping variable, along with the causal predictor and its respective adjustment sets, as indicated in the table. All continuous variables (log hair cortisol, body fat and age) were standardised prior to analyses, by subtracting the mean and dividing by the standard deviation. This approach improves computational efficiency of MCMC, whilst making it easier to set prior probabilities because positive and negative values represent positive and negative effects, respectively. ^1^The parameters estimated in the model, including intercept, sigma (the standard deviation of the intercept), alpha (skew-normal models; a shape parameter, with positive and negative values indicating right or left skews, respectively) and group (accounting for differences in the intercept and standard deviation amongst individual dogs with repeat measurements). ^2^The priors set for each of the parameters in the model; for each, prior, the selected probability distribution is indicated along with values chosen for the distribution parameters; for normal distributions, two parameters were required: mean (𝜇) and standard deviation (𝜎); for exponential distributions, a single parameter, 𝜆, was required (representing the rate of decline of the exponential curve, with larger values indicating more rapid decline). ^3^The likelihood function selected for the models. ^4^The variables included in the model in question, comprising the predictor variable (the variable for which the causal effect was being calculated) and adjustment set variables included in the model to adjust for confounding, based on the directed acyclic graph (Figure 1; Supplementary File 1). ^5^Note that, for both the body fat model and the comorbidity model, a prior for sigma was not included, because it was separately modelled with a single predictor variable (comorbidity); this enabled the variance of the model to differ across levels of comorbidity.

The statistical analyses used were multi-level Bayesian models, which included dog as a grouping variable (to account for multiple samples from some dogs). Such Bayesian models have the advantage of allowing greater flexibility in the desired model structure. Exact details of the variables and parameters used for each multi-level model are shown in Table 2, whilst full details of the statistical workflow (including code used, statistical outputs and graphs) are available online: https://github.com/AliG71/hair_cortisol.

**Figure 2:**
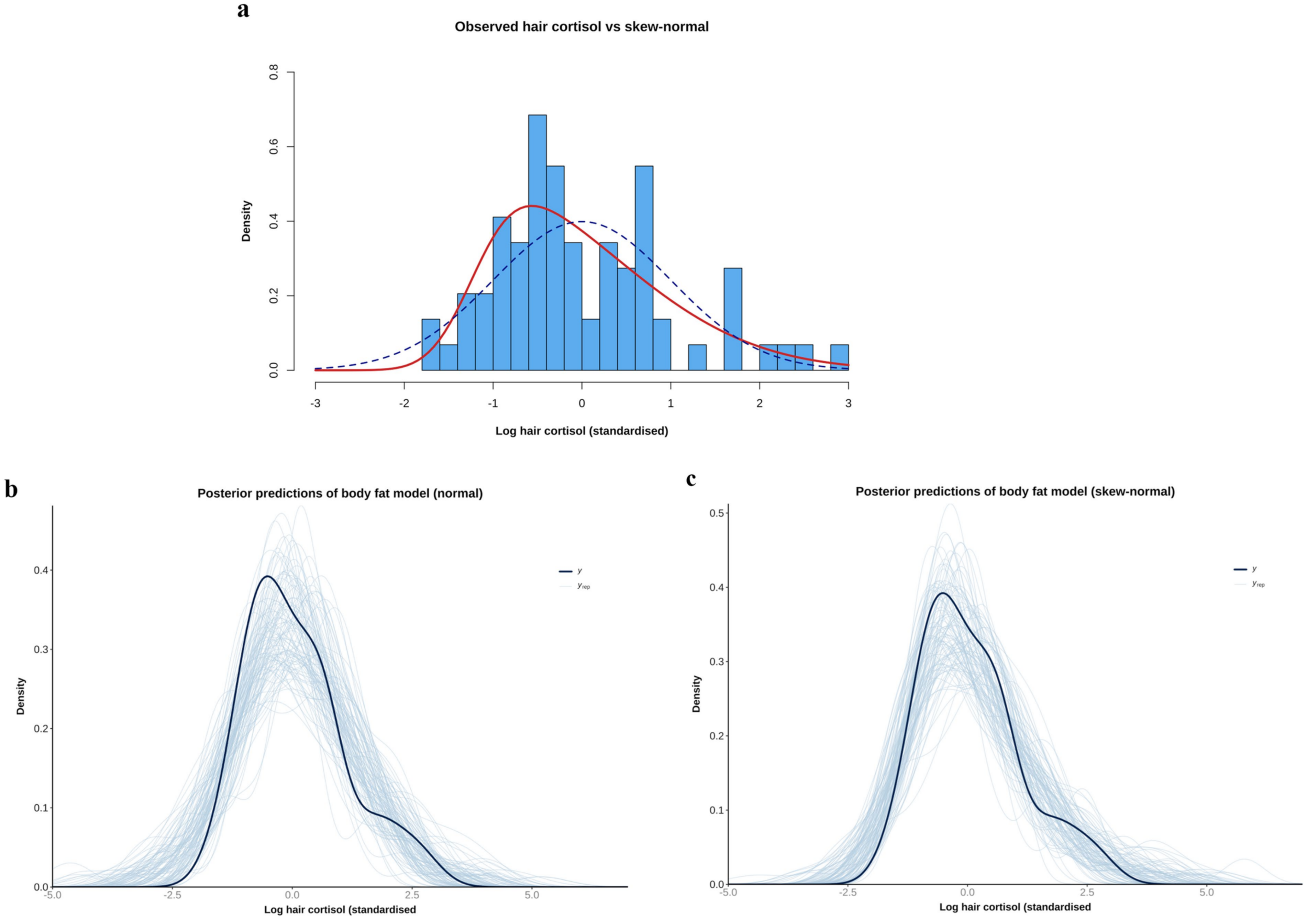
**(a)** Comparison of observed data [log hair cortisol concentration (log HCC)] versus simulations of normal [blue dotted line; mean 0, standard deviation (SD) 1], and skew-normal (red line, mean 0, SD 1, alpha 4) distributions. Note that the observed data have been standardised by subtracting the mean and dividing by the SD. The simulation of the skew-normal distribution better reflects the shape of the observed data. **(b,c)** Comparison of the empirical distribution of the observed outcome variable (logHCCC; thick dark blue curve) with the distributions of many replicated data sets (thin light blue curves) drawn from the posterior predictive distributions of body fat models that use either a normal **(b)** or skew-normal **(c)** likelihood function. Although the model with the normal likelihood function does a reasonable job of predicting the shape of the observed data, the fit is better when a skew-normal likelihood function is used. Specifically, the predictions of the left tail are better aligned (closer to parallel), with means of the predictions clustering around the mean of observed data, and the right-skew is better captured.

Even after logarithmic transformation, data for the outcome variable (logHCC) remained modestly right-skewed. To determine the most appropriate likelihood function, preliminary models with different likelihood distributions (e.g., normal, Student’s *t* and skew-normal) were compared by leave-one-out (LOO) cross-validation using the ‘loo’ package (66). For each model, the leave-one-out information criterion (LOOIC) was calculated, which estimates how well a model can predict future data from the same distribution as the observed data, with smaller values indicating better predictive performance (66). A skew-normal distribution was ultimately chosen given its superior performance, as well as better ability to replicate the distribution of the logHCC data (Figure 2). This distribution is a generalisation of the normal distribution (with mu [mean] and sigma [standard deviation] parameters) but includes an additional ‘shape’ parameter (alpha) to allow for asymmetry, whereby positive and negative values indicate positive and negative skewness, respectively (70). Like the Student’s *t* distribution, it can be more robust to outliers, not least where there is asymmetry around the mean. All final models fitted the data distribution well, as shown by the posterior prediction checks outlined below (Supplementary File 3).

#### Selection of prior distributions

**Figure 3:**
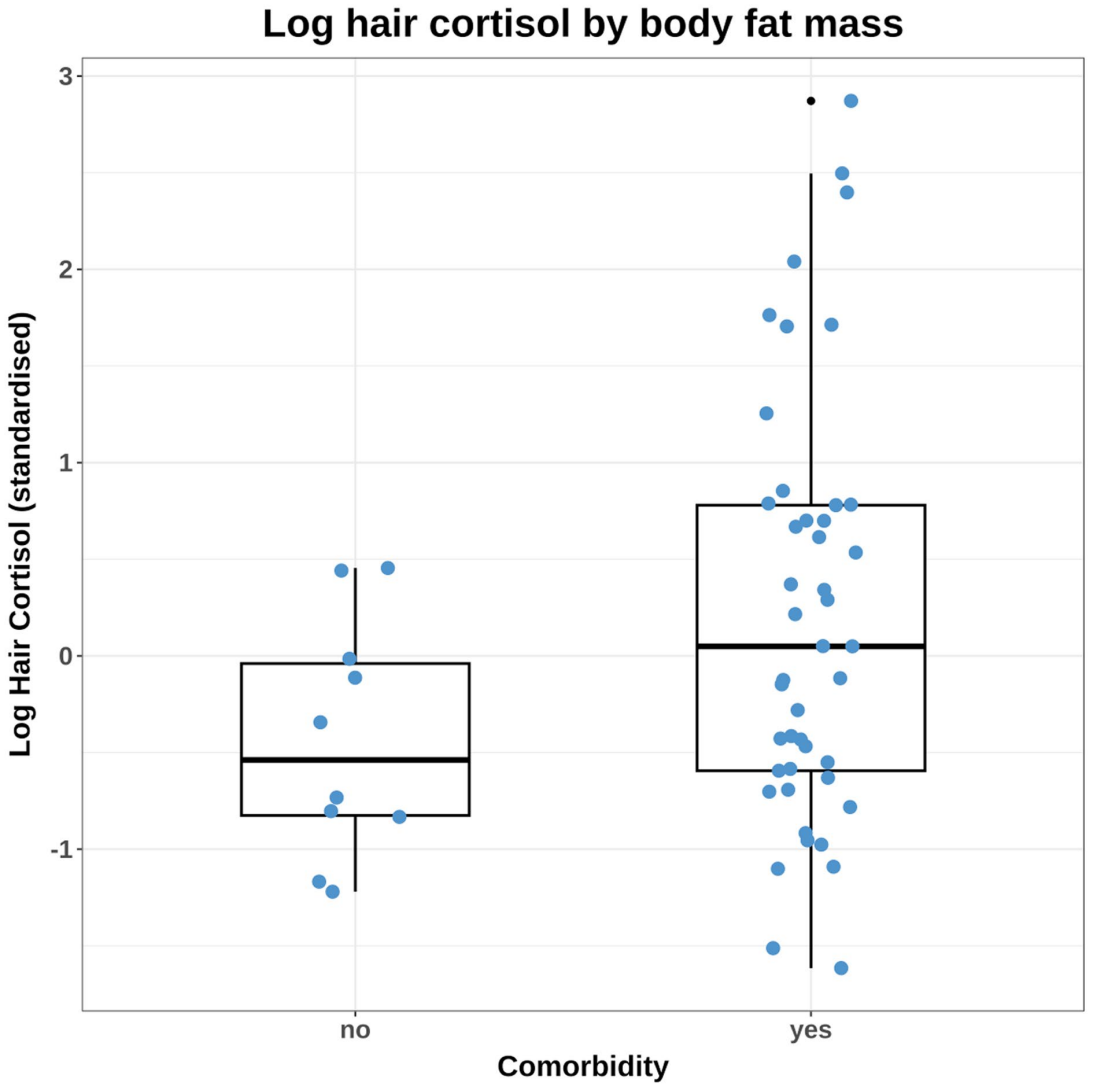
Combined box-and-whisker and dot plot of the observed log hair cortisol concentration (logHCC) data, stratified by the comorbidity variable. The logHCC data have been standardised by subtracting the mean and dividing by the standard deviation. The thick horizontal line represents the median, whilst the upper and lower limits of the box represent the inter-quartile range (IQR). The upper and lower whiskers extend as far as the largest or smallest, respectively, values that are no further than 1.5 × IQR from the IQR, whilst outlying points are shown as small black dots. Given the difference in variance between groups, it was necessary to build some models that accounted for unequal variance amongst dogs that differed by comorbidity status. Affected models included those testing causal associations between comorbidity and HCC and also body fat and HCC (since comorbidity was included in the adjustment set).

**Figure 4:**
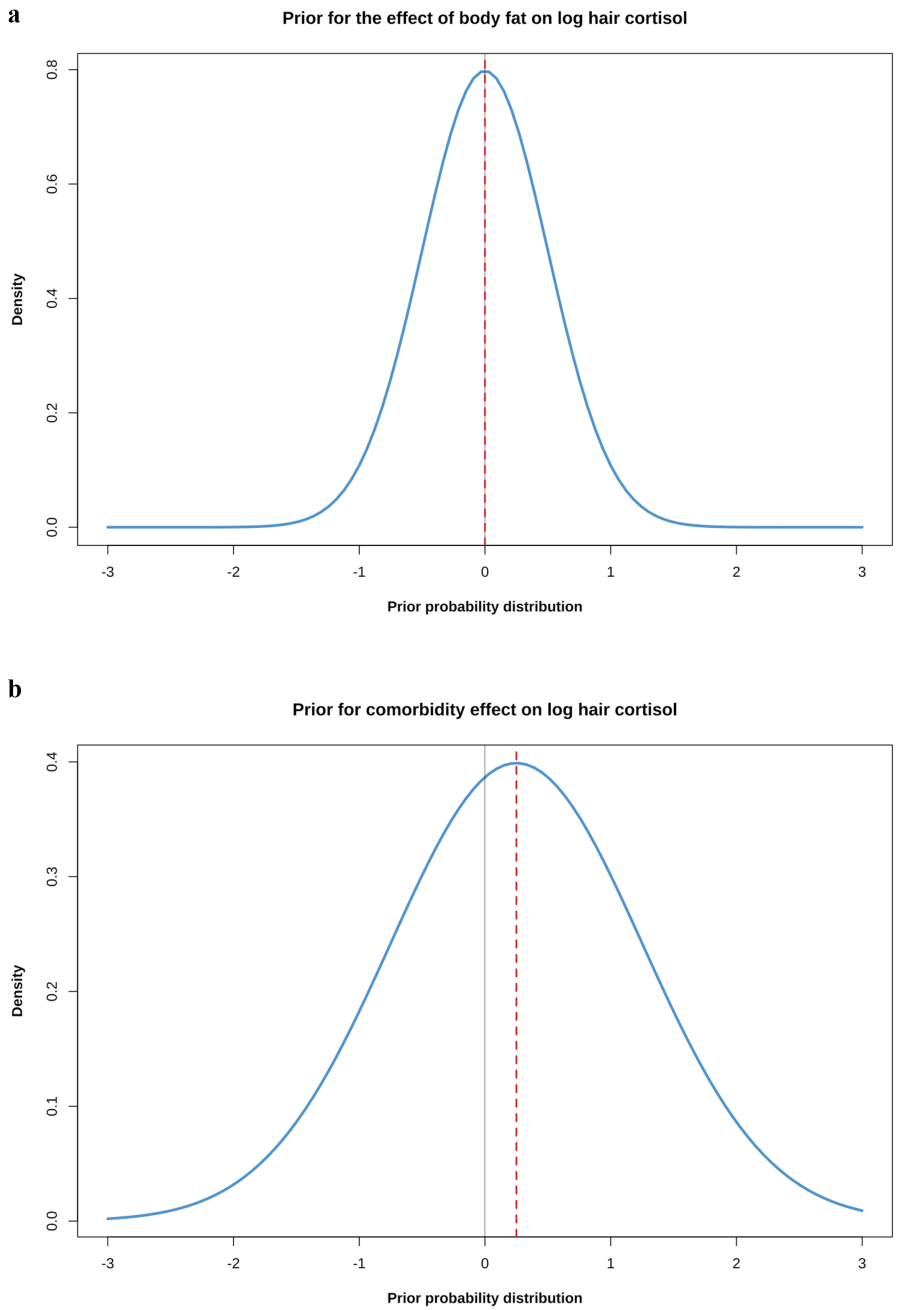
**(a)** Simulated prior probability density distributions (blue lines) for the beta coefficients for causal associations between body fat **(a)** or comorbidity **(b)** and log hair cortisol concentration (logHCC). The thick, red dotted line represents the mean of the density distribution, whilst the solid, thin grey line is intersects the x-axis at zero, indicating a value where the beta coefficient would be neither positive of negative. The body fat prior was assumed to be a normal distribution with a mean of 0 and a standard deviation (SD) of 0.5; the comorbidity prior was also assumed to be a normal distribution, but with a mean of 0.25 and a SD of 1. The impact that such prior probability distributions can have in regularising models is illustrated in Figure 8.

Prior distributions were selected for all parameters of each model, with the overall aim being to ensure they were weakly regularising, adjusted to ensure that that pre-data predictions would span the range of scientifically plausible outcomes. This was confirmed by graphical visualisations and prior predictive simulations from models that sampled from the prior probability distributions only (see below). Justification for the choice of each prior is provided in the Supplementary File 4, whilst details of the final prior distribution choices for each model are shown in Table 2. In most cases, neutral priors were chosen (including for body fat percentage), except for comorbidity and coat colour because previous scientific evidence suggested ‘informed priors’ to be more appropriate (Supplementary File 4). Further, a difference in the variance of HCC between dogs with and without comorbidities was evident (Figure 3) and, therefore, a prior for sigma (standard deviation for the likelihood function) was not included in models that included comorbidity, either as the predictor or an adjustment variable. Instead, sigma was estimated as a parameter within the model, using comorbidity as a single predictor variable (Table 2). Simulated prior probability distributions for the body fat and comorbidity beta coefficients are shown in Figure 4.

#### Model computations and diagnostic checks

Bayesian analyses were computed using the ‘brms’ package [version 2.22.0; (63)], which fits multilevel Bayesian models using the probabilistic programming language, ‘Stan’ (71), accessed via the ‘rstan’ package (version 2.32.7 (68, 69)). All models employed 4 chains with 8,000 iterations (including 2,000 and 6,000 warm-up and sampling iterations, respectively). Diagnostic checks included checks of MCMC performance and also several prior and posterior validation checks (Supplementary File 3), with full details of all statistical analyses (including the code used and all statistical output) available online: https://github.com/AliG71/hair_cortisol. To verify MCMC performance, models were checked for convergence, by assessing R-hat values and inspecting both trace and trace-rank plots (Supplementary File 3), whilst resolution was assessed by calculating effective sample sizes (Table 4). In all final models, effective sample sizes were always acceptable (typically 10,000–20,000 depending upon the model).

**Table 4:** Summary of causal associations for the final models where log hair cortisol concentration was the outcome variable.

Model	Estimate ^1^	Estimated error ^1^	97% HPDI ^2^	R-hat ^3^	ESS ^4^		Bayes R^2,5^	
					Bulk	Tail	Median	97% CI
Age (per SD change)	−0.12	0.10	−0.33, 0.10	1.00	14,786	15,585	0.05	0.01, 0.14
Sex							0.02	0.00, 0.11
Female	Ref.	---	---	---	---	---		
Male	0.14	0.20	−0.29, 0.60	1.00	16,874	14,950		
Breed group								
Mixed breed	Ref.	---	---	---	---	---	0.04	0.01, 0.13
CKCS	0.14	0.38	−0.72, 0.92	1.00	13,139	14,313		
Pug	0.19	0.36	−0.59, 0.96	1.00	14,815	14,562		
Retriever	0.09	0.28	−0.52, 0.70	1.00	12,104	15,143		
Other	0.10	0.26	−0.46, 0.65	1.00	14,704	14,362		
Coat colour							0.08	0.02, 0.19
Light	Ref.	---	---	---	---	---		
Mix	−0.50	0.29	−1.13, 0.13	1.00	14,208	15,167		
Dark	−0.33	0.23	−0.83, 0.17	1.00	15,303	16,240		
Season of sampling							0.07	0.01, 0.18
Spring	Ref.	---	---	---	---	---		
Summer	−0.18	0.26	−0.73, 0.37	1.00	12,907	12,966		
Autumn	−0.32	0.27	−0.89, 0.29	1.00	12,768	13,794		
Winter	0.26	0.28	−0.34, 0.88	1.00	12,885	13,103		
Comorbidity							0.13	0.04, 0.25
No	Ref.	---	---	---	---	---		
Yes	0.65	0.24	0.11, 1.16	1.00	16,588	15,963		
Body fat (per SD change)	0.33	0.13	0.03, 0.59	1.00	15,056	13,776	0.18	0.08, 0.29

All analyses used Bayesian multi-level modelling, using the variables and parameters specified in Table 2. All models employed 4 chains, parallelised on separate computer cores, and each using 8,000 iterations (including 2,000 and 6,000 warm-up and sampling iterations, respectively; total available iterations 24,000). ^1^Estimate and estimated error of the beta coefficient for the causal effect of each model. ^2^Highest posterior density interval (a.k,a. highest density interval), the narrowest interval containing the specified probability mass (here 97%), and representing the most probable region of the value of the parameter, given the model, the priors and the observed data. ^3^R-hat is a convergence diagnostic, which compares between- and within-chain estimates for model parameter; values larger than 1 suggest that the Markov chains in the model have not mixed well, with a commonly-accepted cut-off for acceptability being 1.05. ^4^Estimated sample sizes from the Markov Chain Monte Carlo simulation; these are estimates of the number of samples that were used and provide a useful measure of sampling efficiency. As the names suggest, the bulk ESS and tail ESS provide estimates for the sampling efficiency in the bulk and tails of the distribution, respectively; values >100 per Markov chain (here >400) indicate that estimates of respective posterior quantiles are reliable. ^5^Median and 97% compatibility interval of the posterior distribution of the Bayesian *R*^2^ estimate; with this approach, the “variance of the predicted values is divided by the variance of predicted values plus the expected variance of the errors,” with the results “quantifying the fit of the model to the data at hand” (69).

Verifications of the suitability of prior probabilities included initial graphical modelling, to simulate the expected shape of the distribution, prior predictive simulations and power scaling sensitivity analysis [*powerscale_sensitivity* function of the ‘priorsense’ package (67)]. Verification checks on the posterior distributions included a visual inspection of a pairs plot, a graphical posterior predictive check, graphical comparisons of individual draws against the observed data for each predictor variable and several other graphical checks (Supplementary File 3).

Finally, before testing on the actual study data, models were checked using simulated data to ensure that model fitting had worked correctly. For this, different simulated datasets of 200 visits [100 each for the first (V0) and second (V1) visits from 100 dogs] were created, each specifying different effect sizes for the causal predictors of each model (e.g., in the body fat model, it was assumed that the beta coefficient for the causal effect could be of 0.0, 0.2 or 0.5). All model estimates for the beta coefficient reliably reproduced what was expected in the simulated dataset (https://github.com/AliG71/hair_cortisol).

#### Analyses of the posterior distribution

Posterior density distributions for all parameters in the final models were calculated as described above and summarised using means (for central tendency) and 97% highest posterior density intervals (97% HPDI, for limits of the credible interval), unless otherwise indicated. Graphical visualisation of posterior probability distributions included plots of posterior densities (displaying highest density intervals), plots of conditional effects and plots comparing the prior and posterior probability distributions (to illustrate the relative contribution of the prior and sample data). Hypothesis tests were conducted to determine the probability that the effects of each causal predictor were positive (or negative), and posterior predictions were used (from new simulated data) to estimate causal associations. Finally, the Bayes *R*^2^ metric was calculated using the *bayes_R2* function of the ‘rstantools’ package (69), which is similar to a conventional R^2^ statistic from least-squares linear regression, with the results “quantifying the fit of the model to the data at hand” (69).

#### Sensitivity analyses

Sensitivity analyses included testing the sensitivity of the posterior distribution to the choice of prior distribution, as described above. Other analyses were conducted, on the body fat and comorbidity models, to determine the sensitivity of posterior distribution estimates to measurement error (given known variability in the hair cortisol assay), season of sampling, missing data (body fat model only) and setting a neutral regularising prior for comorbidity (comorbidity model only). Measurement error in the response variable (logHCC) was accommodated using the *mi()* syntax in the ‘brms’ package (63), and assuming that the coefficient of variability of hair cortisol measurement was 11.8% (37). Although not on the causal pathway for either model (Figure 1; Supplementary File 1), a sensitivity analysis was conducted to determine the effect of sample season on causal estimates in the body fat and comorbidity models. This was undertaken by recomputing posterior probability distribution after adding the sampling season variable to each model. To assess a possible effect of missing data in the body fat variable, the *mi()* syntax from the ‘brms’ package (63) was again used; this involved fitting a multivariate Bayesian multilevel model, whereby the missing data for body fat and log hair cortisol are simultaneously predicted (see the sensitivity analyses report in the online material at: https://github.com/AliG71/hair_cortisol). Using this approach, missing data are handled as additional parameters for estimation from a single joint posterior distribution. Knowledge of variables causally-associated with the missing variable can also be used to inform the imputation process. Together, this leads to more accurate and honest credible intervals than other imputation approaches such as single imputation. For the body fat model, a Student’s *t* likelihood function was chosen, and incorporated appropriate predictor variables as indicated by the DAG (Figure 1; Supplementary File 1; e.g., sex, age, breed group and comorbidity). Finally, to determine whether the final choice of prior for comorbidity (marginally-positive, weakly-regularising; mean 0.25, sigma 1) had unduly influenced the posterior probability density, the comorbidity model was rerun with a different prior that was neutral and weakly regularising (mean 0, sigma 1).

### Ethics and welfare considerations

The study has been reported in accordance with the Animal Research: Reporting of *In Vivo* Experiments (ARRIVE) guidelines.^1^ The study received approval from both the University of Liverpool Veterinary Research Ethics Committee (RETH000353 and VREC793) and the Royal Canin Ethical Review Committee (150720–55). All owners gave informed, written consent allowing their dog to participate. Clinical procedures complied with relevant guidelines (e.g., standard operating procedures) and regulations. Foods used were commercially available therapeutic diets commonly used by veterinarians to manage obesity, and given for the clinical benefit of the study dogs. Neither the clinical procedures used nor the clinical use of the therapeutic diets were deemed to involve animal experimentation, falling outside the remit of national legislation (e.g., the revised Animals [Scientific Procedures] Act 1986).

## Results

### Characteristics of dogs, hair samples and HCC results

In total, 73 hair samples were obtained, comprising paired samples from 21 dogs (before [V0] and after [V1] weight reduction), and single samples (before weight reduction only [V0]) from a further 31 dogs. Full details of all baseline characteristics are shown in Table 3. Forty-four of the 52 dogs (85%) had one or more comorbidities (median 1, range 0–3). Twenty-five dogs ate the dry therapeutic food during their period of weight reduction (16 dogs with paired samples; 9 dogs with single samples), whilst the remaining 27 consumed a combination of wet and dry therapeutic food (12 dogs with paired samples; 15 dogs with single samples). In the dogs with paired samples, mean weight loss was 26% (SD 6.8%) of starting weight, at a rate of 0.7% (SD 0.33) per week, with median body fat mass being 42% (range 31–61%) and 33% (range 18–50%) before and after weight reduction, respectively.

**Table 3:** Baseline variables in dogs in the study.

Variable	All dogs	Single hair sample (V0 only)	Paired hair samples (V0 and V1)
Number	52	31	21
Age (months)			
Visit 0	90 (34.0)	90 (33.9)	91 (36.1)
Visit 1	---	---	109 (35.5)
Sex			
Female (neutered)	30 (58%)	17 (55%)	13 (62%)
Male (neutered)	22 (42%)	14 (45%)	8 (38%)
Breed			
Mixed breed	11 (21%)	6 (19%)	5 (24%)
Cavalier King Charles Spaniel	6 (12%)	5 (16%)	1 (5%)
Pug	5 (10%)	3 (10%)	2 (10%)
Retriever	12 (23%)	7 (23%)	5 (24%)
	Labrador retriever 11 Golden retriever 1	Labrador retriever 7 Golden retriever 0	Labrador retriever 4 Golden retriever 1
Other	18 (35%)	10 (32%)	8 (38%)
	Beagle, Bichon Frise 2, Bulldog Cocker Spaniel 2, Corgi, French Bulldog, Lhasa Apso, Miniature Dachshund, Miniature Schnauzer 2, Norfolk Terrier, Polish Lowland Sheepdog, Rhodesian Ridgeback Staffordshire Bull Terrier 3	Beagle, Bichon Frise 2, Bulldog French Bulldog, Mini Schnauzer, Norfolk Terrier, Staffordshire Bull Terrier 3	Cocker Spaniel 2, Corgi, Lhasa Apso, Polish Lowland Sheepdog, Miniature Dachshund, Miniature Schnauzer, Rhodesian Ridgeback
Coat colour			
Light	18 (35%)	8 (26%)	10 (48%)
Mixed	13 (25%)	10 (32%)	3 (14%)
Dark	21 (40%)	13 (42%)	8 (38%)
Season			
Spring	10 (29%)	9 (29%)	1 (5%) / 4 (19%)
Summer	14 (16%)	5 (16%)	9 (43%) / 8 (38%)
Autumn	18 (32%)	10 (32%)	8 (38%) / 3 (14%)
Winter	10 (23%)	7 (23%)	3 (14%) / 6 (29%)
Comorbidities			
No	8 (15%)	1 (3%)	7 (33%)
Yes	44 (85%)	30 (97%)	14 (67%)
Total	59	35	24
Number per dog	1 (0 to 3)	1 (0 to 3)	1 (0 to 3)
	Dental-oral 8 Orthopaedic 17 Cardio-respiratory 12 Dermatological 16 Neoplastic 6	Dental-oral 6 Orthopaedic 10 Cardio-respiratory 7 Dermatological 9 Neoplastic 3	Dental-oral 2 Orthopaedic 7 Cardio-respiratory 5 Dermatological 7 Neoplastic 3
Body fat percentage ^1^			
Before	43 (6.0)	41 (5.5)	45 (6.4)
After	---	---	33 (8.8)
Hair cortisol (pg/mg)			
Before	10.4 (19.52)	11.5 (21.91)	7.6 (14.29)
After	---	---	3.6 (3.74)
Log hair cortisol			
Before	1.3 (1.36)	1.4 (1.33)	1.1 (1.23)
After	---	---	0.9 (0.94)

Except for number of comorbidities (presented as median and range), results are presented either as number (percentage) for count data or mean (standard deviation) for continuous data. ^1^Body fat percentage determined by dual-energy X-ray absorptiometry. V0: the visit before therapeutic weight reduction; V1: the visit after therapeutic weight reduction.

There were various reasons why 31 of the dogs only contributed a single hair sample. Just over half (16 dogs) had reasons related to the COVID-19 pandemic: of these, 3 dogs reached target weight during the pandemic, but a face-to-face follow-up visit was not possible; the remaining 13 pandemic-affected dogs were lost to follow-up, mostly because owners had found it difficult to implement a therapeutic weight reduction protocol. Of the 15 dogs not affected by the COVID-19 pandemic, 3 dogs completed therapeutic reduction but their owners decided not to return for the follow up; 2 dogs were euthanased for other reasons (e.g., metastatic pulmonary adenocarcinoma, old age) before reaching target weight; and the remaining 10 dogs were lost to follow-up (4 due to poor compliance; 4 stopped responding to communications; 1 moved away from the area; 1 had transport issues).

### Hair cortisol concentrations

Details of HCC results are shown in Table 3. The mean HCC for all samples was 10.4 (SD 19.52) pg/mg (logHCC 1.3, SD 1.36). In the dogs providing single samples (before therapeutic weight reduction, V0), mean HCC was 11.5 (SD 21.91) pg/mg (log HCC 1.4, SD 1.33) whereas, in those providing paired samples, HCC was 7.6 (SD 14.29) pg/mg (log HCC 1.1, SD 1.23) before (V0) and 3.6 (SD 3.74) pg/mg (log HCC 0.9 SD 0.94) after (V1) weight reduction.

### Causal associations between hair cortisol and different explanatory variables

The results of estimates of the causal effects from each model are summarised in Table 4 and Supplementary File 5, with full details of each statistical model and validation available online: https://github.com/AliG71/hair_cortisol.

#### Age model

Based on the DAG (Figure 1; Supplementary File 1), the only adjustment variable required for the age model was breed group. Although the probability distribution for the effect of age (beta coefficient) spanned zero (Supplementary File 5), and the majority was negative (mean −0.12 per SD; 97% HPDI -0.33, 0.10). A weakly-negative trend was seen across the age range (Supplementary File 5), equating to an average decrease in HCC of ~1.6 pg/mg (~0.1 logHCC) for each 1-unit (~36 months) increase in standardised age. Therefore, we estimated an 88% probability of there being a negative effect of age on logHCC.

#### Sex model

Based on the DAG (Figure 1; Supplementary File 1), no adjustment variables were required for the sex model. The probability distribution for the effect of sex again spanned zero (Supplementary File 5), but did not clearly favour either a positive or negative predictions (mean 0.14, 97% HPDI -0.29, 0.60). Therefore, we estimated a 75% probability of there being a positive causal association between sex and logHCC.

#### Breed group model

Based on the DAG (Figure 1; Supplementary File 1), no adjustment variables were again required for the breed group model. Compared with mixed breed as the reference category, probability distributions for all other categories spanned zero (Supplementary File 5), and did not suggest any clear positive or negative associations with logHCC (CKCS mean 0.14, 97% HPDI -0.72, 0.92; pug mean 0.19, 97% HPDI -0.59, 0.62; retriever mean 0.09, 97% HPDI -0.52, 0.70; other breed mean 0.10, 97% HPDI -0.46, 0.65). Therefore, the probability of positive causal associations with logHCC were estimated to be 66%, 71%, 63% and 65% for the CKCS, pug, retriever and other breed groups, respectively.

#### Coat colour model

Based on the DAG (Figure 1; Supplementary File 1), the only adjustment variable required for the coat colour model was breed group. Compared with light coat colour as the reference category, the probability distributions for mixed and dark coat colour spanned zero (Supplementary File 5) but were predominantly negative in both instances (mixed: mean −0.50, 97% HPDI -1.13, 0.13; dark: mean −0.33, 97% HPDI -0.83, 0.17). Therefore, we estimated 93% probability and 96% probability that logHCC is less in dogs with dark or mixed coat colour, respectively than in dogs with light coat colour.

#### Season of sampling model

Based on the DAG (Figure 1; Supplementary File 1), no adjustment variables were again required for the sampling season model. Compared with spring as the reference category, all probability distributions spanned zero (Supplementary File 5), although those for summer (mean −0.18; 97% HPDI -0.73, 0.37) and autumn (mean −0.32; 97% HPDI -0.89, 0.29) were majority negative, whilst that for winter was majority positive (mean 0.26; 97% HPDI -0.34, 0.88). Therefore, the probability of negative causal associations between summer and autumn and logHCC were 77% and 88%, respectively, whilst the probability of a positive causal association between winter and log HCC was 83%.

#### Comorbidity model

**Figure 5:**
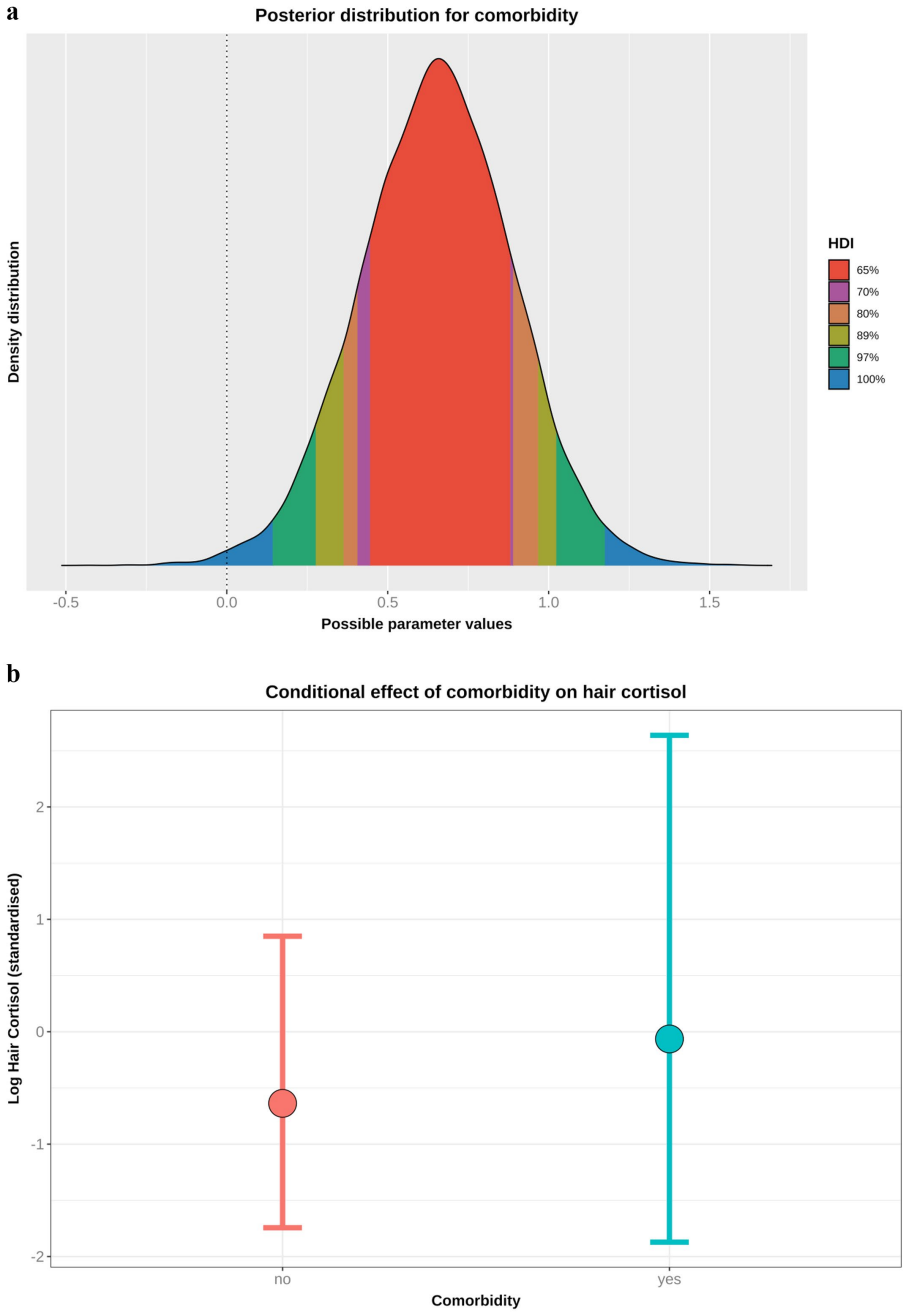
**(a)** Highest probability density interval (a.k.a. highest density interval) plot for the causal association between comorbidity and log hair cortisol concentration (logHCC). The y-axis depicts the density distribution, whilst the x-axis depicts possible values for the beta parameter of the causal association. Different probability intervals are depicted by colour (red 65%; purple 70%; orange 80%; yellow 89%; green 97%; blue 100%). The feint vertical dotted line intersects the x-axis at zero, indicating the point where the beta coefficient would be neither positive of negative. Most of the posterior probability density is greater than zero, indicating a > 99% probability for a positive causal association. **(b)** Conditional effect for the association between comorbidity and logHCC, generated using the *posterior_predict* function of the ‘brms’ package [version 2.22.0; (62)]. The points represent estimates, whilst the lines represent the 97% compatibility interval (97%-CI). This method estimates uncertainty in predictions from both the statistical model and residual error, which better reflects the complete range of plausible outcomes of the scientific model. Mean predicted logHCC is greater in dogs with comorbidities, with a wider uncertainty range, than in dogs without comorbidities.

**Figure 6:**
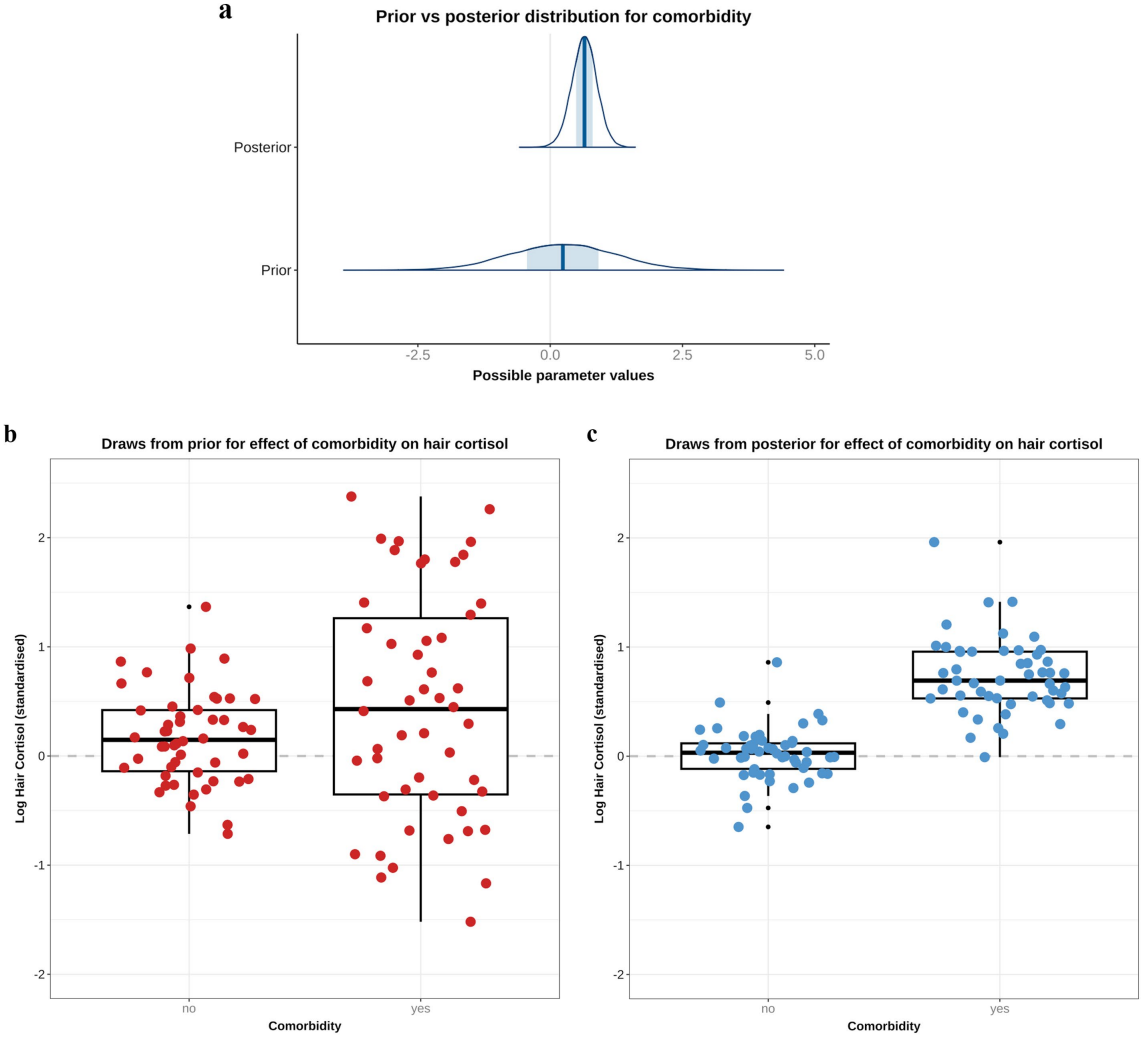
A comparison of prior and posterior distributions for the causal association between comorbidity and log hair cortisol concentrations (logHCC). **(a)** Comparison of prior and posterior probability densities (thin blue lines). Thick vertical lines within these densities represent the mean and the shaded blue regions represent the central (50%) compatibility interval. Differences in in the shape and position of the posterior distributions, relative to the prior distribution, indicate the effect of the observed data on the predictions. **(b,c)** Visual comparison of predicted differences in logHCC between dogs with and without comorbidities, based on individual predictions created using 50 random draws taken from the prior **(b)** or posterior **(c)** probability distribution. Data are displayed as combined box-and-whisker and point plots, with thick horizontal lines representing the median, and upper and lower limits of the boxes represent the inter-quartile range (IQR). The upper and lower whiskers extend as far as the largest or smallest, respectively, values that are no further than 1.5 × IQR from the IQR, whilst outlying points are shown as small black dots. Prior to observing the data, the model predicts a slightly positive average causal association, but with wide degree of uncertainty, as indicated by the wide density distribution spanning zero **(a)**, and only a marginal average difference between the box and whisker plots for each group **(b)**. After seeing the data, the model is confident that the causal association between comorbidity and logHCC is positive, as indicated by the narrower density distribution almost-completely >0, and greater difference between box-and-whisker plots **(C)**.

Based on the DAG (Figure 1; Supplementary File 1), the adjustment set required for the comorbidity model included age, sex and breed group. The entire posterior probability distribution was positive (mean 0.65; 97% HPDI: 0.11, 1.16; Bayes R^2^ 0.13; Figure 5a), meaning that having a comorbidity was associated with an average increase in HCC of 10.4 pg/mg, compared with no comorbidity (Figure 5b). The relative contributions of the prior expectations and the observed data were determined in two ways; first, the prior and posterior density distributions of the beta coefficient for comorbidity were visually compared (Figure 6a); second, a visual comparison was made of 50 random draws taken from the prior (Figure 6b) and posterior (Figure 6c) probability distributions. Prior to observing the data, the model expected the causal association between comorbidity and logHCC to be slightly positive, on average, but with wide degree of uncertainty as indicated by the mean effect of the prior being >0 but with wide variability (Figure 6a) and the fact that individual posterior predictions of logHCC were slightly more likely to be positive than negative, albeit to varying degrees (Figure 6b). After seeing the data, the model was both confident that the causal association would be positive, and relatively confident about its magnitude, as indicated by the narrow range of the posterior distribution (Figure 6a) and the close clustering of the posterior predictions of logHCC (Figure 6c). Therefore, we estimated a > 99% probability of a positive causal association between comorbidity and logHCC.

Sensitivity analyses (https://github.com/AliG71/hair_cortisol) demonstrated that these results were robust to the effects of measurement error (mean 0.65, 97% HPDI 0.11, 1.17; probability >99%; 10.4 pg/mg mean increase in HCC when one or more comorbidity present), sampling season (mean 0.60, 97% HPDI 0.03, 1.16; probability >99; 9.6 pg/mg mean increase in HCC when one or more comorbidity present) and using a neutral, weakly-regularising prior for comorbidity (mean 0.63, 97% HPDI 0.10, 1.14; probability >99%; 10.1 pg/mg mean increase in HCC when one or more comorbidity present).

#### Body fat model

**Figure 7:**
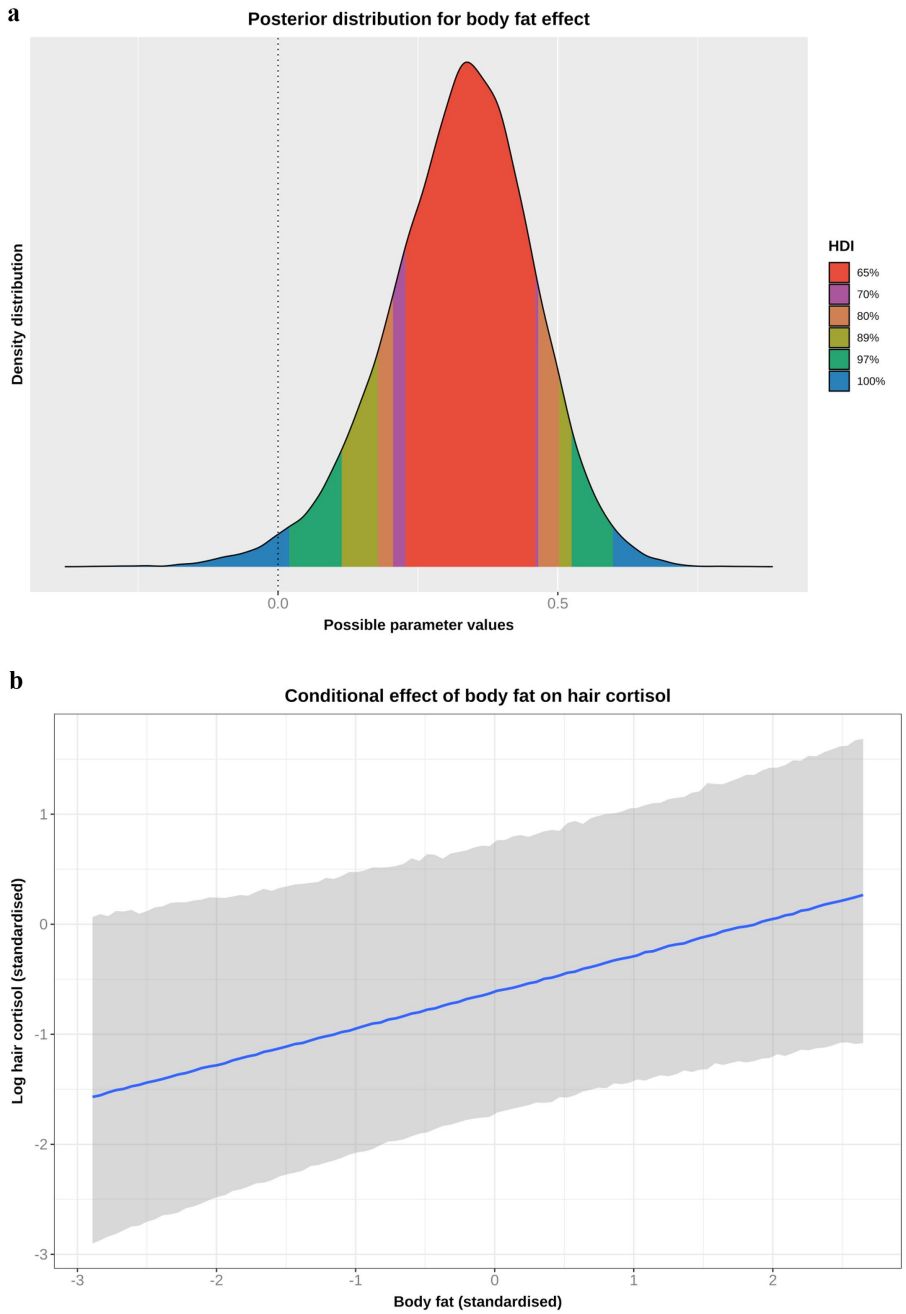
**(a)** Highest probability density interval (a.k.a. highest density interval) plot for the causal association between body fat and log hair cortisol concentration (logHCC). The y-axis depicts the density distribution, whilst the x-axis depicts possible values for the beta parameter of the causal effect. Different probability intervals are depicted by colour (red 65%; purple 70%; orange 80%; yellow 89%; green 97%; blue 100%). The feint vertical dotted line intersects the x-axis at zero, indicating the point where the beta coefficient would be neither positive of negative. Most of the posterior probability density is greater than zero, indicating a 98% probability that the causal association is positive. **(b)** Conditional effect for the causal association between body fat and logHCC. The blue line represents the mean estimate for logHCC, across the body fat range, whilst the shaded region represents the 95% credible interval. Estimates were generated using the *posterior_predict* function of the ‘brms’ package [version 2.22.0 (62)], which returns the posterior mean and 95% credible interval for each data point, thereby incorporating uncertainty in predictions from the posterior distribution (model predictions) and uncertainty due to residual error (from individual data points). Credible intervals generated in this way reflect the complete range of plausible outcomes from the scientific model. Overall, there is a positive linear relationship between body fat and logHCC.

**Figure 8:**
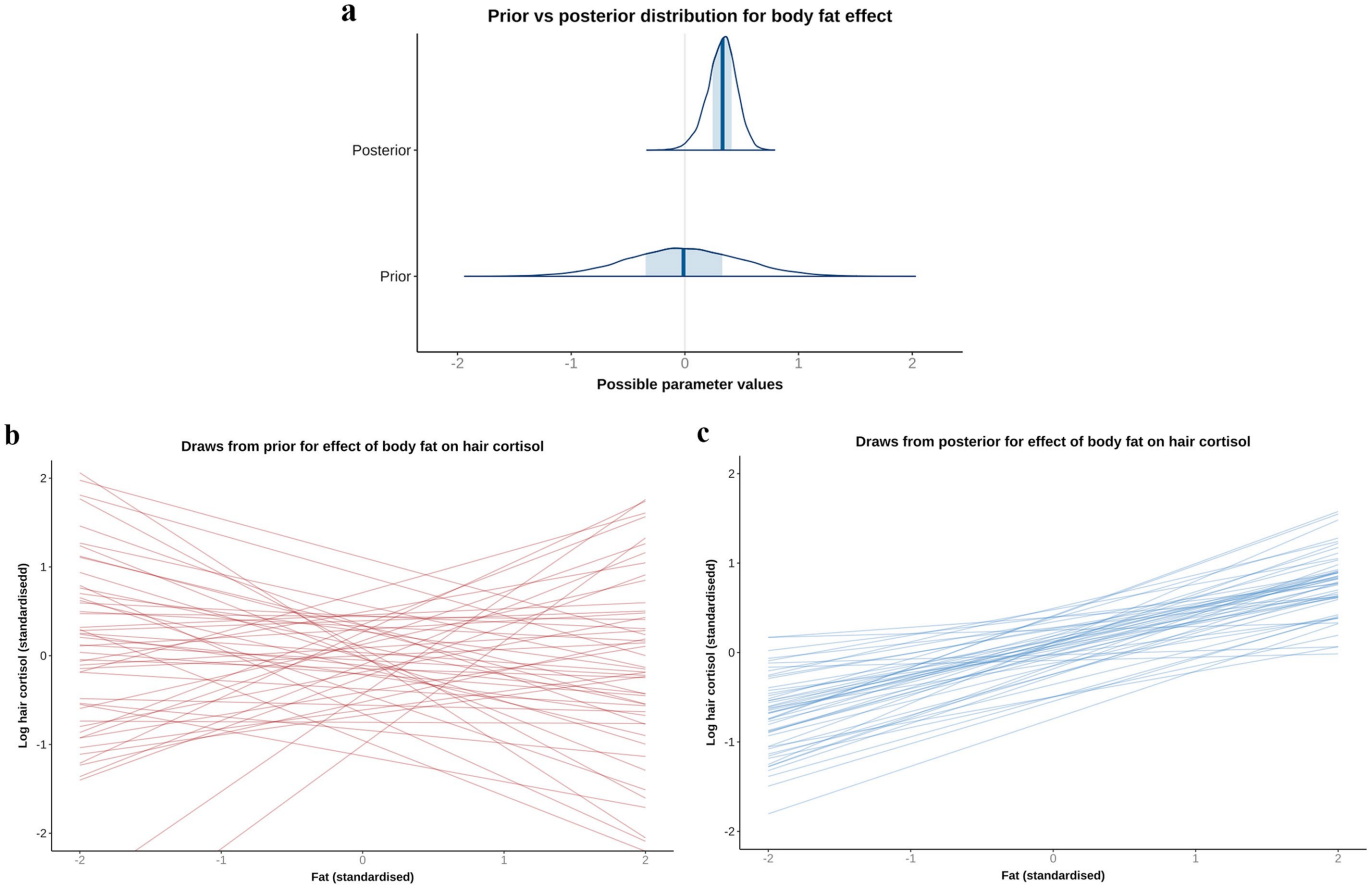
A comparison of prior and posterior distributions for the causal association between body fat and log hair cortisol concentrations (logHCC). **(a)** Comparison of prior and posterior probability densities (thin blue lines). Thick vertical lines within these densities represent the mean and the shaded blue regions represent the central (50%) compatibility interval. Differences in the shape and position of the posterior distributions, relative to the prior distribution, indicate the effect of the observed data on the predictions. **(b,c)** Visual comparison of predicted differences in regression lines. Each line is an individual prediction of the regression slope, calculated from the intercept and beta coefficient for body fat, using one of 50 random draws from the prior **(b)** or posterior **(c)** probability distributions. Prior to observing the data, the model is both neutral and uncertain about the possible causal association between body fat and logHCC, as indicated by the wide density distribution spanning zero **(a)** and wide range of possible regression slopes **(b)**, albeit limited to a physiologically plausible range by the regularising nature of the prior. After seeing the data, the model is more that the association is positive, as indicated by a narrower density distribution that is predominantly above zero, and a more limited range of regression slopes, all of which are positive.

**Figure 9:**
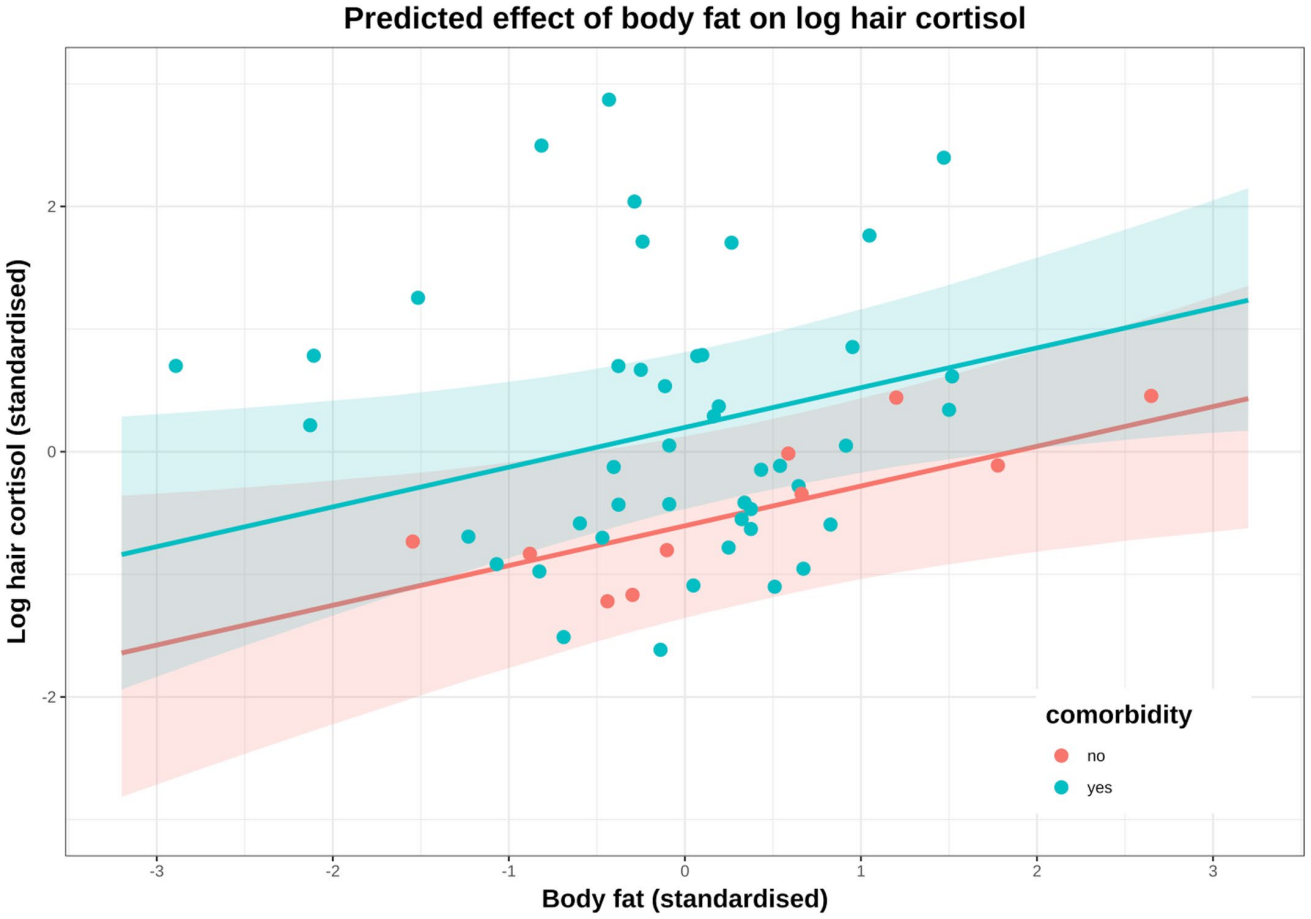
Conditional effect of body fat on log hair cortisol concentration (logHCC), stratified by comorbidity (red: no comorbidity, blue: comorbidity) and compared with the observed data. Solid lines represent mean predictions in logHCC across the body fat range, with shaded areas representing 95% compatibility intervals, and points representing observed results from individual dogs. Estimates were generated using the *posterior_epred* function of the ‘brms’ package [version 2.22.0 (62)], which only incorporates uncertainty from the posterior distribution (model predictions) and, therefore, uncertainty bands are narrower than those seen in Figure 7C. An equivalent plot using *posterior_predict* is included in the Supplementary File 5. Again, a positive causal association between body fat and logHCC is evident, with values being greater and more variable in dogs with comorbidities.

Based on the DAG (Figure 1; Supplementary File 1), the adjustment set required to estimate the causal association between body fat and logHCC, included age, sex, breed group and comorbidity. The vast majority of the probability distribution for the beta coefficient was positive (mean 0.33 per SD; 97% HPDI 0.03, 0.59; Bayes R^2^ 0.18; Figure 7a), and a positive trend was seen across the body fat range (Figure 7b), equating to an average increase in HCC of 5.3 pg/mg for each increase of 1 unit in standardised fat (~10% body fat mass). The relative contributions of our prior expectations and the observed data were assessed in two ways; first, the prior and posterior density distributions of the beta coefficient for body fat from the final model were visually compared (Figure 8a); second, a visual comparison was made of the individual regression lines predicted from 50 random draws taken from the prior (Figure 8b) and posterior (Figure 8c) probability distributions. Prior to observing the data, the model was both neutral and uncertain about the possible effect of body fat on logHCC, as indicated by the mean effect of the prior being 0 (Figure 8a) and the fact that individual regression lines could be either positive or negative to varying degrees (Figure 8b). After seeing the data, the model was both confident of a positive causal association, and relatively confident about its magnitude, as indicated by the narrow range of the posterior distribution (Figure 8a) and the close clustering of the regression line slopes (Figure 8c). Therefore, we estimated a 99% probability of a positive causal association between body fat and logHCC. The conditional effect of body fat on logHCC, stratified by comorbidity and compared with the observed data is shown in Figure 9.

Sensitivity analyses (Supplementary File 5) demonstrated that these results were relatively robust to the effects of measurement error (mean 0.32, 97% HPDI 0.03, 0.60; probability 98%; 5.1 pg/mg mean increase in HCC per 10% body fat mass increase), season of sampling (mean 0.28, 97% HPDI -0.11, 0.62; probability 94%; 4.5 pg/mg mean increase in HCC per 10% body fat mass increase) and missing data (mean 0.27, 97% HPDI 0.01, 0.55; probability 98%; 4.3 pg/mg mean increase in HCC per 10% body fat mass increase.

#### Reverse causality model

**Figure 10:**
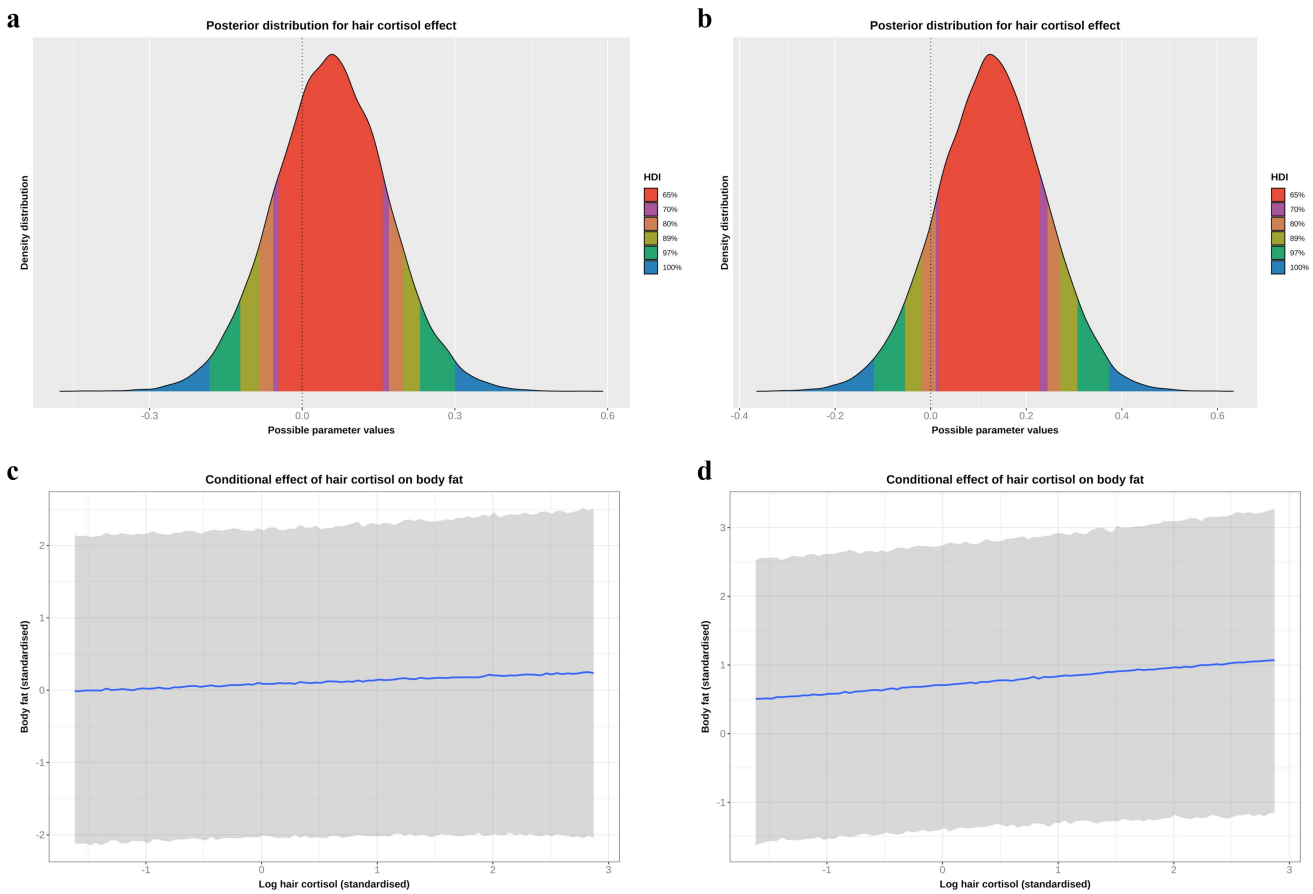
**(a,b)** Highest probability density intervals (a.k.a. highest density interval) plot for the causal association between log hair cortisol concentration (logHCC) and body fat, taken from the reverse causality models and either using all study data **(a)** or only data from visit 0 **(b)**. The y-axis depicts the density distribution, whilst the x-axis depicts possible values for the beta parameter of the causal effect. Different probability intervals are depicted by colour (red 65%; purple 70%; orange 80%; yellow 89%; green 97%; blue 100%). The feint vertical dotted line intersects the x-axis at zero, indicating the point where the beta coefficient would be neither positive of negative. In both models, the posterior probability density spans zero, indicating that the effect could credibly be either positive or negative, albeit with positive effects being more likely given that 70% (**a**: all data) or 87% (**b**: visit 0 data) of the probability density is positive. **(c,d)** Conditional effects for the causal association between logHCC and body fat taken from the reverse causality models and either using all study data **(c)** or only data from visit 0 **(d)**. The blue line represents the mean estimate for logHCC, across the body fat range, whilst the shaded region represents the 95% credible interval. Estimates were generated using the *posterior_predict* function of the ‘brms’ package [version 2.22.0; (62)], which returns the posterior mean and 95% credible interval for each data point, thereby incorporating uncertainty in predictions from the posterior distribution (model predictions) and uncertainty due to residual error (from individual data points). Credible intervals generated in this way reflect the complete range of plausible outcomes from the scientific model. Overall, the linear relationship between logHCC and body fat is relatively flat, with a broad credible interval that includes horizontal).

Although we had assumed that changes in body fat mass would cause changes in HPA function, the possibility of reverse causality, namely that an upregulated HPA would cause increased body fat mass, could not be discounted. To explore this possibility, the DAG was redrawn with HCC and body fat as exposure and outcome, respectively (Supplementary File 1). The same adjustment set as with the body fat model (e.g., age, sex, breed group and comorbidity) was required for this causal analysis, which was tested twice, first using all study data (model 1, Table 5; Figures 10a,c) and also using data from visit 0 only (model 2, Table 5; Figures 10b,d). In both models, posterior probability densities were wide, spanning zero (Figures 10a,b), albeit with slightly more of the probability mass being positive (all data 70%; visit 0 data only 87%). Further, the linear trend was relatively flat across the logHCC range (Figures 10c,d), with the credible interval being broad and including both positive and negative slopes. Such results suggest uncertainty in the estimate of this effect, and would not be consistent with a there being a convincing reverse causality effect.

**Table 5:** Summary of causal associations for the final models where log hair cortisol concentration was the outcome variable.

Reverse causality model	Estimate ^1^	Estimated error ^1^	97% HPDI ^2^	R-hat ^3^	ESS ^4^		Bayes *R*^2 5^	
					Bulk	Tail	Median	97% CI
Model 1	0.06	0.11	−0.16, 0.28	1.00	20,216	17,587	0.43	0.25, 0.56
Model 2	0.13	0.11	−0.10, 0.35	1.00	18,556	16,111	0.25	0.09, 0.40

All analyses used Bayesian multi-level modelling, using the variables and parameters specified in Table 2. All models employed 4 chains, parallelised on separate computer cores, and each using 8,000 iterations (including 2,000 and 6,000 warm-up and sampling iterations, respectively; total available iterations 24,000). ^1^Estimate and estimated error of the beta coefficient for the causal effect of each model. ^2^Highest posterior density interval (a.k,a. highest density interval), the narrowest interval containing the specified probability mass (here 97%), and representing the most probable region of the value of the parameter, given the model, the priors and the observed data. ^3^R-hat is a convergence diagnostic, which compares between- and within-chain estimates for model parameter; values larger than 1 suggest that the Markov chains in the model have not mixed well, with a commonly-accepted cut-off for acceptability being 1.05. ^4^Estimated sample sizes from the Markov Chain Monte Carlo simulation; these are estimates of the number of samples that were used and provide a useful measure of sampling efficiency. As the names suggest, the bulk ESS and tail ESS provide estimates for the sampling efficiency in the bulk and tails of the distribution, respectively; values >100 per Markov chain (here >400) indicate that estimates of respective posterior quantiles are reliable. ^5^Median and 97% compatibility interval of the posterior distribution of the Bayesian *R*^2^ estimate; with this approach, the “variance of the predicted values is divided by the variance of predicted values plus the expected variance of the errors,” with the results “quantifying the fit of the model to the data at hand” (69).

## Discussion

The objective of the present study was to use a Bayesian workflow to estimate possible causal associations between HCC and several different variables in dogs with obesity. The variables for which causal associations were most plausible were body fat and having at least one comorbidity, given the overwhelmingly positive posterior probability densities obtained in the final models. The reasons for a possible positive causal association between body fat and HCC are not clear but a similar association is seen in humans (72). Of course, the causal association between obesity and HPA upregulation might flow in the opposite direction, so-called reverse causation, where upregulated HPA causes an increased body fat mass. Evidence for this includes the fact that cortisol promotes adipose tissue redistribution (to the abdominal region), increases appetite and, in humans at least, promotes a preference for foods of greater palatability (73), all of which could lead to adipose tissue gain. Increased cortisol concentration is also observed in people who gain weight because of stress (74). To address this, we created a final causal model with body fat and logHCC as outcome and causal predictors, respectively, and utilising the same adjustment variables as required for the body fat model (Supplementary File 1). We chose to assess this association both using all study data and only data from visit 0. We included the second analysis because we were concerned that therapeutic weight reduction might affect HCC, as reported in a previous study in dogs (20), thereby obscuring any causal effect of HPA upregulation on body fat mass. In contrast to the result of the body fat model, where the probability distribution for a causal effect was overwhelmingly positive (99%), the probability density spanned zero in both the reverse causality models, i.e., not consistent with a causal effect in this direction. Taken together, these results suggest that, if a causal effect exists, it is most likely to be in the direction of body fat causing HPA upregulation.

For dogs where paired samples were available, mean HCC was 7.6 pg/mg and 3.6 pg/mg in samples taken before and after therapeutic weight reduction, respectively. A major study limitation was the fact that it was not possible to create an unbiased statistical model for the effect of therapeutic weight reduction because, based on our DAG, we could not correct for possible confounding from unmeasured variables. Such unmeasured variables could include owner factors (e.g., owner attitudes and behaviours in implementing a therapeutic weight reduction protocol), environmental factors (e.g., the living environment of the dog) and, of course, the impact of the COVID-19 pandemic; all such variables could plausibly affect both the body fat percentage of dogs and the confounding variables (e.g., affecting attendance of owners at visits and whether an individual dog would successfully reach its target). Considering the findings of the current study, a prospective study should now be considered where the effect of therapeutic weight reduction on HCC could be assessed prospectively.

As well as a possible direct effect of excessive adipose tissue mass on HPA upregulation, obesity might have an indirect effect either by predisposing to or exacerbating other diseases; indeed, many possible disease associations have been identified in previous research (11). In the current study, most dogs had at least one comorbidity, with some having multiple comorbidities. The estimated causal association between comorbidity and HCC was highly variable; not only was HCC greater by an average of 10.4 pg/mg in dogs with comorbidities, but concentrations within the group were much more variable than in the dogs without comorbidities. These findings are, perhaps, not surprising given the many different comorbidities present, with differing severity, and the fact that the pathogenetic mechanisms are likely to be different amongst different diseases. For example, orthopaedic diseases were particularly common in the current study; such diseases might activate stress pathways because of chronic pain, trauma to local tissues caused by the injury or subsequent surgery, inflammation, and by locally triggering endogenous corticosteroid production, as seen in human studies (75–77). Further studies would be required to confirm the mechanisms by which excess adipose tissue leads to increased HCC in dogs.

Possible causal associations were also suggested for some of the other variables, including age and hair coat although posterior probability distributions indicated that such associations were less certain. In this respect, the probability of there being a negative association between age and HCC was estimated to be 88%, whilst the probability of there being negative associations between mixed or dark coat colour and HCC were estimated to be 96 and 93%, respectively. A possible association with coat colour was suggested in previous research (8), and this was the justification for setting a marginally-negative regularising prior in this model. The reason for this association is not fully understood but is likely due to hair-related rather than systemic factors because differences in salivary cortisol are not seen in dogs with different coat colour (8). This has resulted in suggestions that hair of different colour might sequester cortisol to differing degrees, perhaps associated with differences in the size of melanin granules (78). In contrast to the association with hair colour, age has not previously been identified as being associated with HCC in dogs (8), whilst the effect of age in humans is non-linear, being greater in children and elderly, and lowest in middle age (79). Only adult dogs were assessed in the current study (with an age range at the initial visit of 2 to almost 14 years). Therefore, further research would be required, including sampling from growing dogs, to explore more fully a possible causal association between age and HCC in dogs.

There are several limitations that should be considered:

1. The study population was relatively small, with considerable variability in baseline attributes amongst dogs including the presence of comorbidities, and we did not control for the therapy that dogs received. This will have created noise within the dataset, which might have masked the actual causal associations with the variables studied.
2. Having missing data in the body fat percentage variable created challenges for data analysis, although our sensitivity analyses suggested that the impact of this was minimal.
3. It was not possible to examine the causal association between weight loss success and HCC, for example, by creating a binary variable classifying dogs into those that completed and those that ended their therapeutic weight reduction prematurely. Based on our scientific model (Figure 1; Supplementary File 1), it was not possible to identify a set of adjustment variables that allowed this causal association to be estimated. Rather than reporting an erroneous result, with potentially misleading conclusions, we preferred not to attempt such an analysis.
4. It was also not possible to develop a causal model for an effect of visit (before vs. after weight reduction; Figure 1; Supplementary File 1). Therefore, questions of the effect of visit and non-compliance with weight reduction are arguably best answered using a prospective study with an appropriate design.
5. All statistical analyses were based on a scientific model, codified as a DAG, and there might have been errors and omissions, which adversely impact the accuracy of any causal effects.
6. A limitation of the DAG method is that causal pathways typically need to be one-way and cyclical paths are not allowed (i.e., one variable cannot influence other variables which, in turn, influence the first variable). Possible inverse causality in the relationship between changes in body fat and HPA function was considered, as discussed above. However, the direction of association between body fat and having comorbidities is a similar consideration. In our scientific model, we assumed that comorbidities would have an impact on body fat percentage, but the association might be reversed, at least for some diseases. We chose the former rather than the latter on the basis that data on comorbidities were cross-sectional, in that all comorbidities were present at the time of the initial visit. Therefore, the effects of any such comorbidity on body fat mass, for example by affecting food intake or physical activity, would already be evident. In contrast, to test the causal effect of body fat mass on comorbidities, a longer-term cohort study would be needed whereby the effect of initial body fat mass on the future development of a comorbidity could be assessed, as with a recent study examining the association between obesity and future diabetes mellitus (80). Since we did not have body fat data prior to the development of the comorbidities, it would be difficult to study such a causal effect. Further, any monitoring period in such a study period would need to be of sufficient duration to maximise the chances of fully capturing the effect; indeed, a monitoring period of at least 4 years was assessed in the previous study of obesity and DM (80).

Besides these limitations, the findings of the current study should also be interpreted considering possible methodological limitations inherent to hair cortisol analysis. First, HCC can vary with body region and cortisol deposition may not be uniform; in a study involving both humans and other animals, HCC could differ by >20% in hair samples taken from different locations (e.g., head vs. limbs) (81). We controlled for this by always sampling from the same two regions and pooling the harvested hair. These sites were chosen for reasons of convenience since they happened to be regions where clipping was already required for medical procedures (e.g., blood sampling and intravenous catheterisation). Although this approach was favoured for ethical and welfare reasons, our results might not be directly comparable to other studies where different body regions were sampled.

We also did not account for possible variability caused by differences in hair growth rates or the stage of the hair growth cycle, not least since dog hair growth is often cyclical and can be influenced by season (5). Compared with spring, the probability of negative causal associations between summer or autumn and HCC were 77 and 88%, respectively, whilst the probability of a positive causal association between winter and HCC was 83%. Given such probabilities, seasonal effects are certainly possible, but by no means probable. In our scientific model (Figure 1), back door pathways between season of sampling and either body fat percentage or comorbidly were thought to be implausible and, therefore, sampling season was not likely to be a confounder for either variable. These assumptions were confirmed in sensitivity analyses whereby the effects of both body fat percentage and comorbidity were largely unchanged when sampling season was included in modelling.

Finally, there is some evidence for local cortisol production in the skin and hair; for example, in guinea pigs, systemically-administered, radiolabelled cortisol accounted for only a small fraction of the cortisol found in hair, with the remainder probably arising from local follicular synthesis (82). Such local production of cortisol or cortisol-like compounds might also contribute to HCC in dogs, as suggested for dogs with HAC (5). Therefore, factors affecting the skin (e.g., skin inflammation, hyperpigmentation, or topical steroid exposure) might feasibly affect HCC independently of systemic cortisol status.

In conclusion, increased body fat and the presence of one or more comorbidities are causally associated with increased HCC in dogs; prospective studies should assess the impact of therapeutic weight reduction.

## Data Availability

All data generated or analysed during this study are included in this published article and its supplementary information files (Supplementary File 2). The data, statistical code and all statistical reports are also available online at: https://github.com/AliG71/hair_cortisol.

## Footnotes

^1^ https://arriveguidelines.org/arrive-guidelines
