# Supplementary material for: A Bayesian analysis of variables causally associated with hair cortisol concentration in dogs with obesity
Source: Front Vet Sci. 2025 Nov 27;12:1695345. doi: 10.3389/fvets.2025.1695345 (PMC12695548; doi:10.3389/fvets.2025.1695345)
Supplement: Supplementary file 1 [file Data_Sheet_1.pdf]

Supplementary File 1

Directed acyclic graphs

This file includes directed-acyclic graphs (DAG), created using the online resource: https://www.dagitty.net, and based on the scientific model which was used to inform the statistical analyses. The first DAG is the final model depicting the outcome variable (marked "I"), observed variables (blue circles) and unobserved variables (grey). Most of the subsequent DAGs show the adjustment sets required for each of the final models to enable the causal effect to be determined. The top pane shows the outcome variable ("I"), causal predictor (marked with a triangle), causal pathways (green arrows), confounding variables (red circles), confounding (i.e., backdoor) pathways (red arrows), variables not on a causal or backdoor pathway (blue circles) and unobserved variables (grey circles). To fulfil the 'backdoor criterion' (Pearl), and estimate the causal effect, an adjustment set of variables is required that ensures all backdoor pathways are blocked, as shown in the lower pane. A similar colour coding is used here, except that adjusted variables (e.g., those included in the final model) are shown as white circles. As can be seen, there are no remaining confounding paths, when adjusting for such a set of variables.

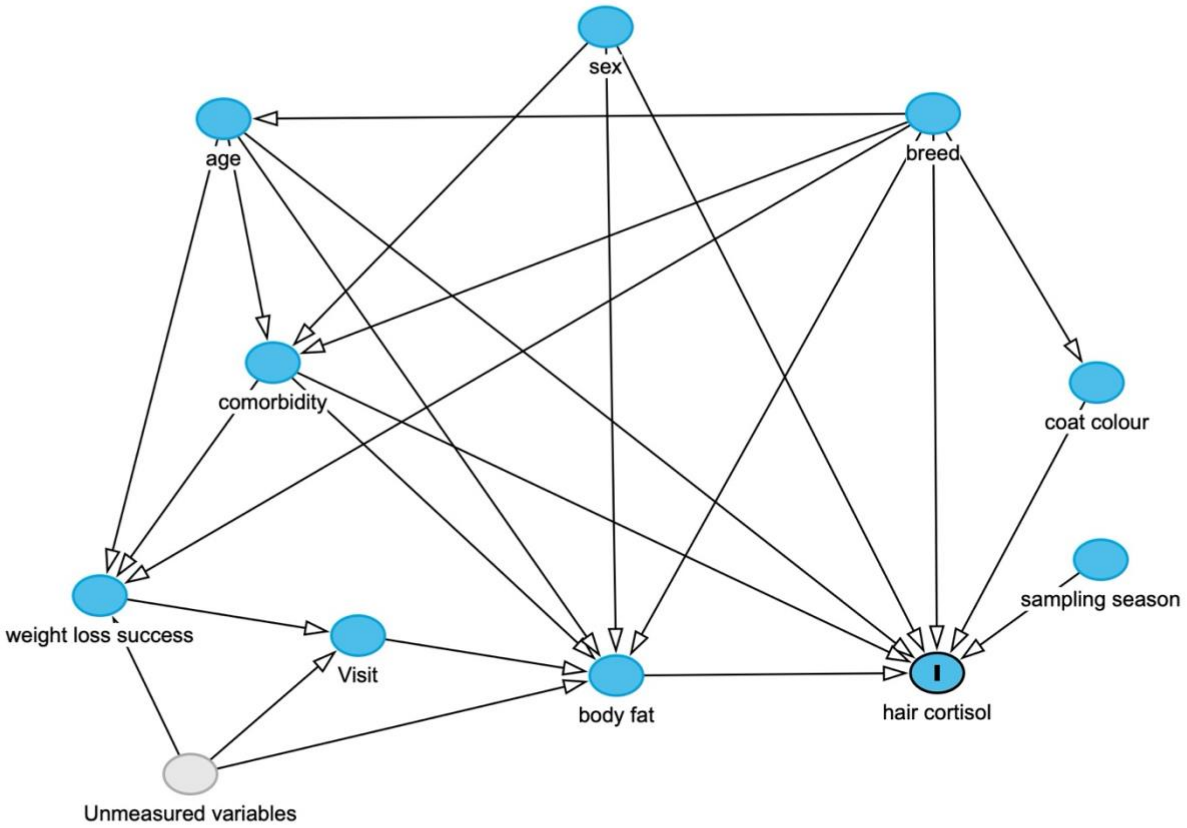

Fig 1. Directed acyclic graph showing the assumptions of the scientific model that informed the statistical analyses used to determine causal effects of different variables on hair cortisol concentration.

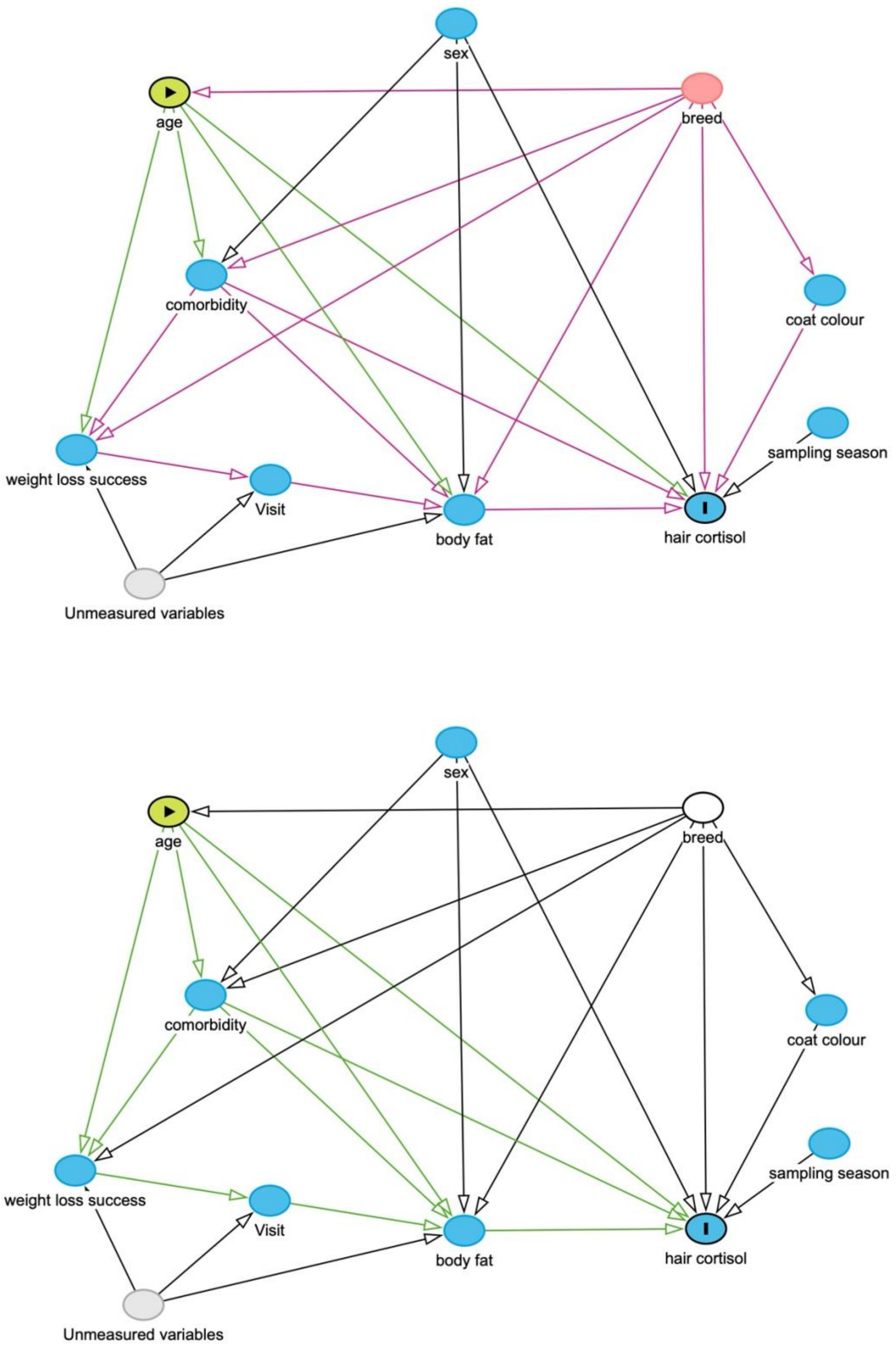

Fig 2. Final DAG for the causal effect of age on hair cortisol concentration. A single adjustment variable (breed) was required.

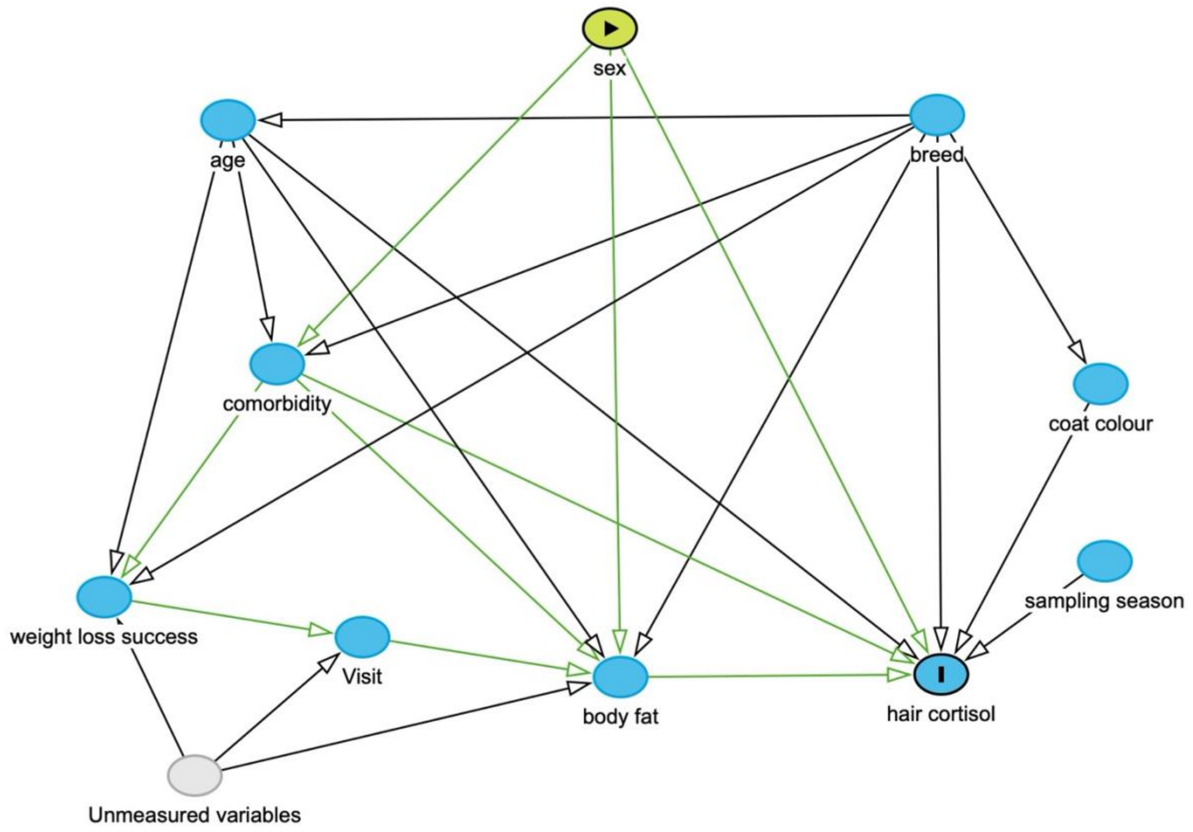

Fig 2. Final DAG for the causal effect of sex on hair cortisol concentration. No adjustment variables were required

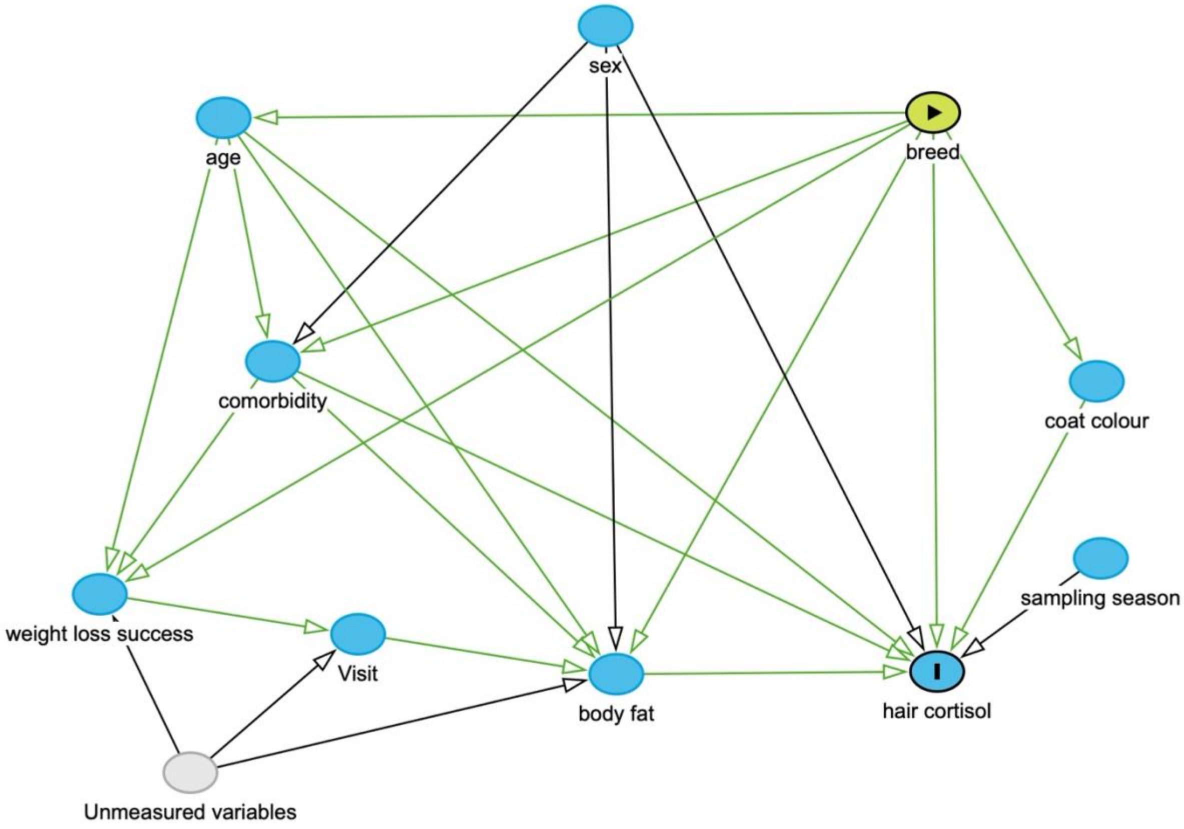

Fig 3. Final DAG for the causal effect of breed on hair cortisol concentration. No adjustment variables were required.

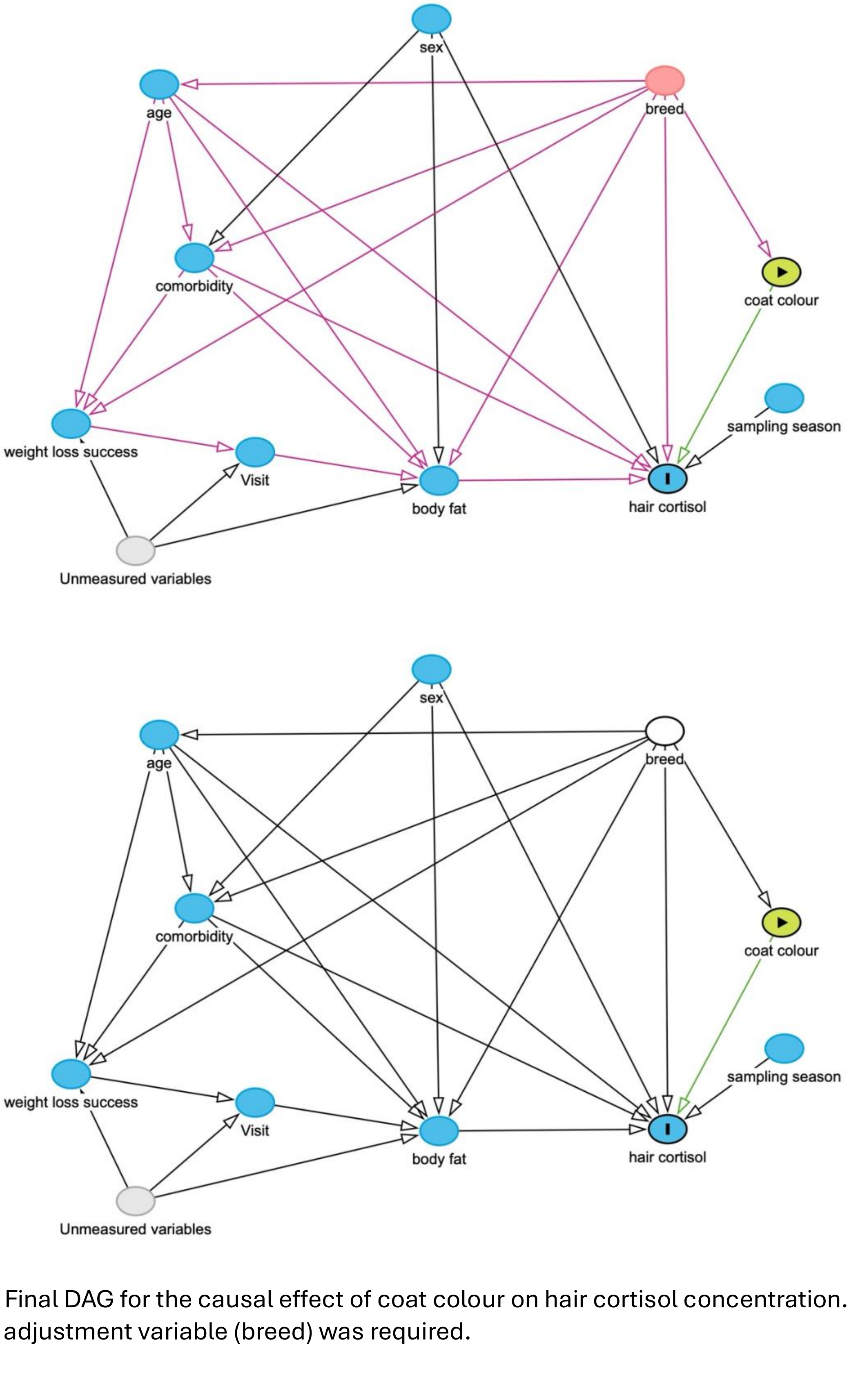

Fig 4. Final DAG for the causal effect of coat colour on hair cortisol concentration. A single adjustment variable (breed) was required.

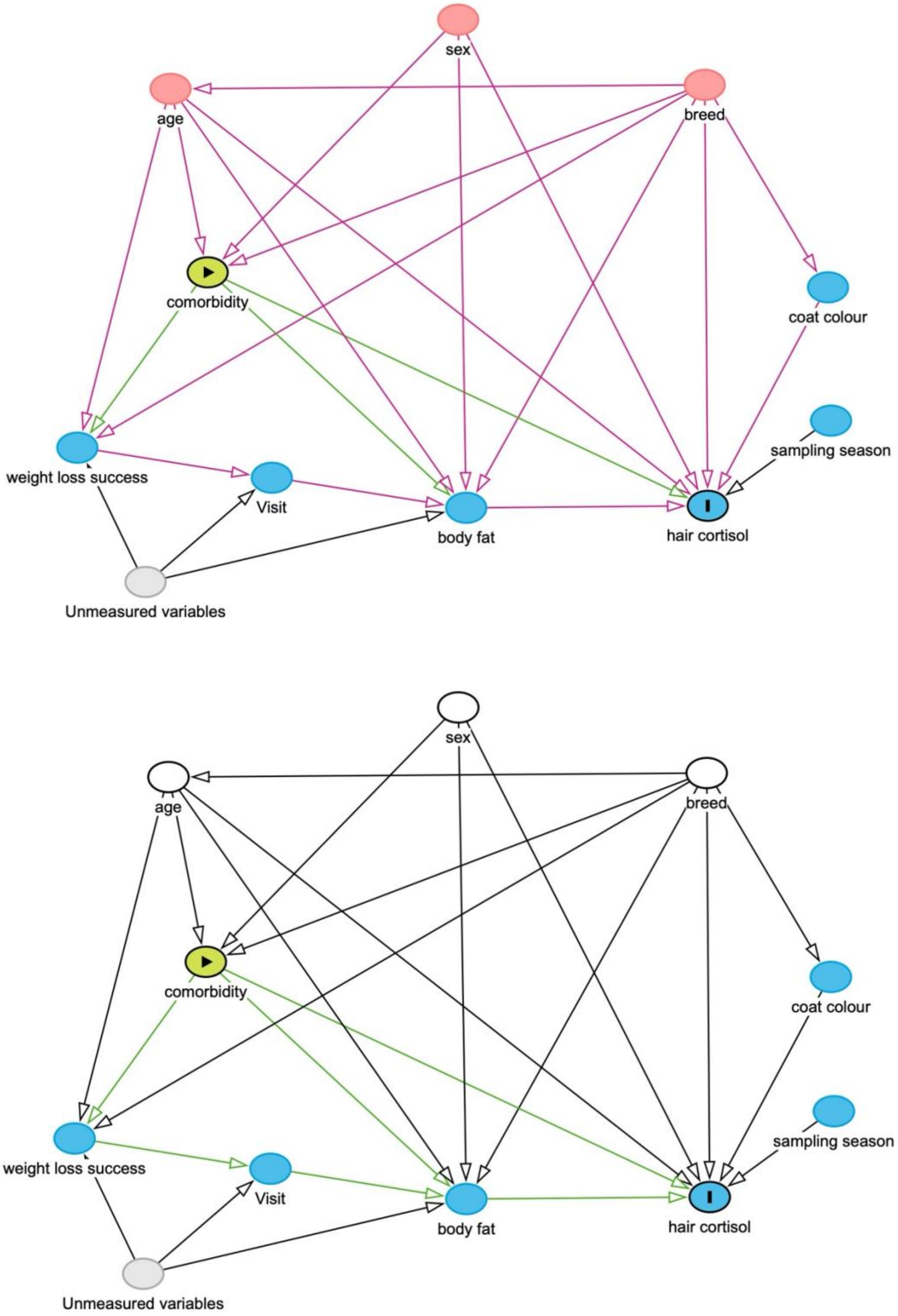

Fig 5. Final DAG for the causal effect of comorbidity on hair cortisol concentration. The required adjustment set was age, breed and sex.

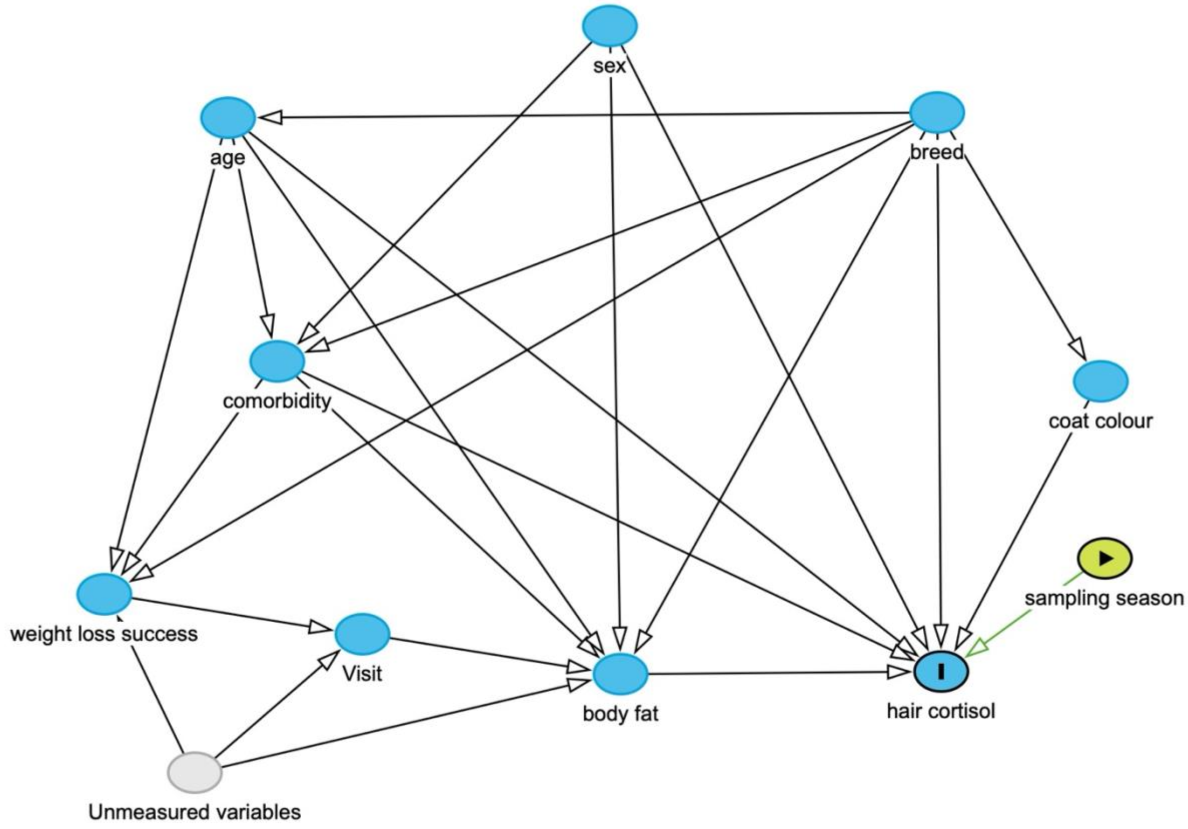

Fig 5. Final DAG for the causal effect of season of sampling on hair cortisol concentration. No adjustment variables were required.

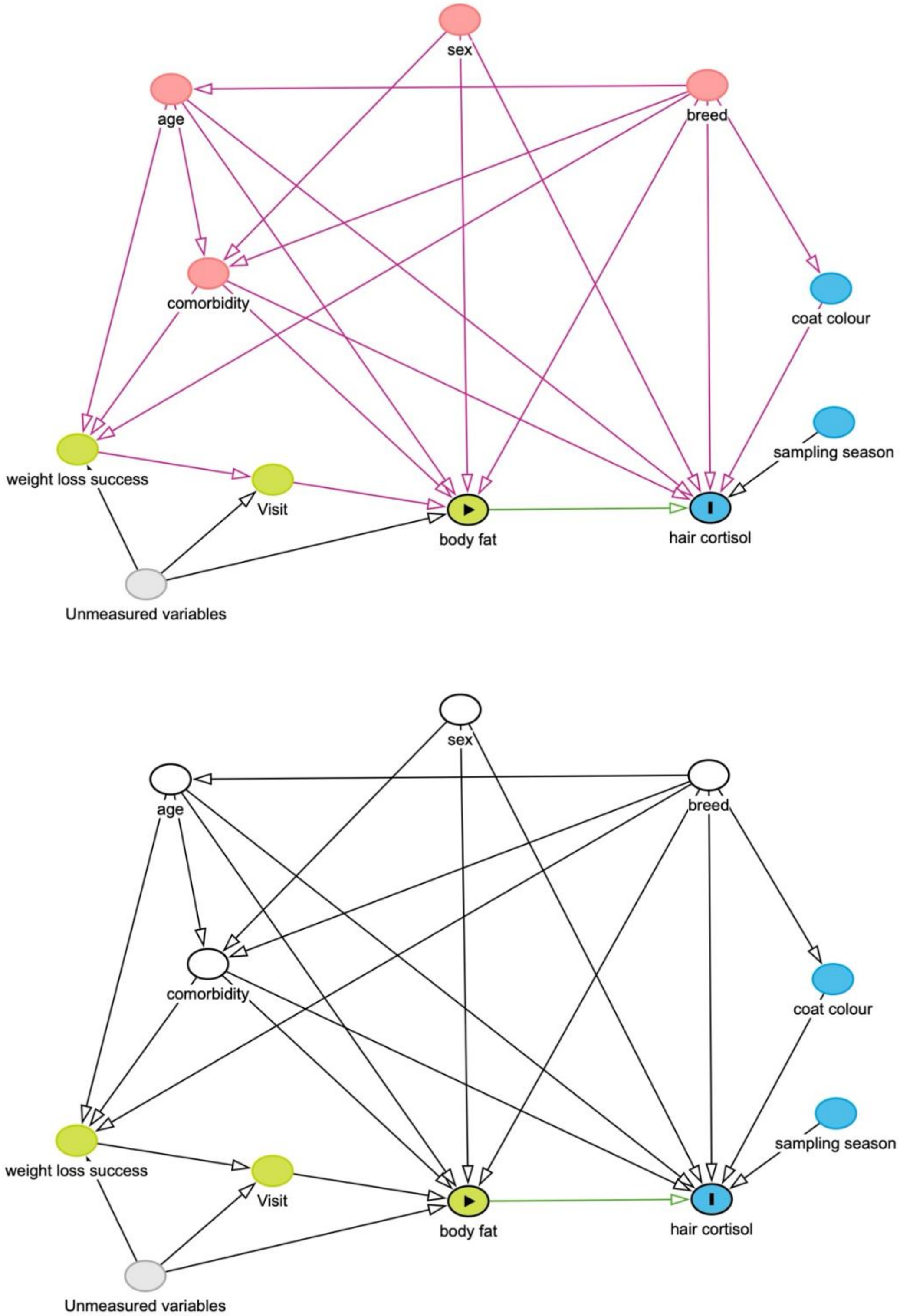

Fig 5. Final DAG for the causal effect of body fat mass on hair cortisol concentration. The required adjustment set was age, breed, sex and comorbidity.

Successful weight loss
Exposure: successful weight loss
Outcome: hair cortisol
Biasing paths are open.
No adjustment sets found.

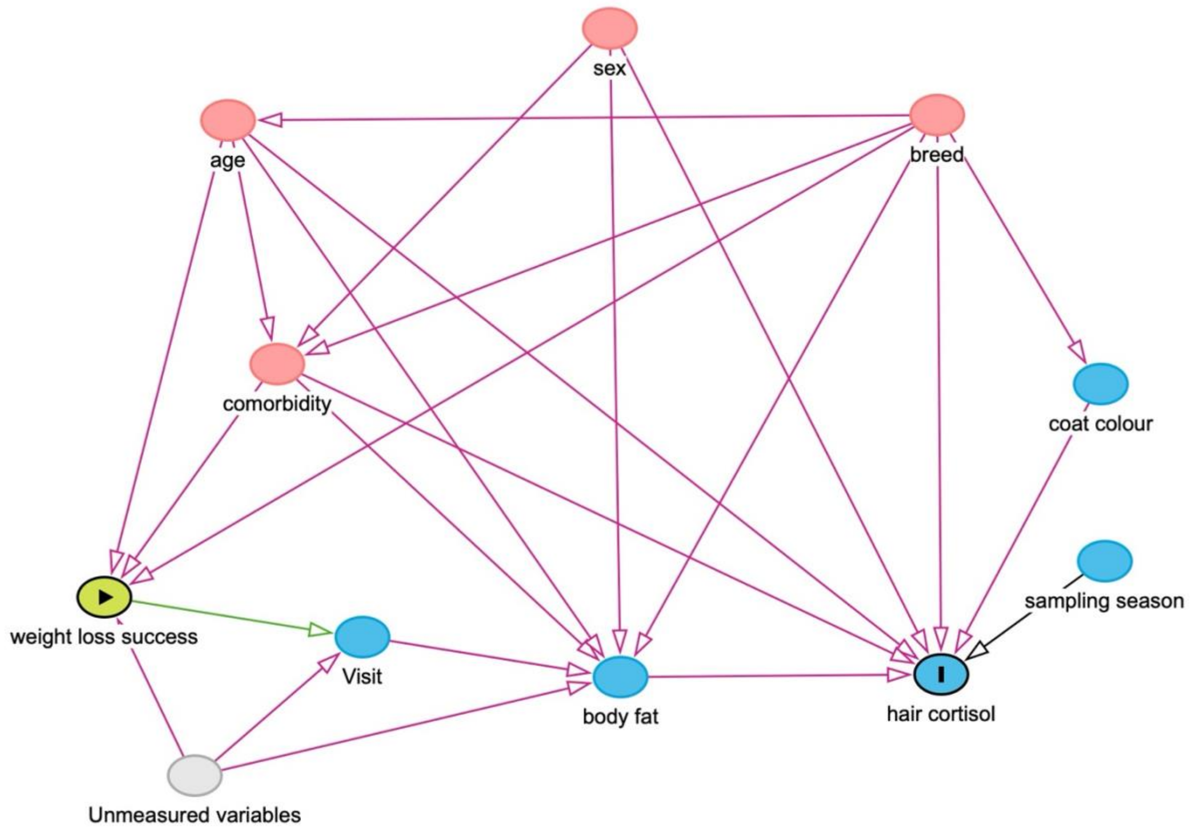

Fig 6. Final DAG for the causal effect of successful weight loss on hair cortisol concentration. Unfortunately, an estimate the causal effect of successful weight loss (completed vs. stopped groups) could not be made because it was not possible to close all backdoor paths (to fulfil the backdoor criterion) on account of their being unobserved confounding variables.

Visit
Exposure: visit
Outcome: hair cortisol
Biasing paths are open.
No adjustment sets found.

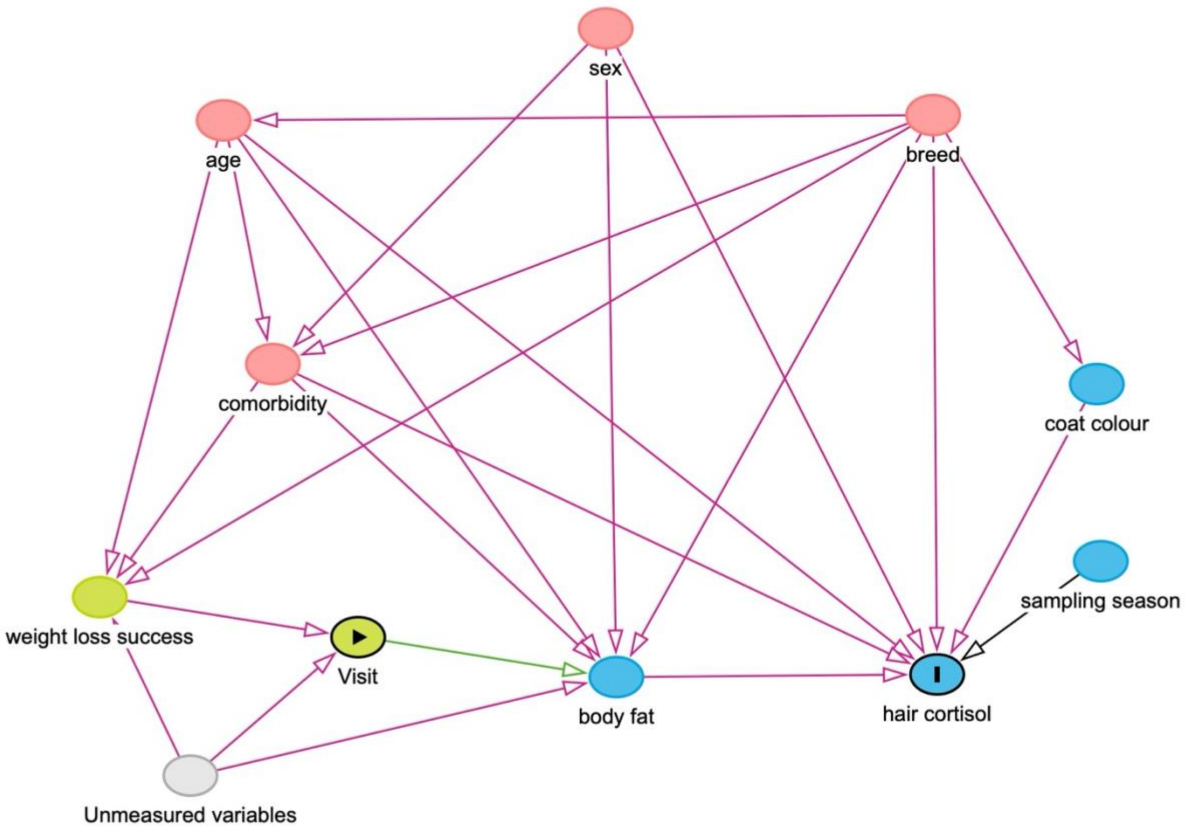

Fig 7. Final DAG for the causal effect of visit (before [v0] vs after [v1] therapeutic weight reduction) on hair cortisol concentration. It was not possible to identify an appropriate adjustment set due to the presence of unmeasured confounding variables in the DAG, creating confounding pathways that could not be closed (backdoor criterion not fulfilled).

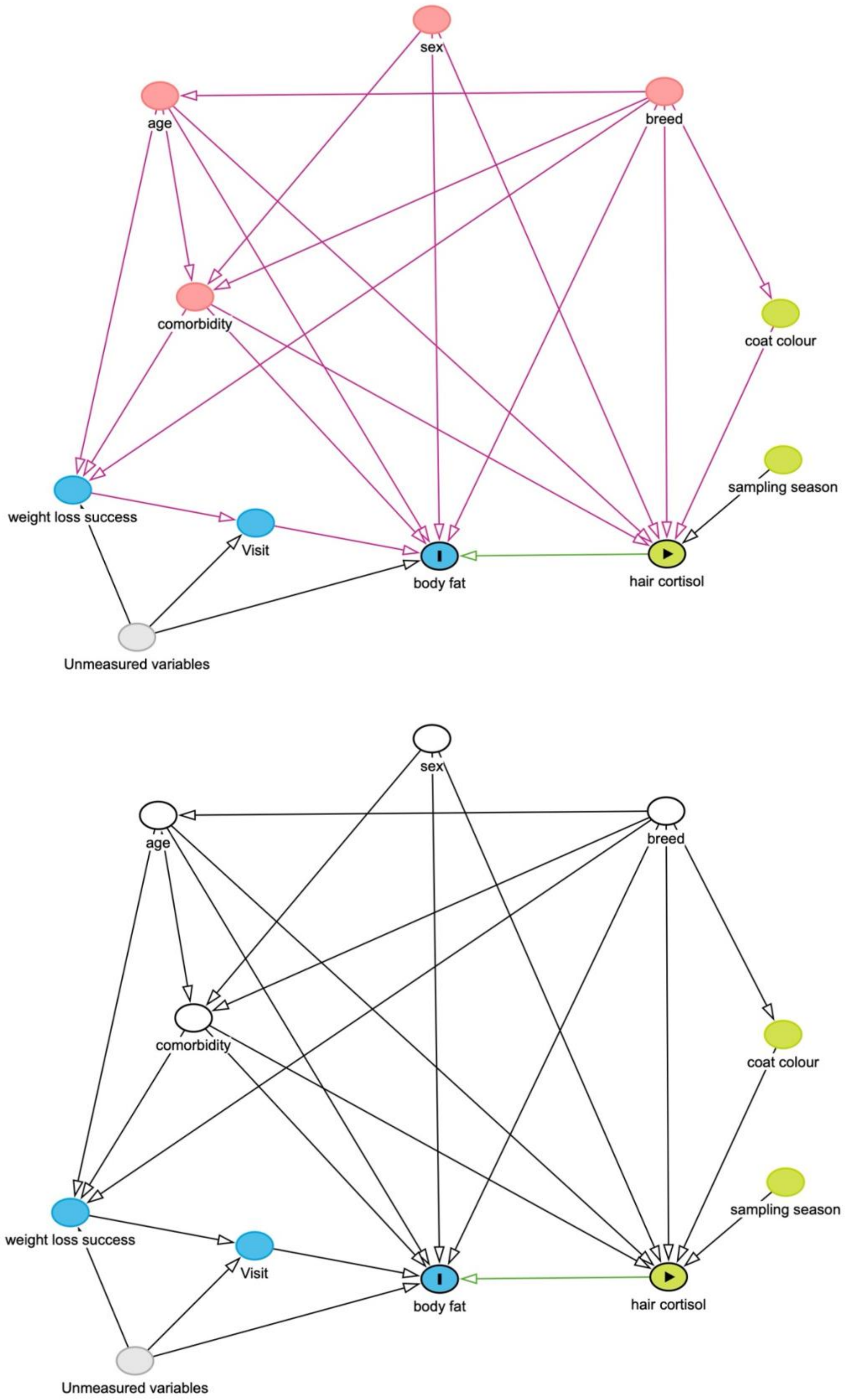

Fig 8. Final DAG for the reverse causality model, assessing for a possible effect of hair cortisol concentration on body fat mass. The required adjustment set was age, breed, sex and comorbidity.
