# Supplementary material for: A Bayesian analysis of variables causally associated with hair cortisol concentration in dogs with obesity
Source: Front Vet Sci. 2025 Nov 27;12:1695345. doi: 10.3389/fvets.2025.1695345 (PMC12695548; doi:10.3389/fvets.2025.1695345)
Supplement: Supplementary file 3 [file Data_Sheet_3.pdf]

Supplementary File 3

Further details of Bayesian analyses

Model computation
Bayesian analyses were computed using the 'brms' package (version 2.22.0, [1]), which fits multilevel Bayesian models using the probabilistic programming language, 'Stan' [2], accessed via the 'rstan' package (version 2.32.7, [3]). Stan estimates posterior probabilities by implementing Hamiltonian Monte Carlo [4] and its extension, the No-U-Turn Sampler (NUTS; [5]). These algorithms are superior to many other (MCMC) algorithms because they converge more quickly, thereby improving efficiency [5]. The 'brms' package combines the convenience of using similar syntax to other R packages (e.g., 'lme4' [6]), whilst supporting a wide range of distributions and link functions including those that are robust to outliers, thereby avoiding overfitting. Finally, models can readily be evaluated, either by in-built tools or by using several compatible packages including 'bayesplot' [7], 'bayestestR' [8], 'loo' [9], 'priorsense' [10] and 'rethinking' [11].

All models employed 4 chains, parallelised on separate computer cores, and each using 8,000 iterations (including 2,000 and 6,000 warm-up and sampling iterations, respectively). Diagnostic checks performed included checks of MCMC performance, as well as several prior and posterior validation checks. Examples of these checks are shown in supplementary information (Supplementary File 3); full details of the diagnostics from all models (including the code used and all statistical output) are available online: https://github.com/AliG71/hair_cortisol.

Verification checks
MCMC performance
To verify MCMC performance, models were checked for convergence, by assessing R-hat values and inspecting both trace and trace-rank plots (See examples below), whilst resolution was assessed by calculating effective sample sizes. Occasional problems with convergence or divergent chains were resolved by altering NUTS control parameters (e.g., amending step size, maximum treedepth or adapt_delta), or by adjusting the regularising ability of priors (e.g., altering SD [normal distribution] or rate [exponential distribution] parameters). In all final models, such steps resolved any problems encountered, and effective sample sizes were always acceptable (typically 10,000-20,000 depending upon the model).

Verification of priors
Verifications of the suitability of prior probabilities included initial graphical modelling, to simulate the expected shape of the distribution, and prior predictive simulations utilising models that sampled from the prior probability distributions only. Results were graphically modelled using the pp_check function of the 'bayesplot' package [63] (see examples below), and by creating graphical visualisations of slopes using random draws of intercepts and beta parameters from the prior distributions. Power scaling sensitivity analysis (powerscale_sensitivity function of the 'priorsense' package [10])

was used to identify the sensitivity of priors and likelihoods to power-scaling, as well as identifying possible prior-likelihood conflicts. For this, the Cumulative Jensen-Shannon distance was used as the divergence method, and the sensitivity threshold set to 0.05, with larger values being flagged as potentially sensitive to power scaling [10]. Finally, the check_prior function of the 'bayestestR' package [8] was used as a simple check to determine whether priors for each parameter were informative.

Verification of posterior probability distributions
Verification checks on the posterior distributions included a visual inspection of a pairs plot (to determine the presence of covariance amongst model parameters); a graphical posterior predictive check (to confirm that the model usefully mimicked the observed data); graphical comparisons of individual draws against the observed data for each predictor variable (to confirm that predicted effects of each variable were consistent with the observed data); and several other graphical checks to compare observed data versus simulated data from the posterior predictive distribution (e.g., error histograms, scatter plots of joint posteriors, scatter average plots, and a leave-one-out predictive check; see examples below).

References

1. Bürkner PC. Advanced Bayesian multilevel modeling with the R package brms. The R Journal. (2018) 10(1): 395-411. doi:10.32614/RJ-2018-017.
2. Stan Development Team (2025). Stan Modeling Language: User's Guide and Reference Manual. URL https://mc-stan.org/docs/stan-users-guide/ [Accessed July 31, 2025].
3. Gabry J, Goodrich B, Lysy M, Johnson A (2024). rstantools: Tools for developing R packages interfacing with 'Stan'. R package version 2.4.0. 2024. https://CRAN.R-project.org/package=rstantools [Accessed July 31, 2025]
4. Duane S, Kennedy AD, Pendleton BJ, Roweth D. Hybrid Monte Carlo. Physics Letters B. (1987) 195(2): 216–222.
5. Hoffman MD, Gelman A. The No-U-Turn Sampler: adaptively setting path lengths in Hamiltonian Monte Carlo. The Journal of Machine Learning Research. (2014) 15(1): 1593–1623. https://jmlr.org/papers/volume15/hoffman14a/hoffman14a.pdf
6. Bates D, Maechler M, Bolker B, Walker S. Fitting Linear Mixed-Effects Models Using lme4. Journal of Statistical Software. (2015) 67(1): 1-48. doi: 10.18637/jss.v067.i01.
7. Gabry J, Simpson D, Vehtari A, Betancourt M, Gelman A. Visualization in Bayesian workflow. Journal of the Royal Statistical Society Series A: Statistics in society. (2019) 182: 389-402. doi:10.1111/rssa.12378 <https://doi.org/10.1111/rssa.12378.
8. Makowski D, Ben-Shachar M, Lüdecke D. bayestestR: describing effects and their uncertainty, existence and significance within the Bayesian framework. Journal of Open Source Software. (2019) 4(40): 1541. doi:10.21105/joss.01541.
9. Vehtari A, Gelman A, Gabry J. Practical Bayesian model evaluation using leave-one-out cross-validation and WAIC. Statistics and Computing. (2017) 27: 1413-1432. doi:10.1007/s11222-016-9696-4.

10. Kallioinen N, Paananen T, Bürkner P-C, Vehtari A. Detecting and diagnosing prior and likelihood sensitivity with power-scaling. Statistics and Computing. (2023) 34(57). doi:10.1007/s11222-023-10366-5.
11. McElreath R (2024) rethinking: Statistical Rethinking book package. R package version 2.42, commit ac1b3b2cda83f3e14096e2d997a6e30ad109eeee. https://github.com/rmcelreath/rethinking [Accessed July 31, 2025]

Examples of various graphical validation checks performed on models

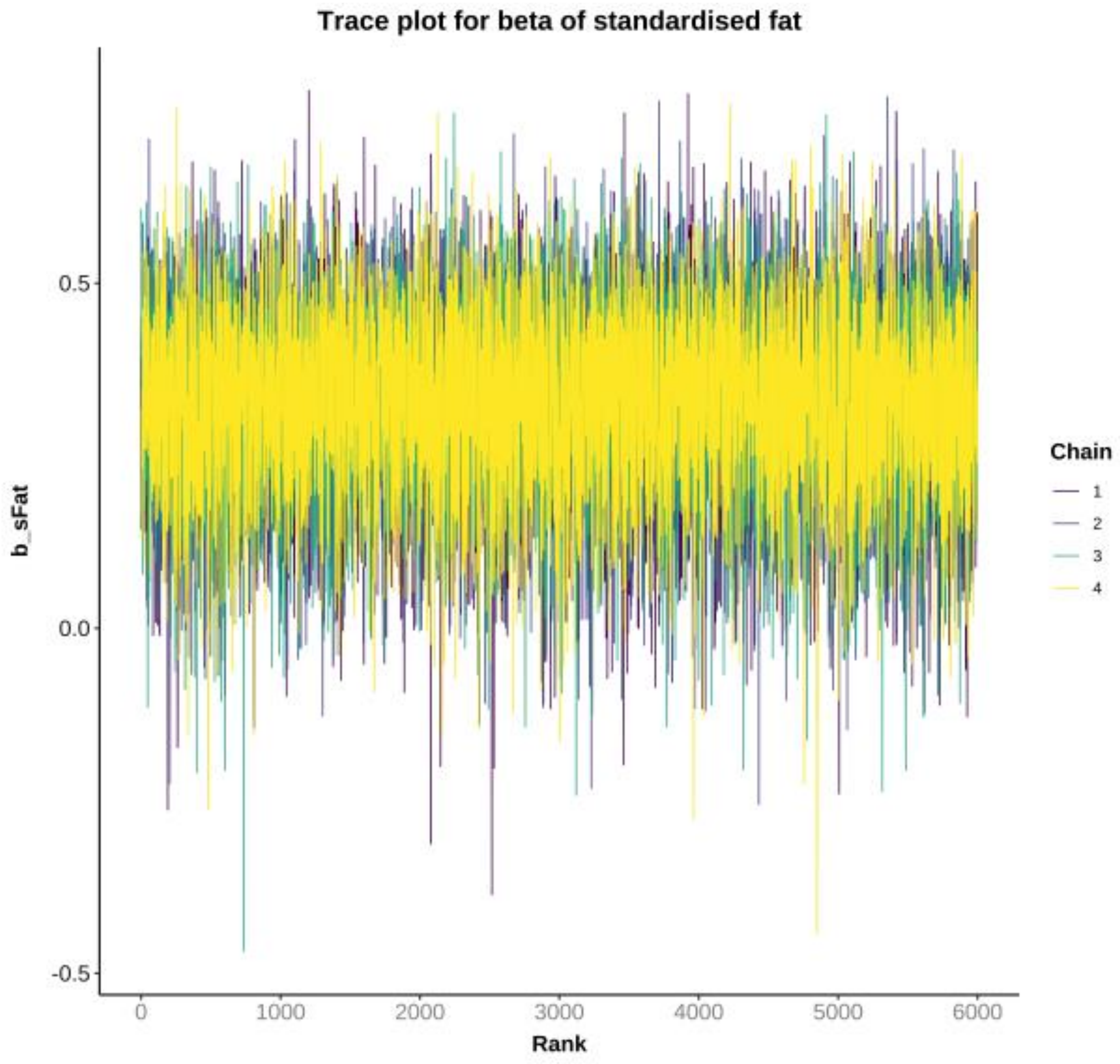

Fig 1. Trace plot for the beta coefficient for the effect of body fat on log hair cortisol concentration (both standardised). Such a plot displays values for model parameters sampled by four concurrent Markov Chain Monte Carlo (MCMC) algorithms (depicted by different colours, as indicated) over a series of iterations, enabling chain stability and mixing to be evaluated; MCMC chains are confirmed to be stable (for sampling purposes) when they reach a stable state (horizontal position, are adequately exploring the parameter space, with good mixing amongst chains (when they appear as 'hairy caterpillars').

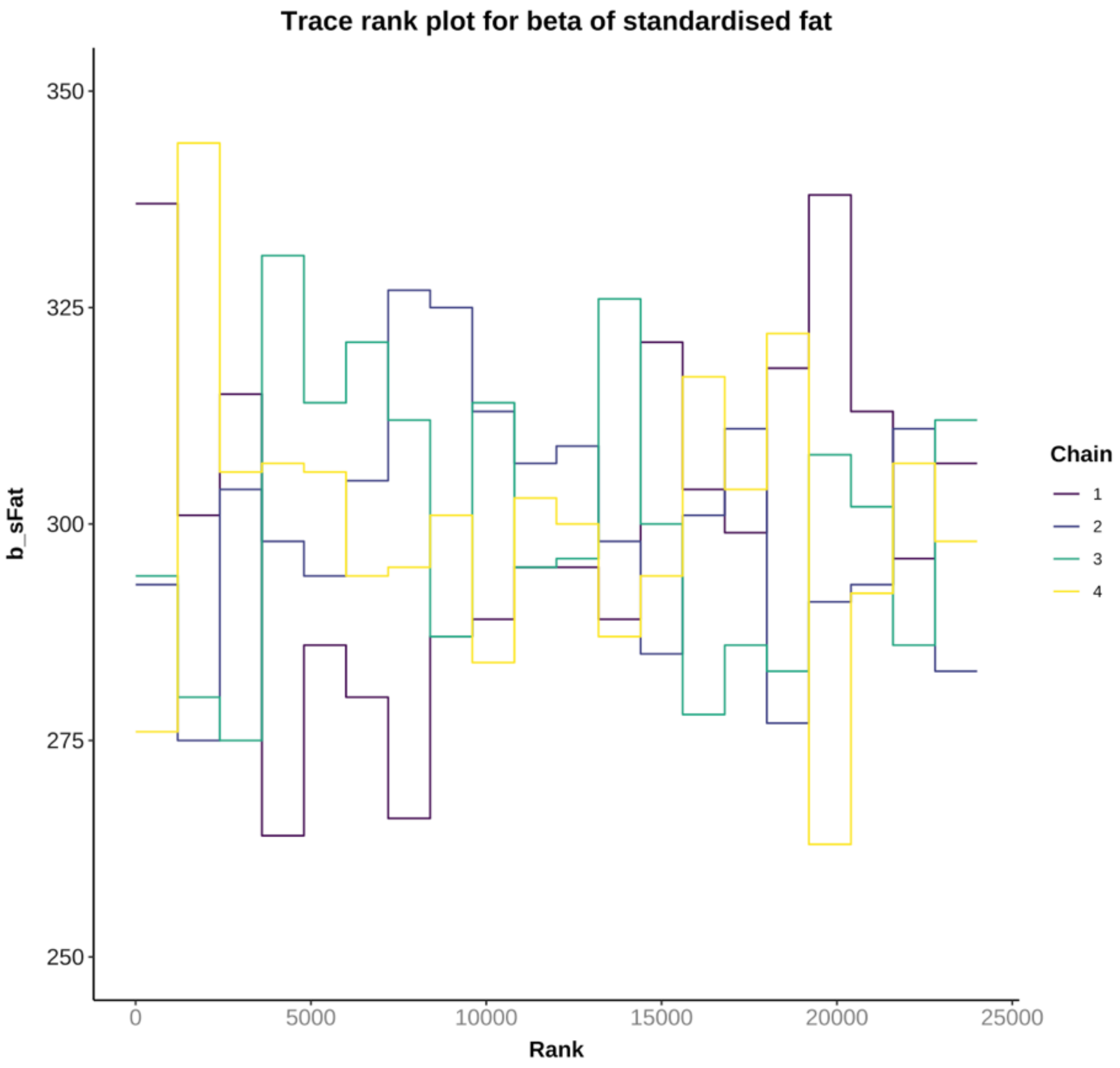

Fig 2. Trace (rank) plot (a.k.a. “Trank” plot for the beta coefficient for the effect of body fat on log hair cortisol concentration (both standardised). This plot is similar to the trace plot shown in Fig 1 but, rather than showing the parameter space, displays ranks of values from the chains. As such it can often provide a clearer indication of how well the chains are mixing (depicted as an approximately uniform histogram with overlapping chains); in contrast, non-uniform histograms (where one or more chains that is constantly greater or less than the others) suggest the chains are not mixing well and might require further investigation.

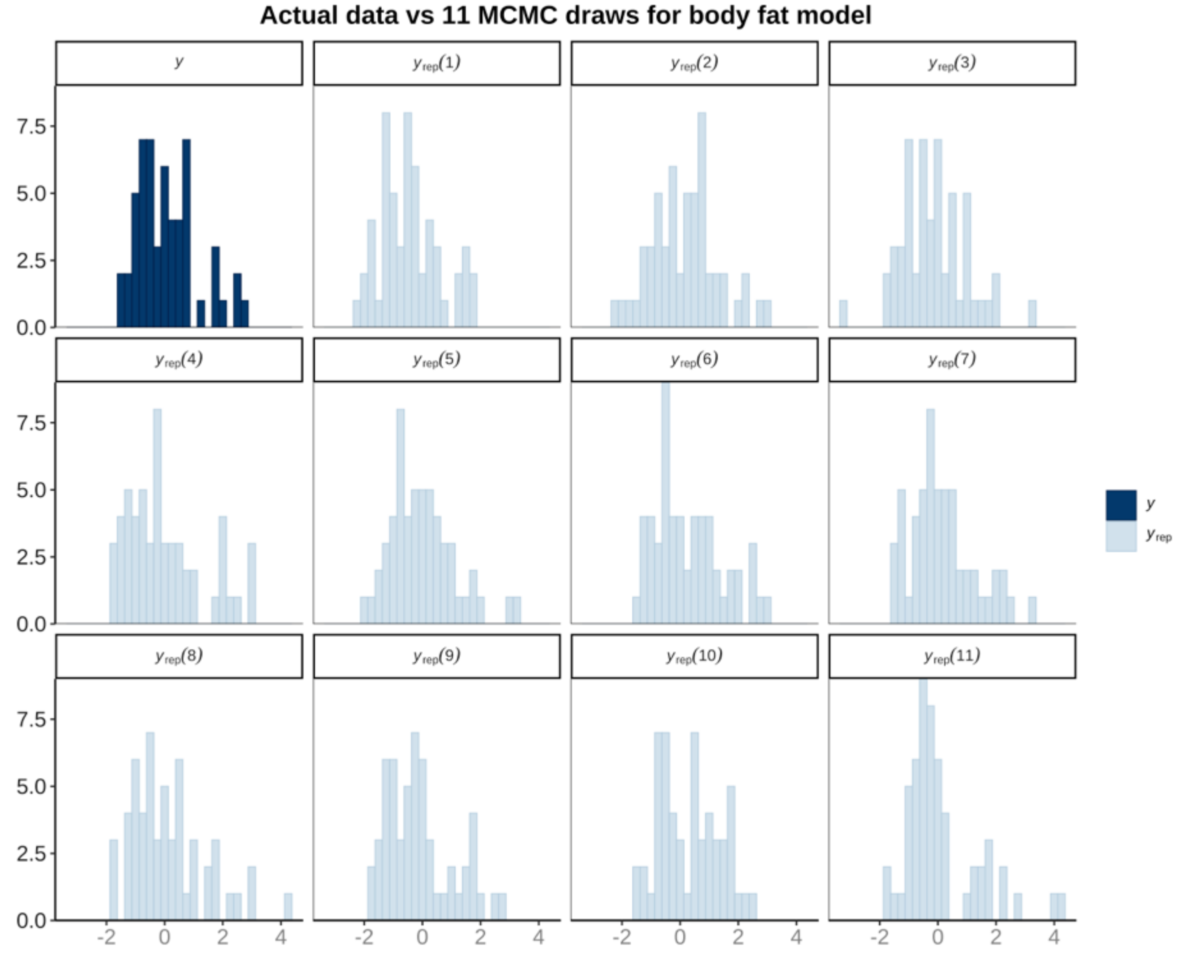

Fig 3. Comparison of histograms of the observed log hair cortisol concentrations and individual predictions using draws from the posterior probability distributions of the final model. The distributions for each of the individual predictions (feint blue) are similar to the distribution of the data itself (dark blue).

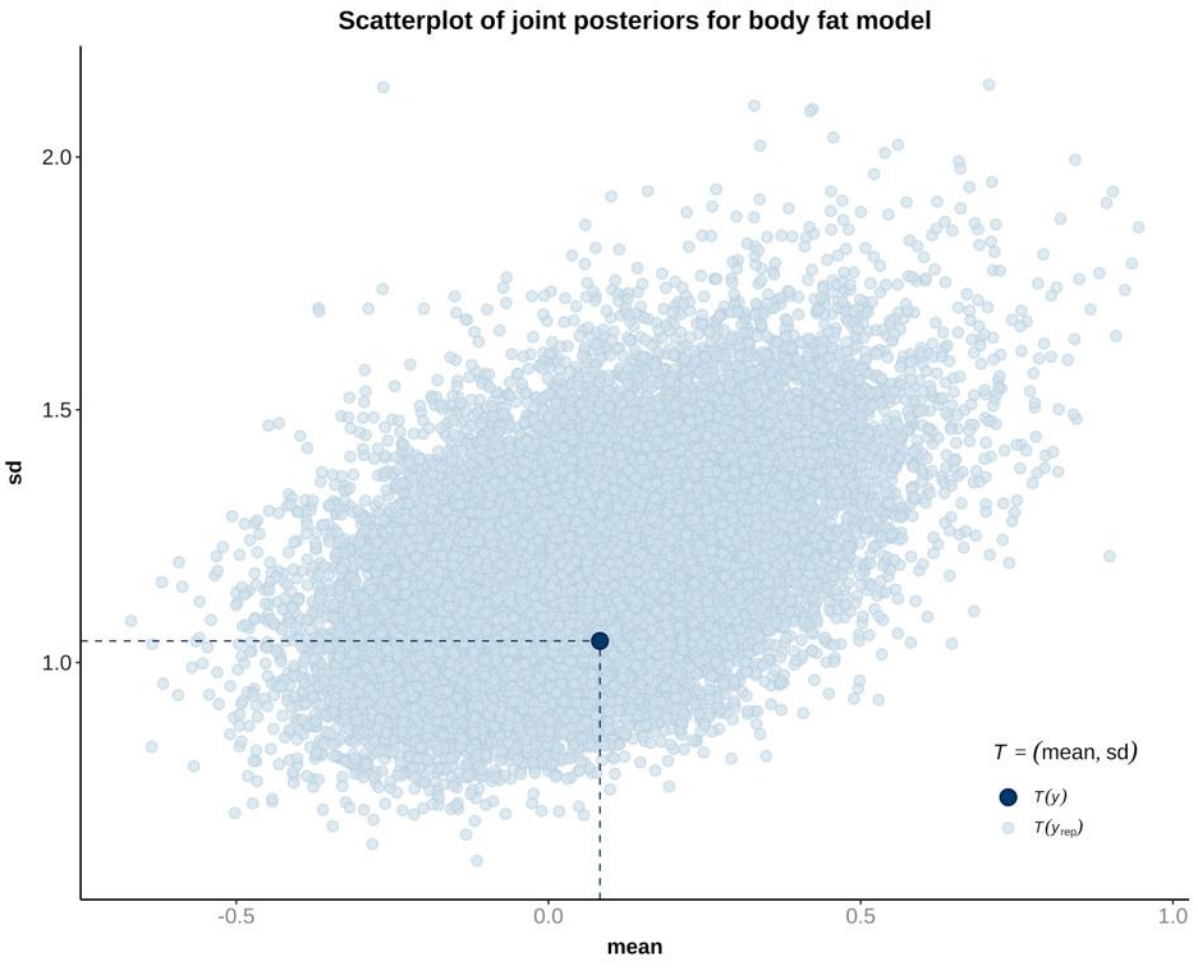

Fig 4. Bivariate scatterplot of posterior draws from the body fat mode. Such plots help to illustrate relationships between pairs of parameters estimated from Markov Chain Monte Carlo (MCMC) analysis, and are another means of assessing convergence. When chains have mixed well and have adequately explored the parameter space, the scatter plot will show a stable and dense region, which indicates convergence, and with no evidence of correlation between parameters.

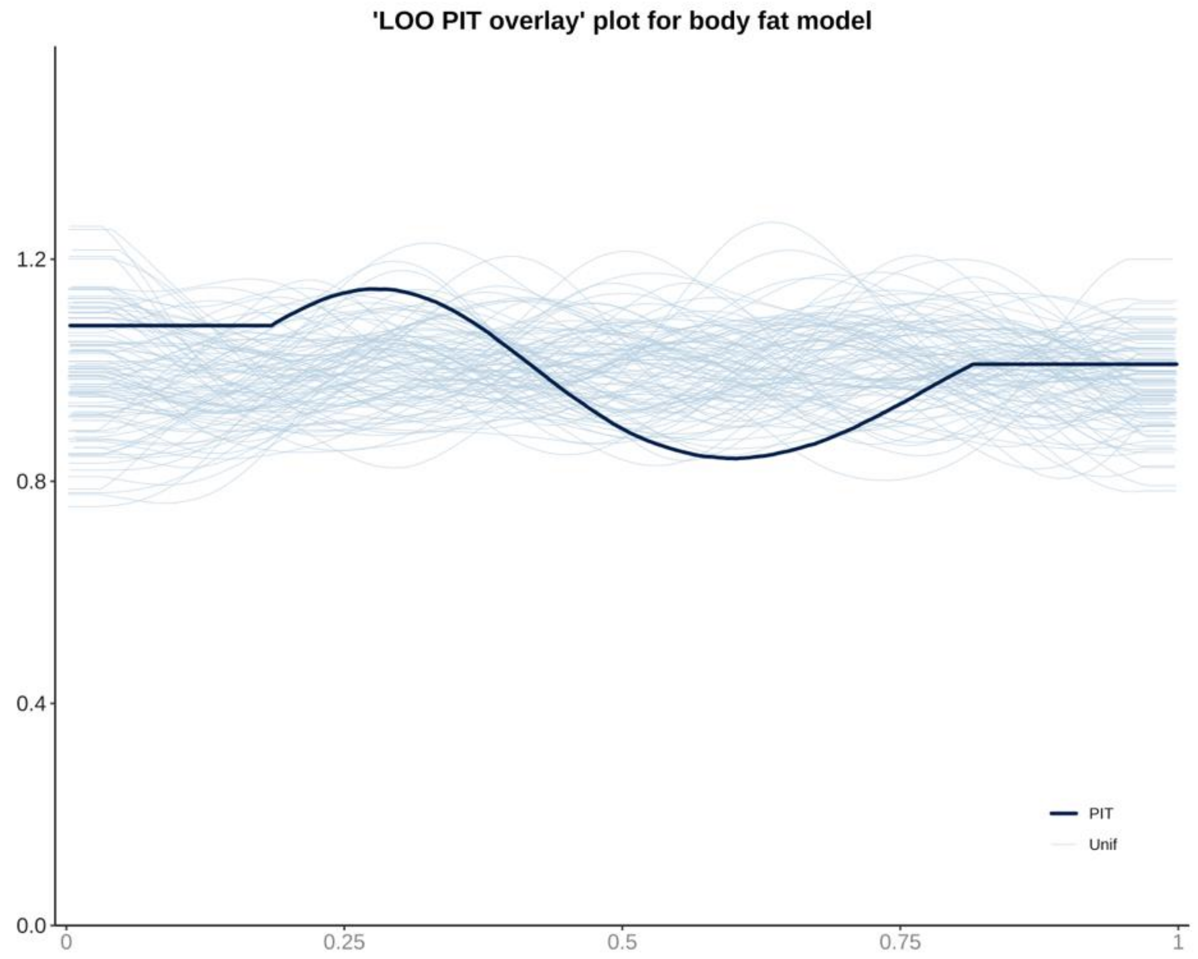

Fig 5. Probability integral transformation (PIT) check, adjusted using leave-one-out cross validation, which results in better calibration by avoiding double-counting of data. This plot assesses how well calibrated marginal predictions are, with models being asymptotically uniform (for continuous data) if the model is calibrated. In this plot, the density of the LOO PITs (“PIT”; thick blue line) has been compared with the density estimates of many simulated data sets from the standard uniform distribution (“Unif” thin feint blue lines).

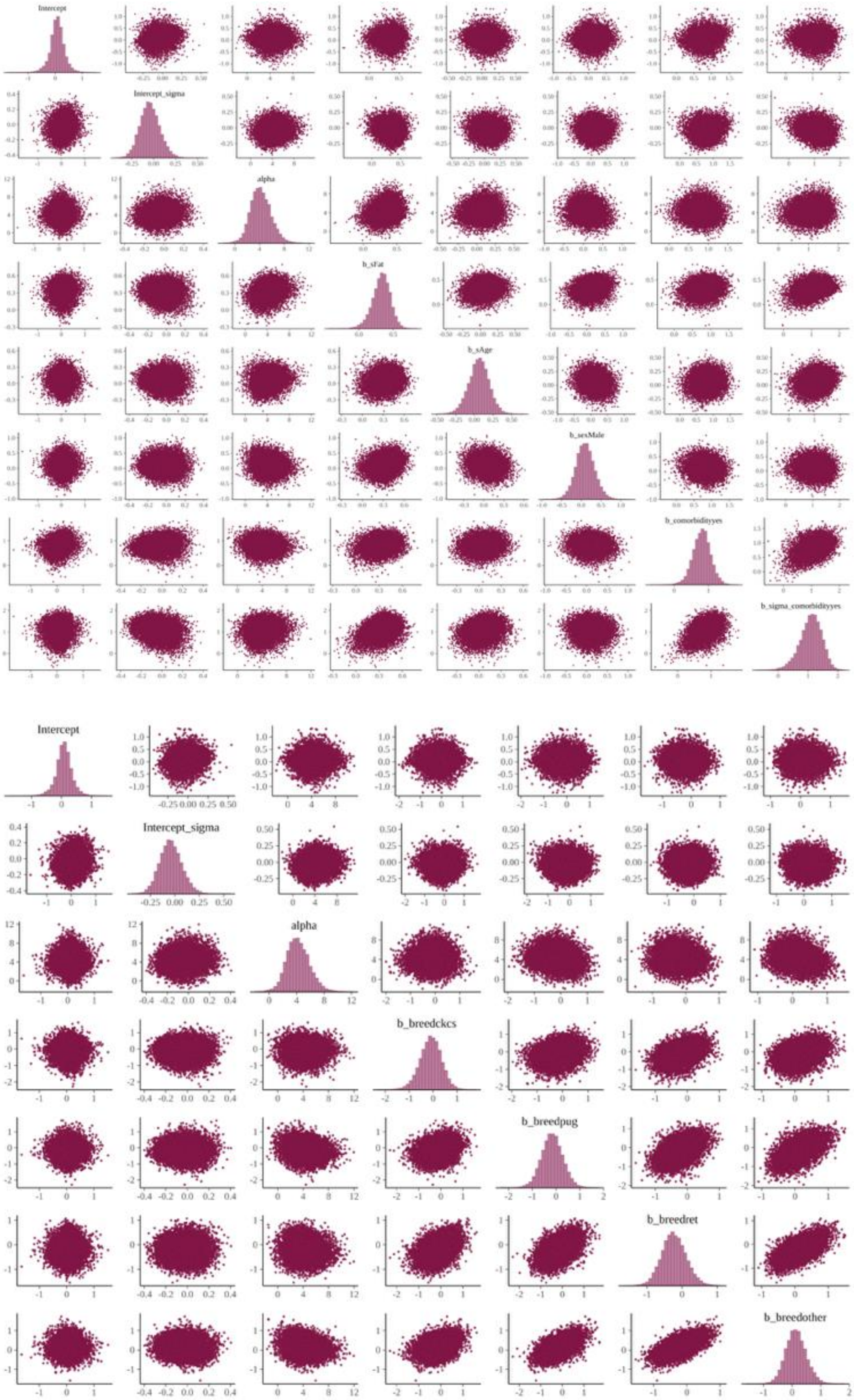

Fig 6. Pairs plot of parameters from the body fat model, displaying histograms and bivariate scatter plots for selected parameters, and enabling associations to be explored. Here, parameters here have been separated into two groups because plotting >8 parameters simultaneously makes them harder to view. Such plots are especially useful for identifying collinearity between variables. Of course, some collinearity is inevitable, for example amongst separate categories of the same variable (e.g., breed).

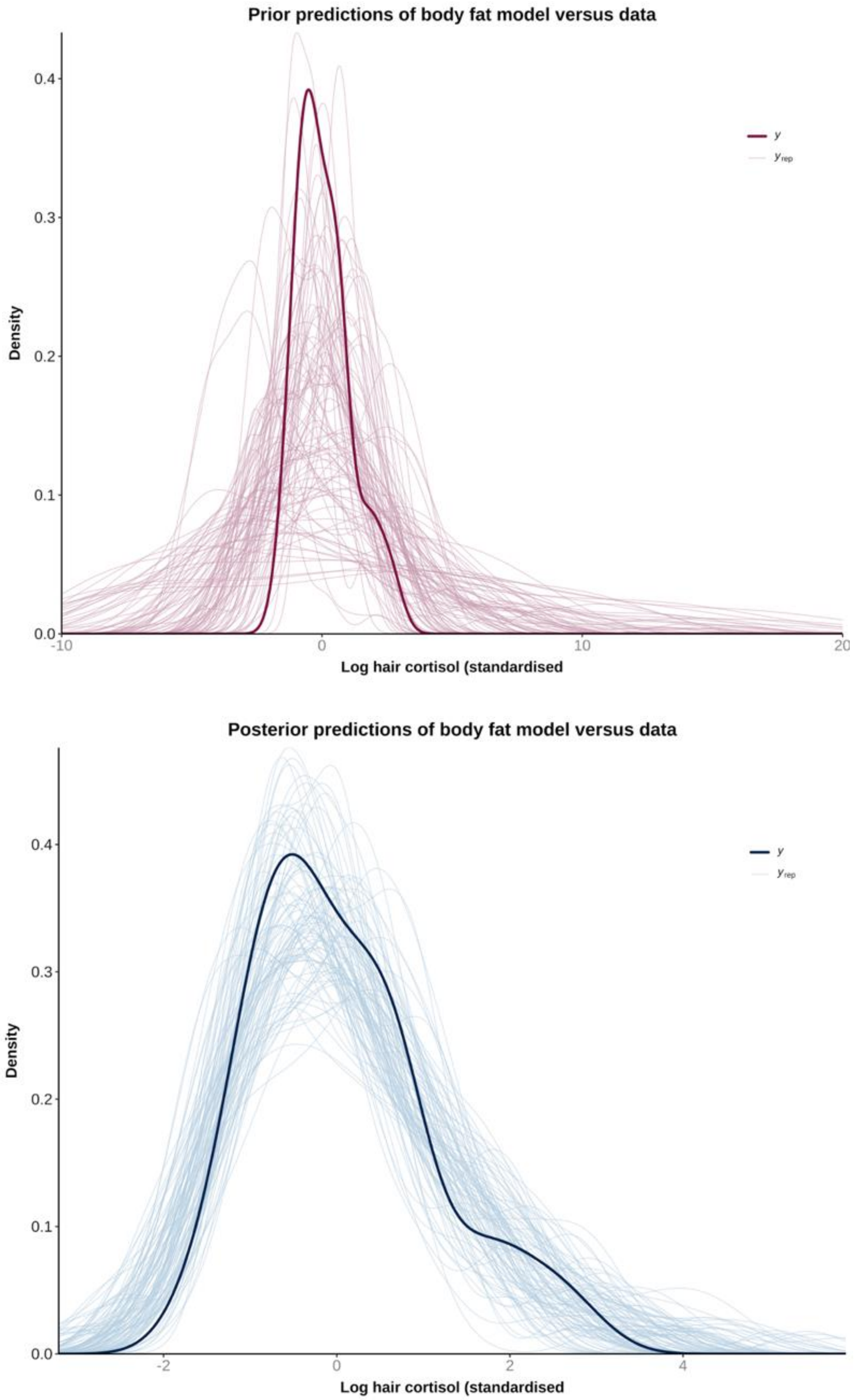

Fig 6. Distributions of many replicated data sets (thin feint curves) drawn from the prior (top; pink) and posterior (bottom; blue) predictive distribution, and compared with the empirical distribution of the observed outcome (thick dark curves).

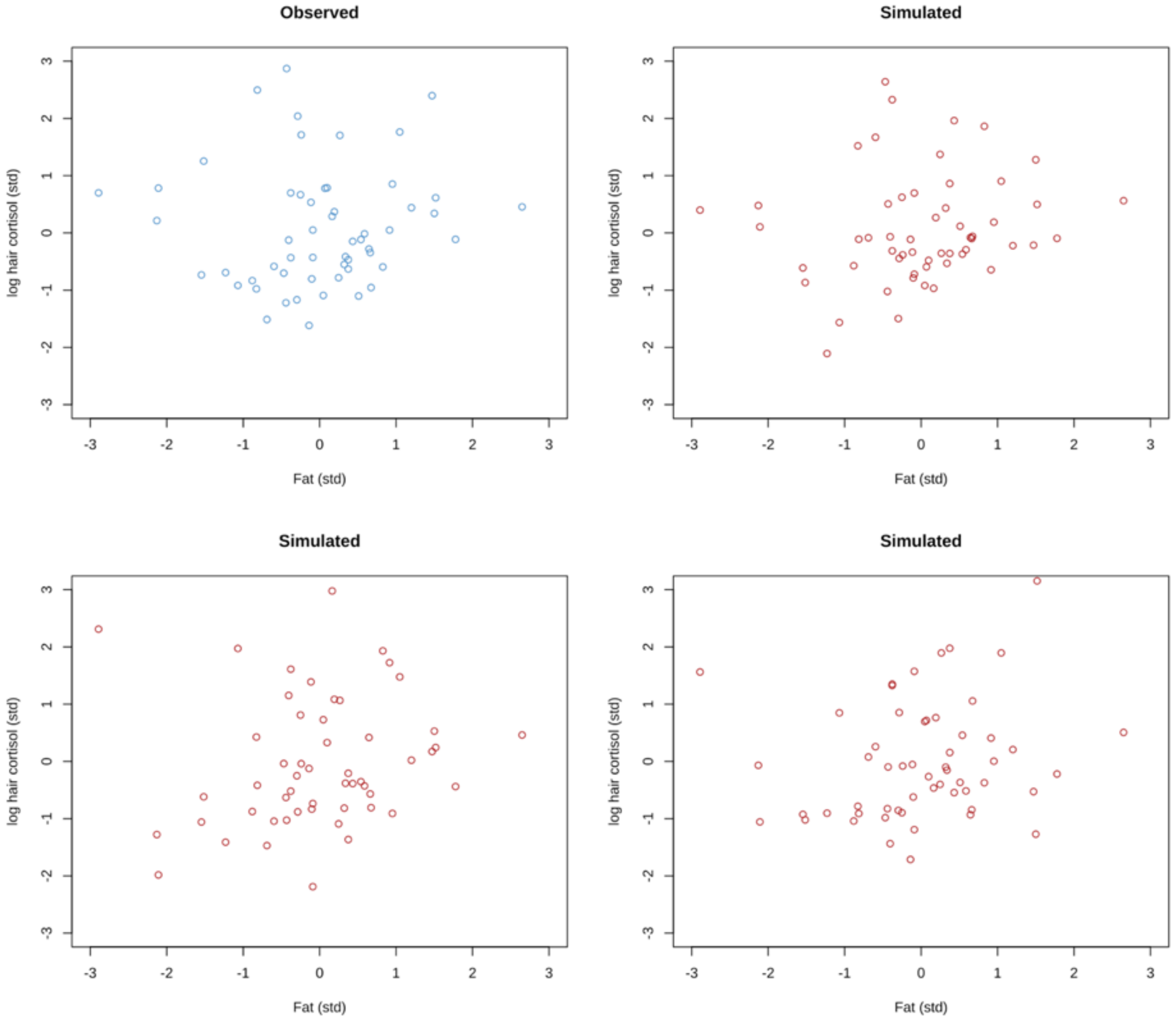

Fig 7. Scatter plots of body fat (standardised) vs. log hair cortisol concentration comparing observed data (blue) with simulations using draws from the posterior probability distributions of the final model. The data distribution in the scatterplots generated from simulated data are broadly similar to that of the observed data.
