# Supplementary material for: A Bayesian analysis of variables causally associated with hair cortisol concentration in dogs with obesity
Source: Front Vet Sci. 2025 Nov 27;12:1695345. doi: 10.3389/fvets.2025.1695345 (PMC12695548; doi:10.3389/fvets.2025.1695345)
Supplement: Supplementary file 4 [file Data_Sheet_4.pdf]

Supplementary File 4

Justification of choice of prior distributions

Prior distributions were selected for all parameters of each model, with the overall aim being to ensure they were weakly regularising, adjusted to ensure that that pre-data predictions would span the range of scientifically plausible outcomes. This was confirmed by graphical visualisations and prior predictive simulations from models that sampled from the prior probability distributions only (see below). Priors were occasionally adjusted to resolve issues with divergent transitions or potential prior-likelihood conflicts as described in the main manuscript.

Intercept and sigma parameters

For the intercept of all models, a neutral, regularising prior was chosen (normal, mean 0, SD 0.5) whilst, for most models, an exponential distribution (rate 1) was chosen for the sigma parameter to ensure that values were always positive and were more likely to be smaller. The main exception was for models that included comorbidity, either as the predictor or an adjustment variable; there was a difference the variance of hair cortisol concentrations between dogs that did or did not have a comorbidity (manuscript Fig 3) and, as a result, sigma was instead estimated as a parameter within the model, using comorbidity as a single predictor variable.

Likelihood function

The likelihood function chosen for all models (besides the reverse causality model) was a skew-normal distribution (see above); in addition to mean and sigma parameters of a normal distribution, requires a shape parameter (alpha), which required its own prior to be specified. For this, a mean value of 4 was chosen because graphical simulations suggested that it approximately replicated the shape of the logHCC data (manuscript Fig 2); this mean value was coupled with a SD of 2 to ensure that this prior was weakly regularising. All models also required a prior for the grouping variable (random effect, the individual study number) to be set, since significant variability amongst dogs was expected, a neutral, weakly-regularising prior (normal, mean 0, sigma 1) was again used.

Body fat percentage

There were no previous studies in dogs assessing the effect of body fat percentage on hair cortisol concentration, but human studies were available. In one study, logHCC was greater in humans with obesity (0.98 pg/mg) compared with those of normal weight (0.86 pg/mg) and in overweight condition (0.90 pg/mg) (Jackson et al, 2017), suggesting a slight (approximately +0.10 SD unit) positive effect of body fat mass on hair cortisol concentration. Against this, however, the effect of BCS were assessed in a previous study of dogs living in a Nicuaguan reserve, and suggested a slight (approximately -0.03 SD unit) negative effect on log hair cortisol (Bowland et al. 2020); however, the dogs were described as living “in a marginal environment where disease, malnutrition, and

mortality rates are high" (Bowland et al., 2020) and, not surprisingly, most dogs were in underweight condition (mean BCS 2.3, range 1-6/9). Therefore, the effects on hair cortisol were more likely to be associated with poor nutrition and health rather than obesity. Further, no differences in HCC were observed before and after a 4-week intervention involving lead walking in dogs with obesity, albeit with the limitation that changes in weight were modest to negligible (Kim et al. 2024). Therefore, given these inconsistent study findings, we opted for a neutral, weakly-regularising prior (mean 0, sigma 1), to enable the beta coefficient to be either positive and negative (manuscript Fig 4a), but to limit expected slopes for the regression line to within a sensible range (see results section for more details).

Comorbidity

Since it was plausible that having a comorbidity might increase HCC, given known mechanisms by which diseases might affect physiological stress in various species, including through infections, chronic pain and altered immune function (Knezevic et al., 2023; Alotibly et al;, 2024). However, there is limited research into the effects of disease on cortisol responses in dogs, with a single study of atopic dermatitis in dogs (Stuart Marques et al., 2023); in this study, HCC were used as a biological marker of stress, and declined by ~50% after therapy. Considering this limited evidence, we chose a marginally-positive, weakly-regularising prior (mean 0.25, sigma 1); this suggested a small, positive average (25% of a SD unit: logHCC 0.3; hair cortisol 4 pg/mg), but allowing for a wide range of positive or negative predictions (manuscript Fig 4b).

Sex

Regarding the prior for sex, previous studies in other species have revealed conflicting results, with a positive effect of male sex seen in one human study (Binz et al., 2018), but an opposite effect seen in vervet monkeys (Laudenslager et al., 2023). In dogs, female dogs had greater hair cortisol in one study (van den Laan et al., 2022), but no sex effect was seen in a second (Bowland et al., 2020). Given the variable and conflicting results in the literature, and the fact that all dogs were neutered, a neutral, weakly-regularising prior was chosen (mean 0.25, sigma 1), again enabling a wide distribution of positive and negative effects to be considered with no preference for either.

Age

There was not much evidence of an effect of age on HCC in the literature although, in one study, no effect was evident (Bowland et al., 2020). Therefore, once again, a neutral, regularising prior was chosen (normal distribution, mean 0, sigma 0.5); such a prior meant we expected the bulk of the probability distribution for the age effect to lie close to zero (limited effect), although both positive and negative effects were possible.

Breed

No previously-published studies on a possible effect of breed on HCC were identified, although breed effects might be plausible, either because of differences in hair colour (see below) or because of genetic differences in manifestation of stress across breeds. However, since we were not certain which breeds might be affected, we again chose a neutral regularising prior (normal distribution, mean 0, sigma 1.5 when used for the

causal effect; normal distribution, mean 0, sigma 1.5, when used in a adjustment set), which would enable a range of effects amongst different breed groups to be identified from the data.

Coat colour

In one study of hair coat colour, logHCC were greater in dogs with a light-coloured coat colour, compared with those whose coats were either a mixed or dark in colour (Bowland et al., 2020). However, the observed effects were relatively small equating to +0.070 SD (light versus mixed) or +0.075 SD (light versus dark) difference in log hair cortisol. Given that this was only a single study, light hair coat was set as the reference category and weakly-regularising priors, with a marginally-negative average effect, were chosen for both mixed (normal, mean -0.070, sigma 1) and dark (normal, mean -0.070, sigma 1) hair colour.

Season of sampling

There was little evidence of an effect of season on hair cortisol. In one previous study, cortisol concentrations were greater when hair was sampled in January, compared with sampling in either May or September (Roth et al., 2016). However, samples were not taken at other times making it difficult to relate these to sampling season, not least the effect of summer, of months other than the times sampled. As a result, a neutral weakly-regularising prior was preferred (normal, mean 0, sigma 1).

Reverse causality model

This model was used to check for possible reverse causality in our scientific model, with body fat as the outcome variable and log hair cortisol concentration as the causal predictor. Based on the DAG (see Supplementary file 1), the adjustment set required was age, sex, breed and comorbidity.

Likelihood function

The likelihood function chosen was a normal distribution, as this was the most appropriate for the data distribution of body fat percentage.

Intercept and sigma

For the intercept, a neutral, regularising prior was chosen (normal, mean 0, SD 0.5) whilst, for most models, whilst an exponential distribution (rate 1) was chosen for the sigma parameter to ensure that values were always positive and smaller values would be more likely.

Hair cortisol

The research on associations between body fat and hair cortisol are discussed above (see body fat percentage). As a result, we again decided to use a neutral regularising prior for log hair cortisol e.g., normal distribution (mean 0, SD 0.5).

Comorbidity

Some comorbidities present could be associated with increased body fat, e.g., osteoarthritis, on account of reduced physical activity. For others, we might expect a decrease in body fat, e.g. neoplasia, kidney disease, whilst we would not expect any

effect for many (e.g. skin disease etc). Therefore, safest to set a neutral, weakly-regularising prior for comorbidity effect, e.g. normal distribution (mean 0, SD 1). This would allow effects in either direction, but would still regularise the model to avoid overfitting.

Sex
We might expect a slightly negative effect of male sex on body fat mass. However, in this cohort, we had previously not found an effect of sex on body fat, probably because only very overweight dogs are referred to the specialist obesity care clinic. Therefore, we decided to set a neutral, regularising prior for male sex, e.g. normal distribution (mean 0, SD 1). This would again allow effects in either direction, but would still regularise the model to avoid overfitting.

Age
In dogs, body condition and body fat mass increase with age from young to middle age, but then decrease in later life. Therefore, it might be expected that a non-linear effect could be present, and modelling with splines might be necessary. However, this would increase the number of parameters in the model, and we were conscious that the dataset was small. Therefore, assuming the age effect to be linear was preferable. Given that either positive or negative effects were plausible, we decided on a neutral, regularising prior e.g. normal distribution (mean 0, SD 1), again to enable effects in either direction, but avoiding overfitting.

Breed
In large epidemiological studies, there are differences in obesity prevalence amongst breeds, with some breeds more prone to obesity than others. However, this was not a representative population… dogs attending a specialist obesity care clinic, and all would have excess body fat unless they are being assessed after weight loss. Also, most breed groups contained relatively small numbers, making it hard to discern changes. Finally, the effect in the 'other' group would be highly unpredictable given the diverse breed range. Again, therefore, we believed that the safest approach was to set a neutral, regularising prior, but allowing for wide variability amongst groups. The ultimate choice was normal distribution (mean 0, SD 2).

References

Alotiby A. Immunology of Stress: A Review Article. J Clin Med. 2024 Oct 25;13(21):6394. doi: 10.3390/jcm13216394

Binz TM, Rietschel L, Streit F, Hofmann M, Gehrke J, Herdener M, Quednow BB, Martin NG, Rietschel M, Kraemer T, Baumgartner MR. Endogenous cortisol in keratinized matrices: Systematic determination of baseline cortisol levels in hair and the influence of sex, age and hair color. Forensic Sci Int. 2018 Mar;284:33-38. doi: 10.1016/j.forsciint.2017.12.032

Bowland GB, Bernstein RM, Koster J, Fiorello C, Brenn-White M, Liu J, Schwartz L, Campbell A, von Stade D, Beagley J, Pomerantz J, González A, Quick M, McKinnon K, Aghaian A, Sparks C, Gross JB. Fur Color and Nutritional Status Predict Hair Cortisol Concentrations of Dogs in Nicaragua. Front Vet Sci. 2020 Oct 19;7:565346. doi: 10.3389/fvets.2020.565346

Jackson SE, Kirschbaum C, Steptoe A. Hair cortisol and adiposity in a population-based sample of 2,527 men and women aged 54 to 87 years. Obesity (Silver Spring). 2017 Mar;25(3):539-544. doi: 10.1002/oby.21733.

Kim K, Song B, Kim D, Kim DH, Lee HJ, Kim G. Effect of leash walking on weight loss and assessment of hair cortisol in overweight dogs. Comparative Exercise Physiology 2024, 20(3), 283-291. doi: 10.1163/17552559-20231019

Knezevic E, Nenic K, Milanovic V, Knezevic NN. The Role of Cortisol in Chronic Stress, Neurodegenerative Diseases, and Psychological Disorders. Cells. 2023 Nov 29;12(23):2726. doi: 10.3390/cells12232726

Laudenslager ML, Jorgensen MJ, Fairbanks LA. Developmental patterns of hair cortisol in male and female nonhuman primates: lower hair cortisol levels in vervet males emerge at puberty. Psychoneuroendocrinology. 2012 Oct;37(10):1736-9. doi: 10.1016/j.psyneuen.2012.03.015

Roth LS, Faresjö Å, Theodorsson E, Jensen P. Hair cortisol varies with season and lifestyle and relates to human interactions in German shepherd dogs. Sci Rep. 2016 Jan 21;6:19631. doi: 10.1038/srep19631

Stuart Marques V, Calesso JR, de Carvalho OV, da Costa-Val Bicalho AP. Hair cortisol concentration, disease severity and quality of life in dogs with atopic dermatitis during lokivetmab therapy. Vet Dermatol. 2023 Aug;34(4):339-347. doi: 10.1111/vde.13151

van der Laan JE, Vinke CM, Arndt SS. Evaluation of hair cortisol as an indicator of long-term stress responses in dogs in an animal shelter and after subsequent adoption. Sci Rep. 2022 Apr 21;12(1):5117. doi: 10.1038/s41598-022-09140-w
