# Supplementary material for: A Bayesian analysis of variables causally associated with hair cortisol concentration in dogs with obesity
Source: Front Vet Sci. 2025 Nov 27;12:1695345. doi: 10.3389/fvets.2025.1695345 (PMC12695548; doi:10.3389/fvets.2025.1695345)
Supplement: Supplementary file 5 [file Data_Sheet_5.pdf]

Supplementary information: File 5

Analyses of the posterior distribution

Different graphical illustrations of the posterior distributions of the causal effects in the final models.

(a) A comparison of prior and posterior distributions. The thin blue line represents the density distribution, the thick vertical line represent the mean and the shaded blue region represents the central (50%) probability interval. Differences in in the shape and position of the posterior distributions, relative to the prior distribution, indicates the effect of the observed data on the predictions.

(b) Plots of the highest probability density interval (a.k.a. highest density interval) of the posterior distribution of the causal effect from each model. The y-axis depicts the density distribution, whilst the x-axis depicts possible values for the beta parameter of the causal effect. Different probability intervals are depicted by colour (red 65%; purple 70%; orange 80%; yellow 89%; green 95%; blue 100%). The feint vertical dotted line represents the

(c) Conditional effects of the causal variable from each model, depicted either as a linear prediction (solid line: median; shaded area: 97% uncertainty region of the effect) for continuous variables, or as a point (median) and whisker (97% uncertainty interval) plot for categorical variables.

1. **Visual illustrations of the effects of age on hair cortisol concentration**

(a)

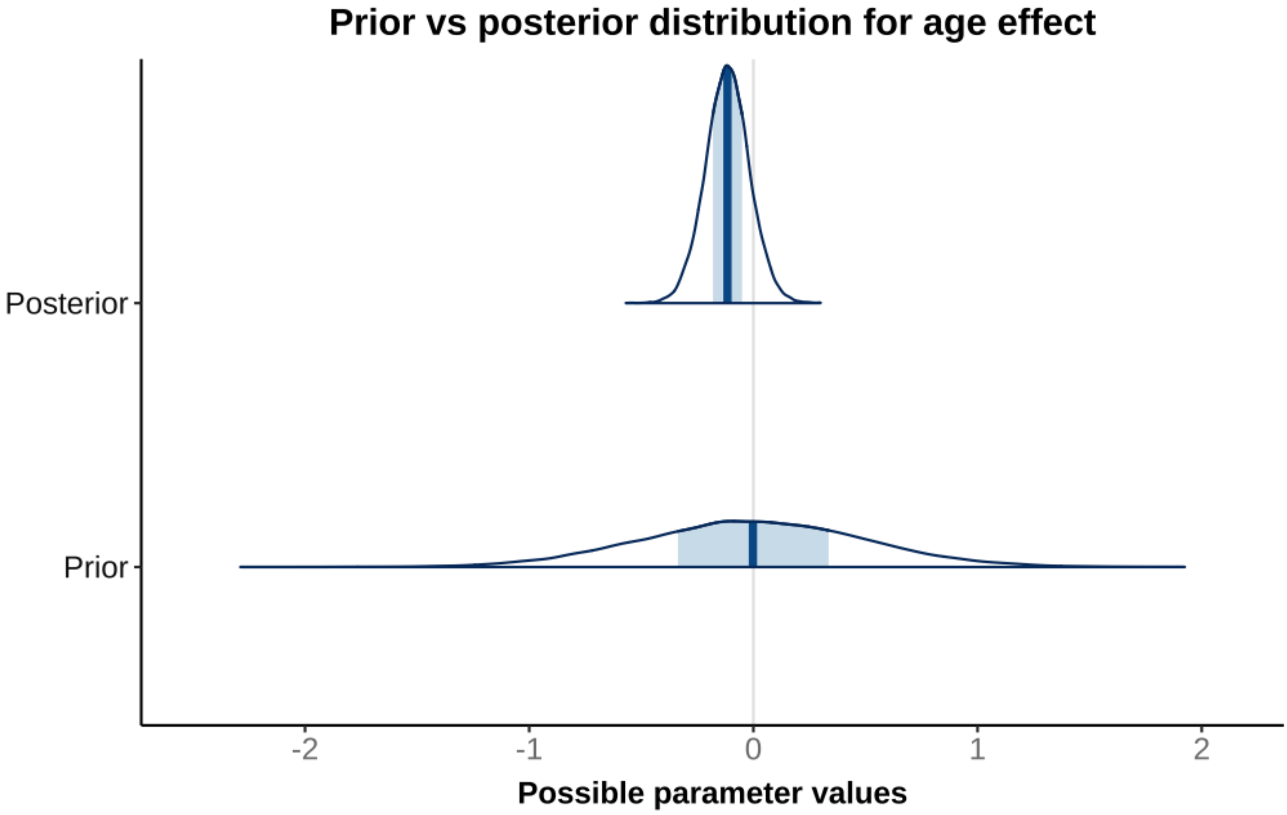

(b)

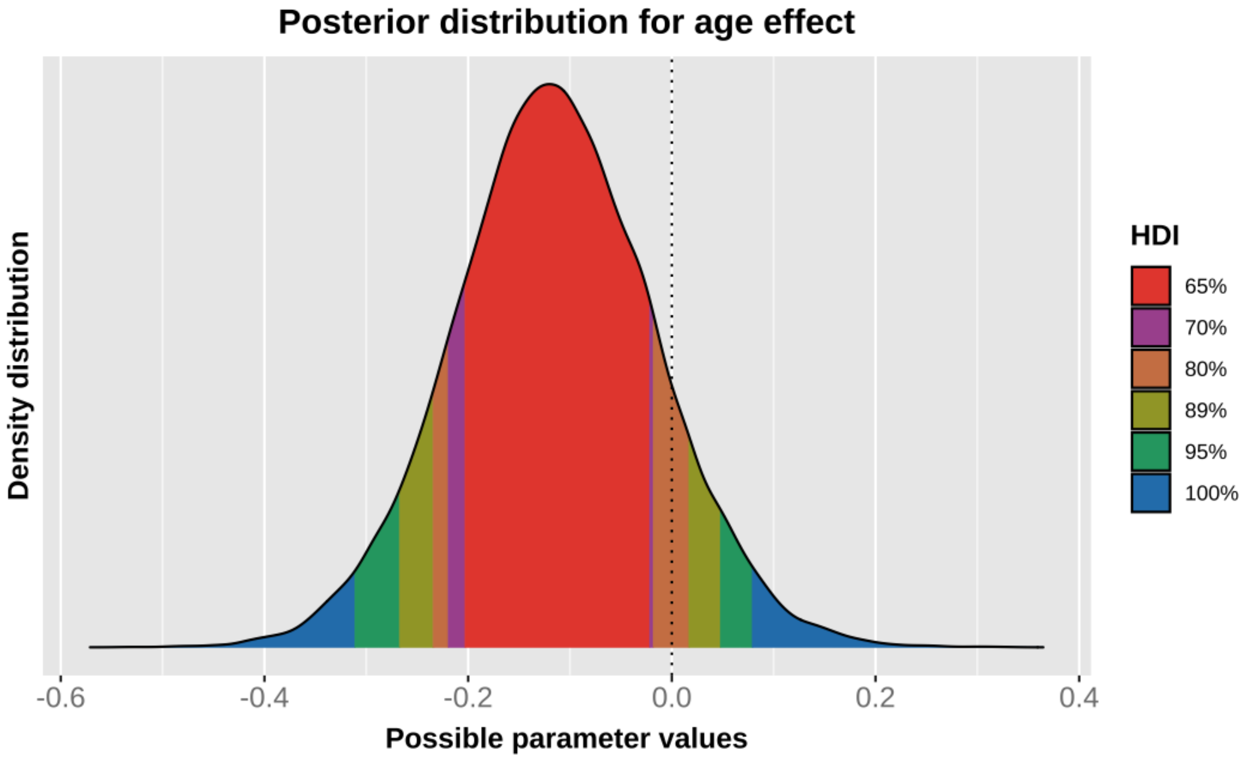

(c)

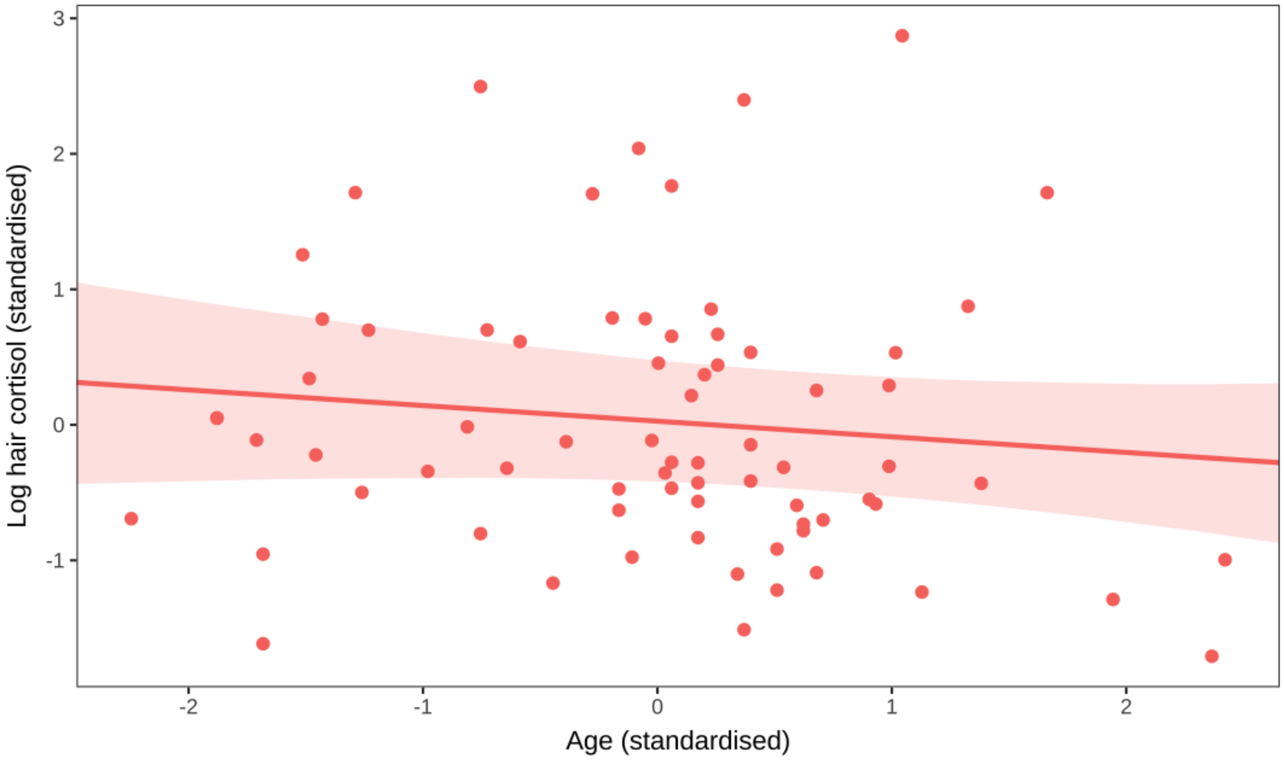

Log hair cortisol (standardised)
Age (standardised)
3
2
1
0
-1
-2
-1
0
1
2

2. **Visual illustrations of the effects of sex on hair cortisol concentration**

(a)

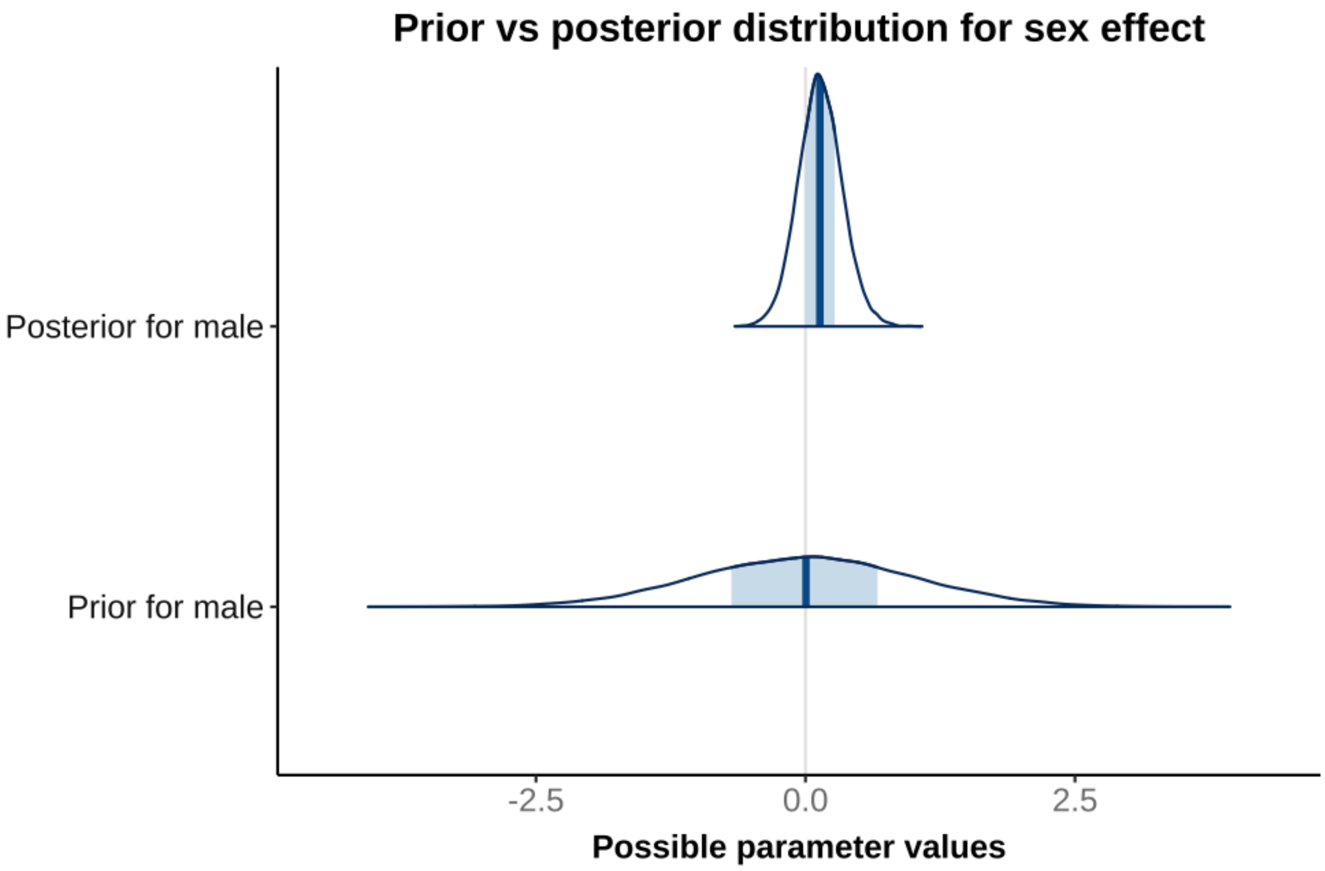

(b)

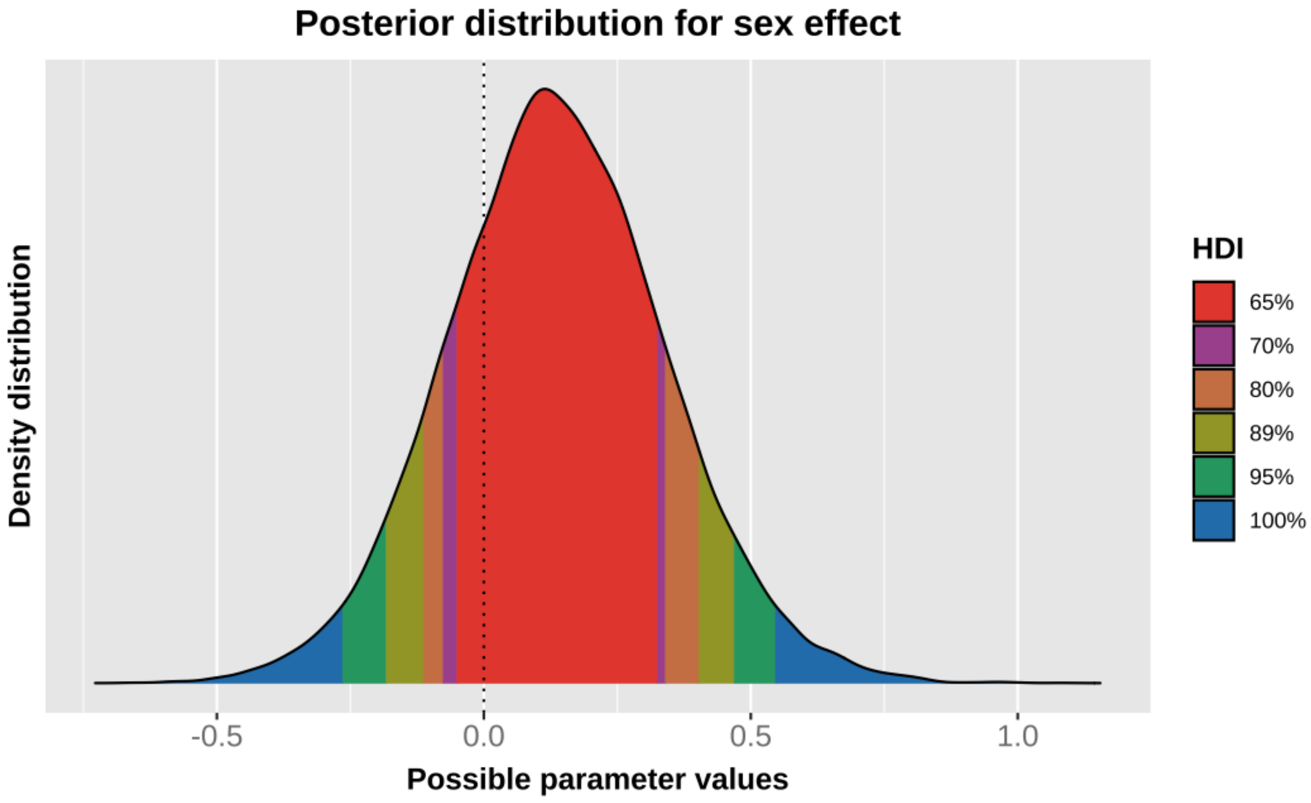

(c)

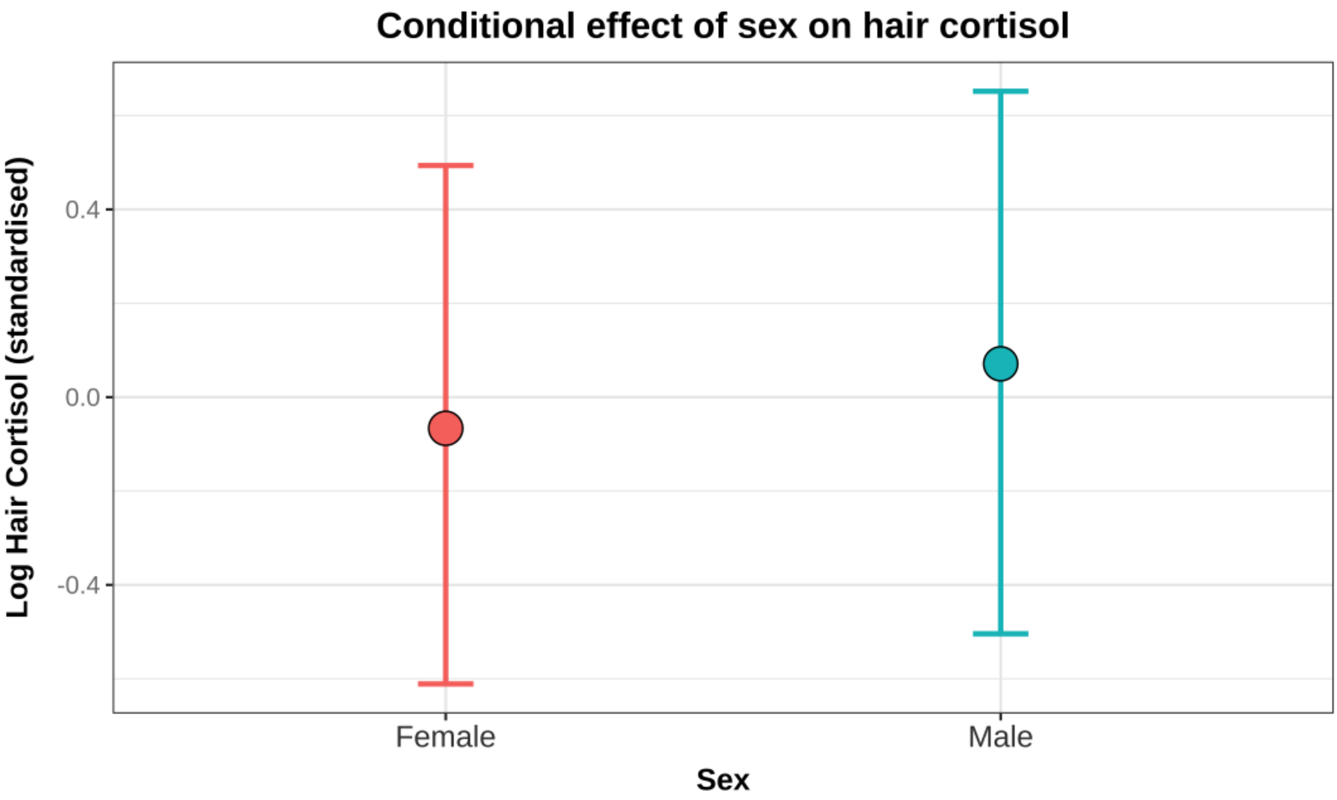
Conditional effect of sex on hair cortisol
Log Hair Cortisol (standardised)
0.4
0.0
-0.4
Female
Male
Sex

3. **Visual illustrations of the effects of breed on hair cortisol concentration**

(a)

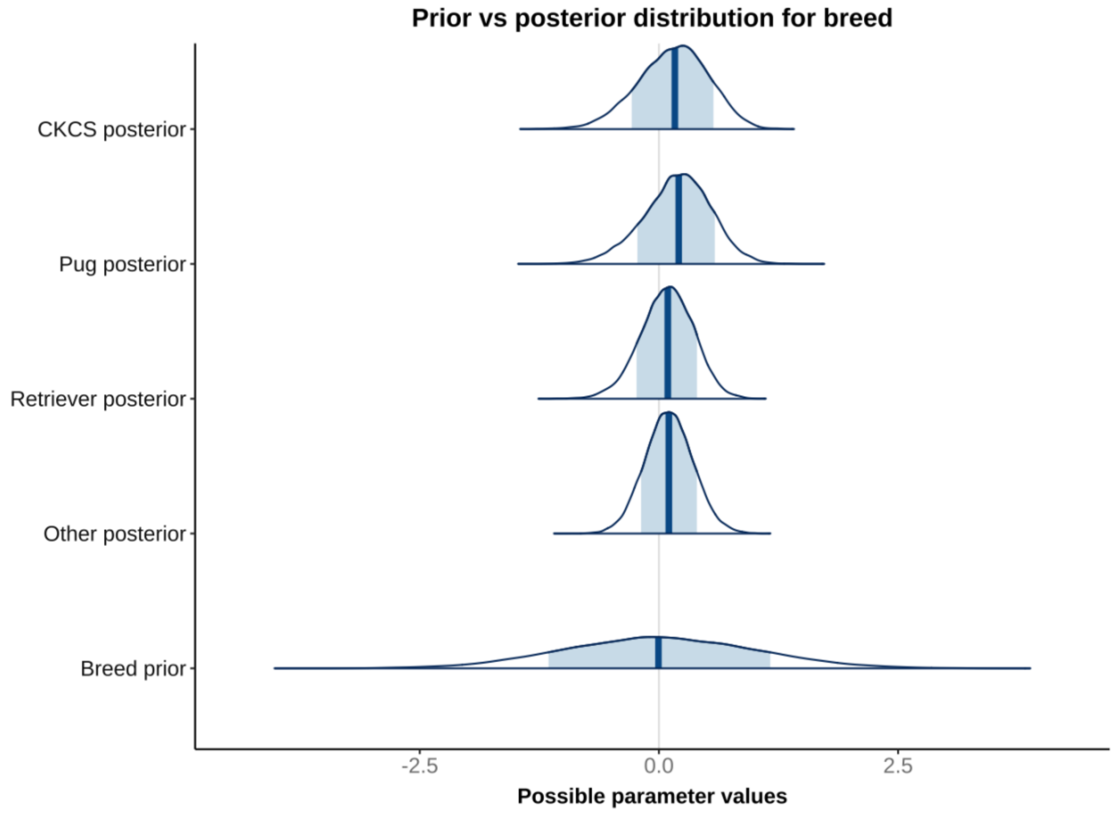

(b)

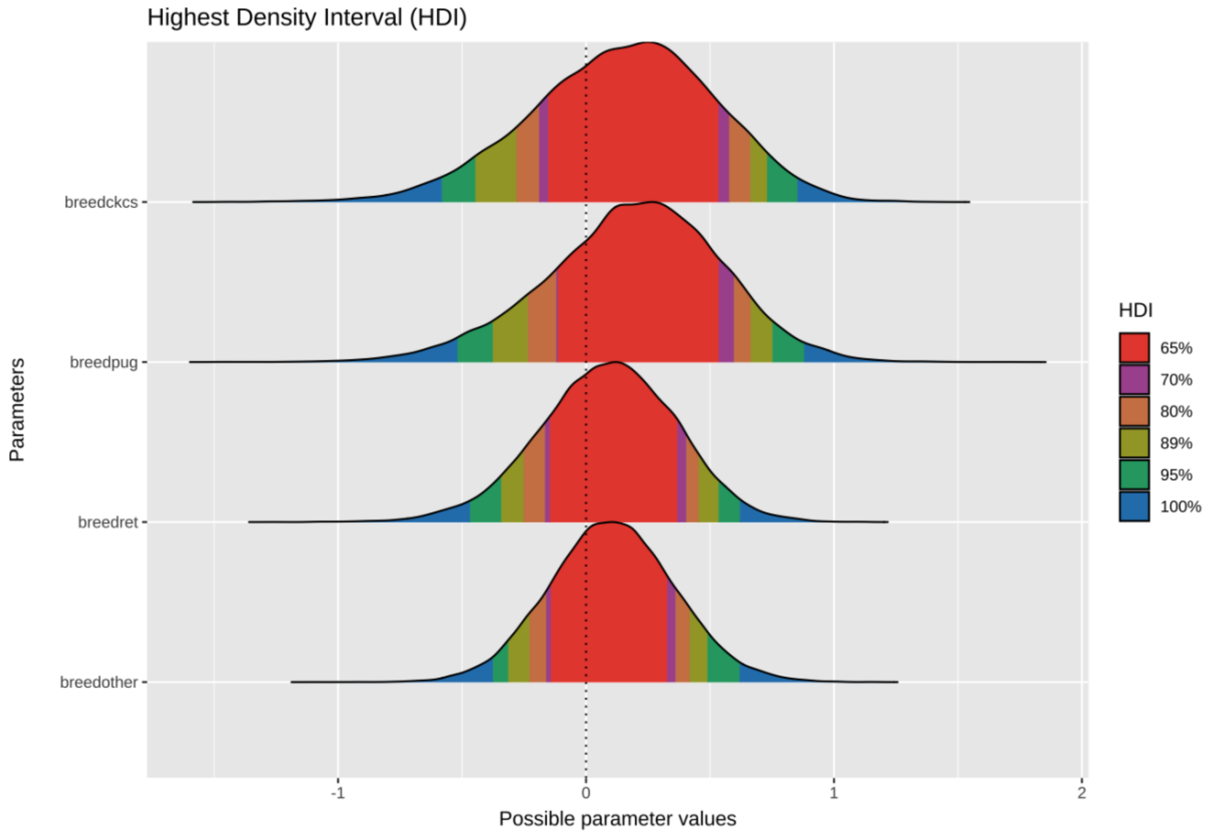

(c)

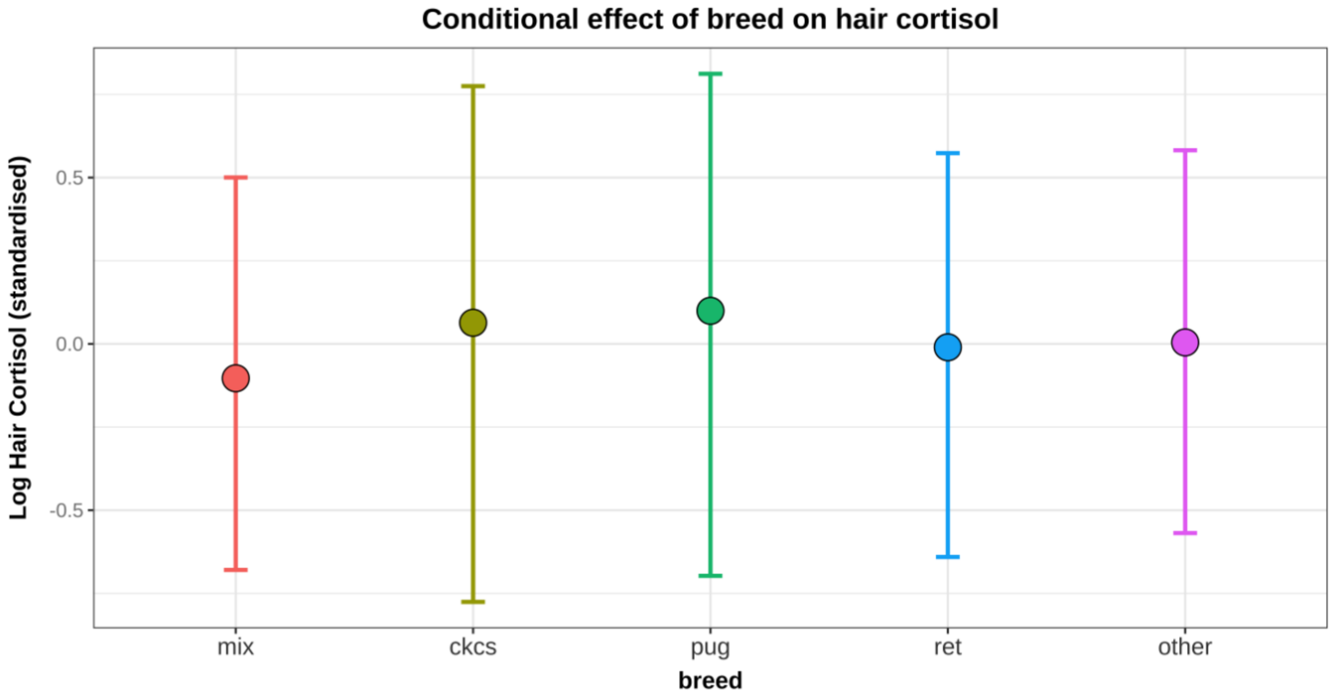
Conditional effect of breed on hair cortisol
Log Hair Cortisol (standardised)
0.5
0.0
-0.5
mix
ckcs
pug
ret
other
breed

4. **Visual illustrations of the effects of coat colour on hair cortisol concentration**

(a)

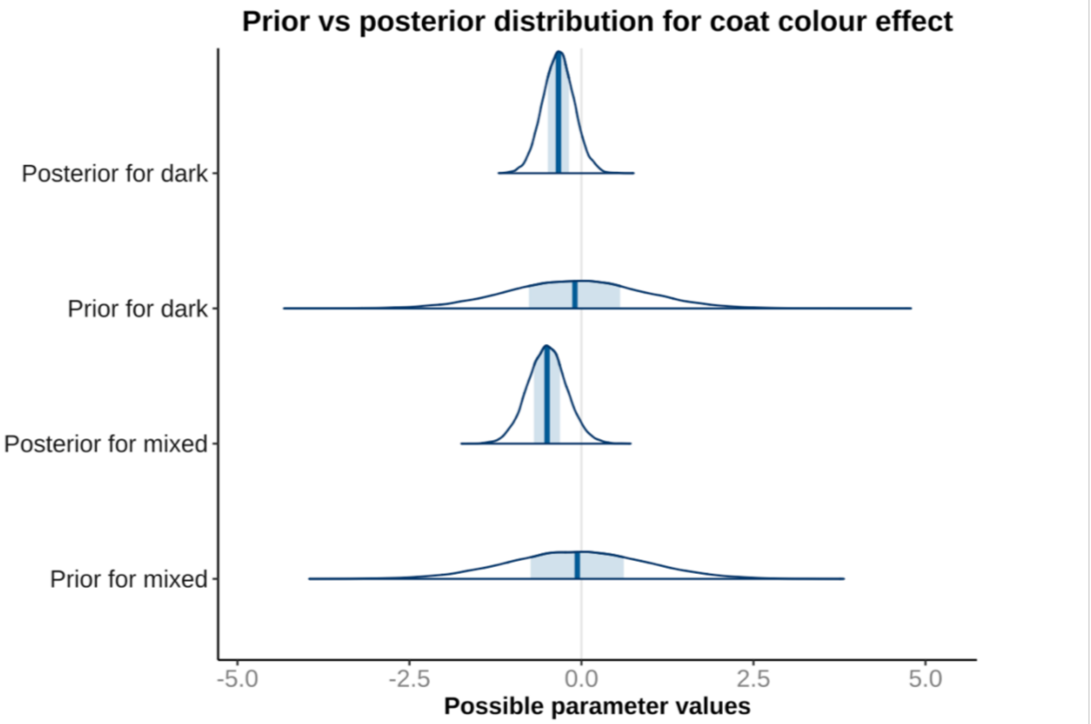

(b)

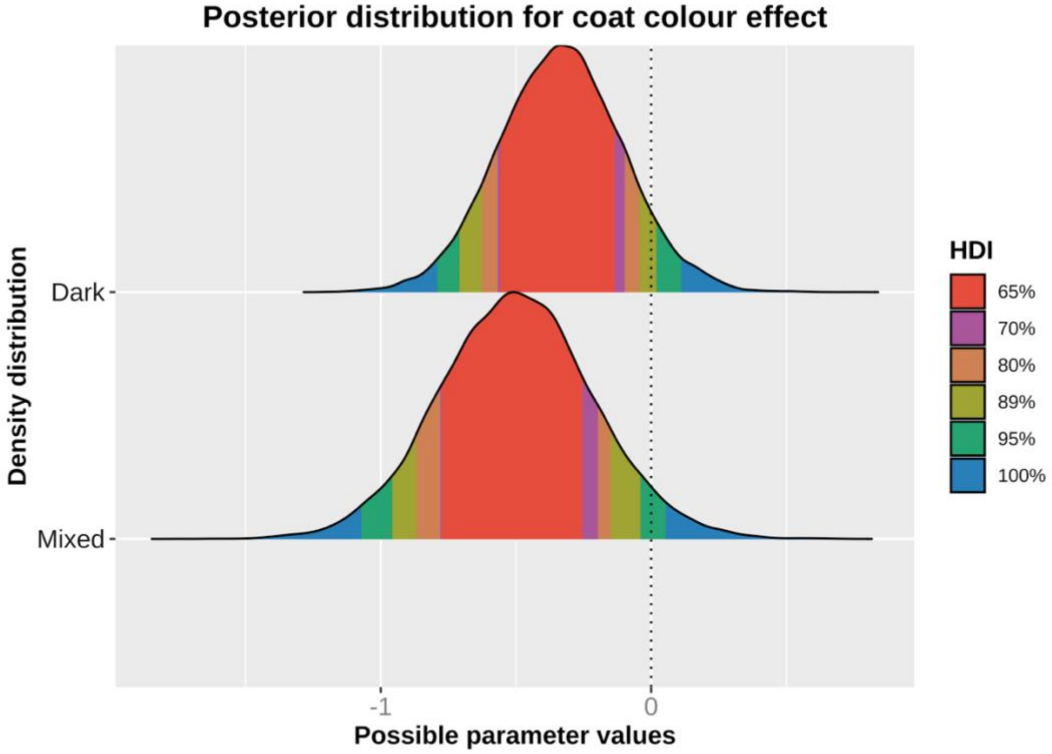

(c)

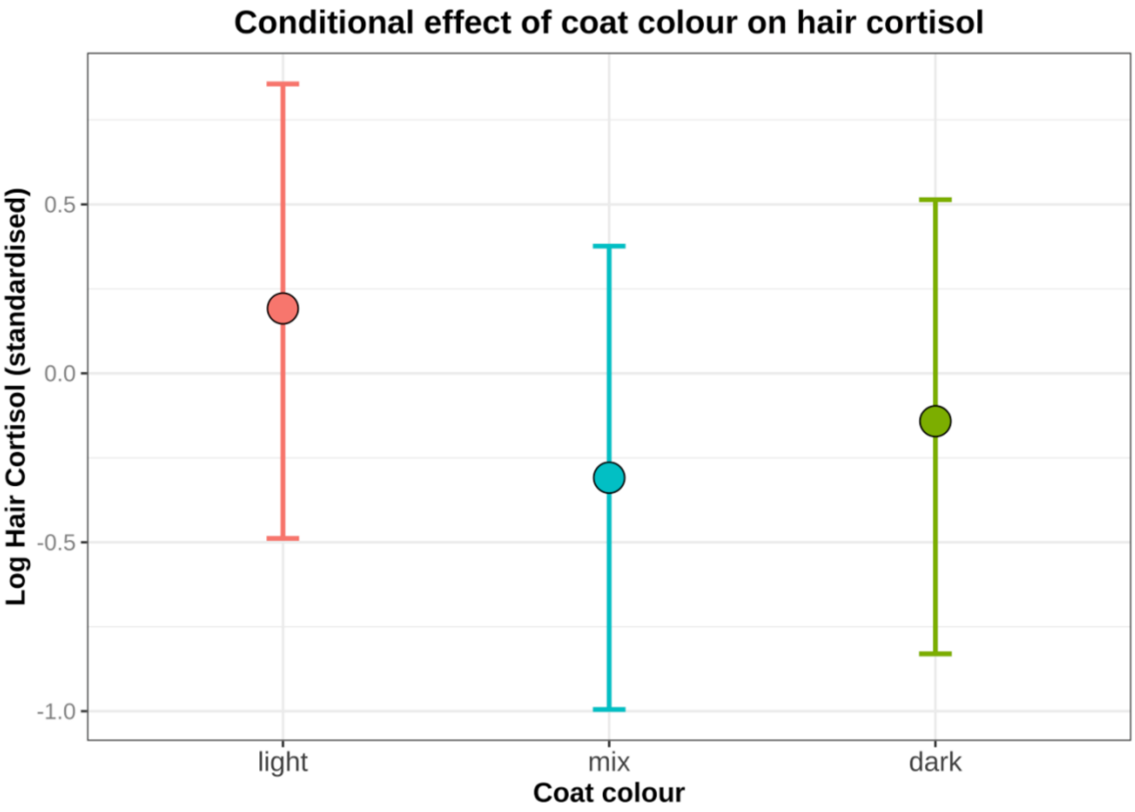

Conditional effect of coat colour on hair cortisol
Log Hair Cortisol (standardised)
0.5
0.0
-0.5
-1.0
light
mix
dark
Coat colour

5. **Visual illustrations of the effects of season on hair cortisol concentration**

(a)

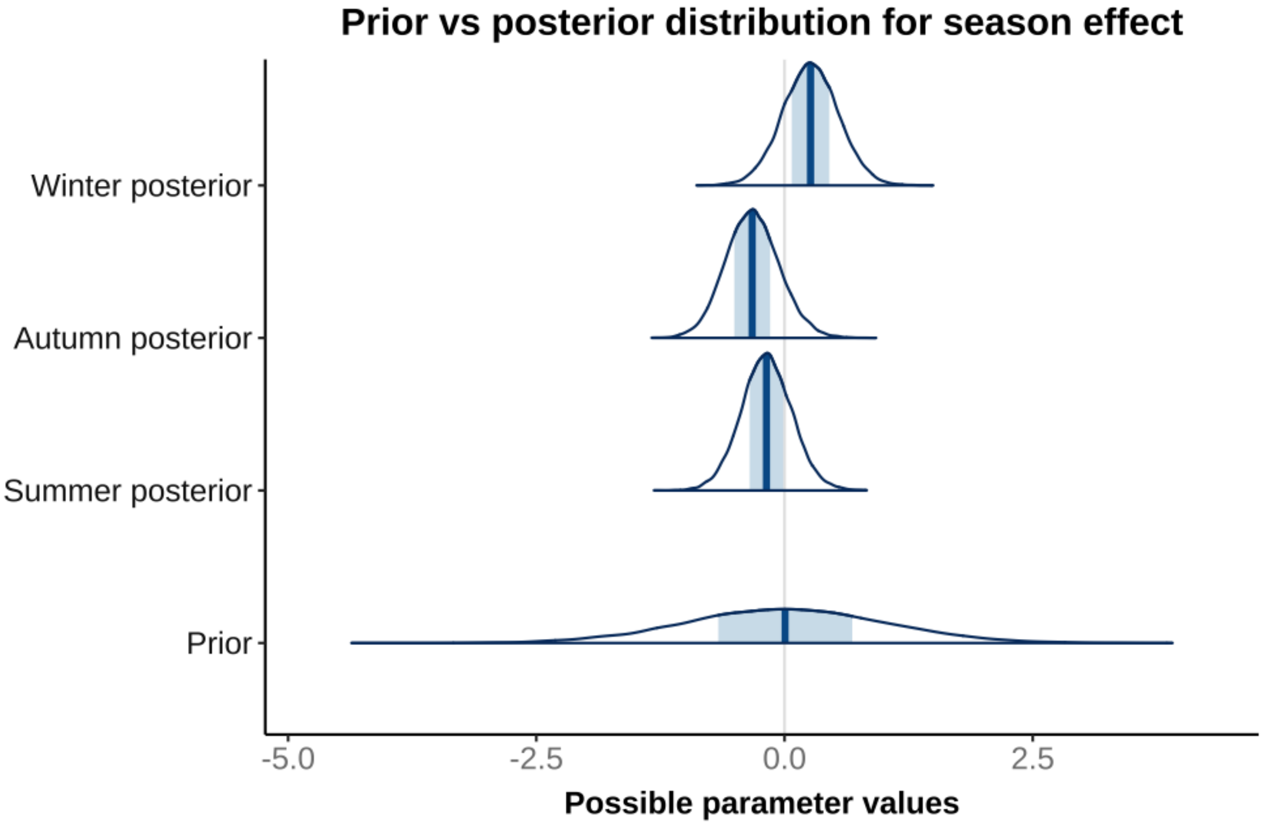

(b)

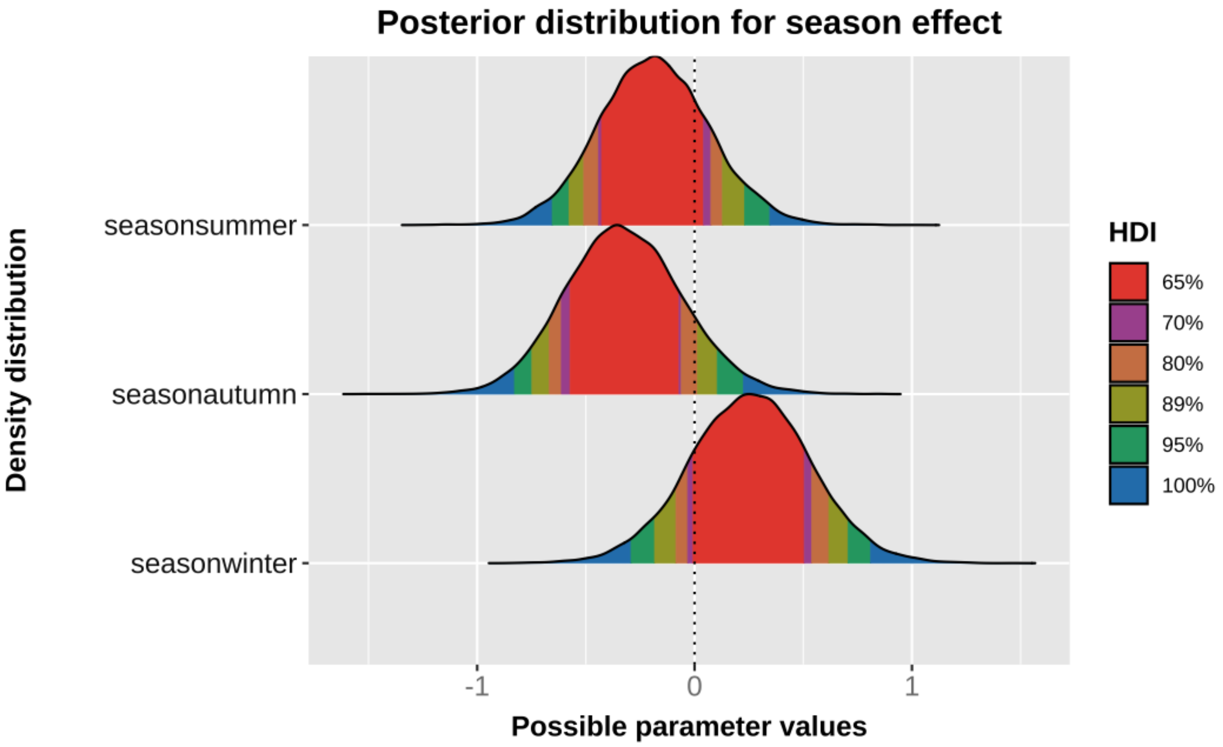

(c)

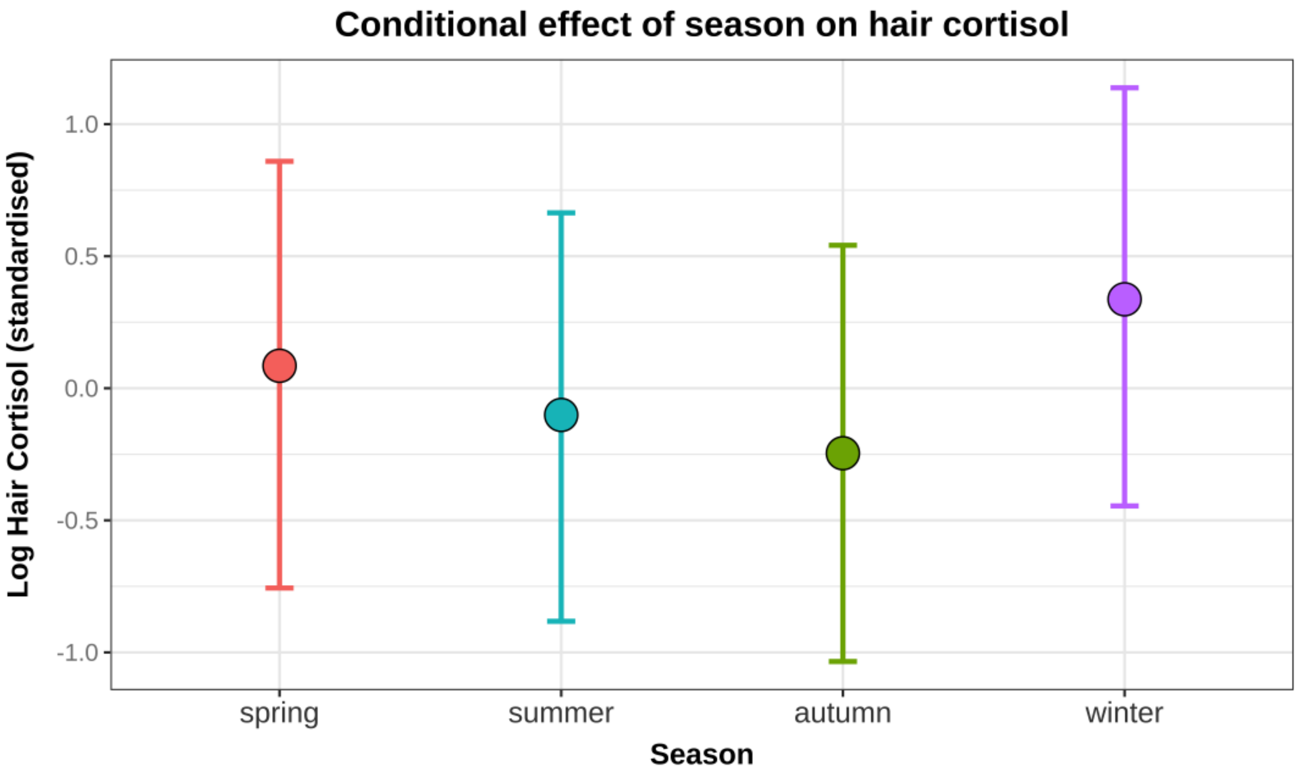
Conditional effect of season on hair cortisol
Log Hair Cortisol (standardised)
1.0
0.5
0.0
-0.5
-1.0
spring
summer
autumn
winter
Season
